# Trematode recolonization in first intermediate snail hosts after large-scale stream restoration

**DOI:** 10.1186/s13071-026-07583-y

**Published:** 2026-07-28

**Authors:** Annabell Hüsken, Jessica Schwelm, Bernd Sures

**Affiliations:** 1 Aquatic Ecology and Centre for Water and Environmental Research, University of Duisburg-Essen, Universitätsstraße 5, 45141 Essen, Germany; 2 Research Center One Health Ruhr, Research Alliance Ruhr, University of Duisburg-Essen, Universitätsstraße 5, 45141 Essen, Germany; 3 Water Research Group, Unit for Environmental Sciences and Management, North-West University, Potchefstroom Campus, Potchefstroom, 2520 South Africa

**Keywords:** Parasites, Gastropoda, Dispersal, Colonization, Succession, Life cycle

## Abstract

**Background:**

Parasite recolonization and succession following disturbance are inherently linked to the recovery of free-living species, yet patterns governing parasite recolonization in freshwater ecosystems remain largely unexplored. The Emscher River in western Germany was recently restored after a century of degradation as an open sewer system. As only few aquatic organisms persisted under these conditions, free-living organisms and parasites recolonized the system following restoration, providing a unique opportunity to study early successional dynamics.

**Methods:**

Trematodes infecting first intermediate snail hosts were surveyed through seasonal samplings during the initial years following restoration. Snails and trematodes were identified using combined morphological and molecular approaches. Successional dynamics of snail and trematode communities were analyzed on temporal and spatial scales using abundance, α-diversity (within-site species richness), and β-diversity (among-site dissimilarity). Trematode recolonization potential was assessed based on life-history traits, including dispersal capacity, life cycle complexity, and phylogenetic and geographic host specificity.

**Results:**

In total, 1511 snails of 12 species were examined, harboring 16 trematode species at an overall prevalence of 7.8%. The onset of trematode infections coincided with the establishment of native snail hosts, particularly *Ampullaceana balthica*, which harbored the highest trematode richness and overall prevalence. Although snail host abundance varied with season and distance to the river mouth, recolonization did not follow distinct spatial or temporal patterns. Both snail and trematode communities showed high spatial heterogeneity with pronounced species turnover between sites. Trematode assemblages were dominated by taxa from the regional species pool distributed via mobile definitive hosts. Species with truncated life cycles were underrepresented in terms of species richness but accounted for a high share of overall prevalence. High phylogenetic host specificity toward first intermediate hosts was observed, however, several species exhibited geographic host turnover among localities.

**Conclusions:**

These findings demonstrate rapid trematode recolonization following freshwater restoration, and suggest that early trematode assemblages show high spatial heterogeneity and are primarily shaped by host dispersal capacity and the regional species pool, rather than host specificity or life cycle complexity of trematodes. Continued monitoring will be required to assess whether directional changes in infection patterns emerge as the restored habitats mature.

**Graphical Abstract:**

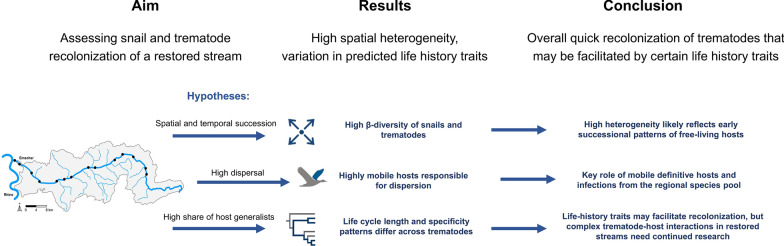

**Supplementary Information:**

The online version contains supplementary material available at 10.1186/s13071-026-07583-y.

## Background

Ecological succession describes the gradual change in species composition and community structure within an ecosystem over time, following disturbance or the creation of new habitats [1–3]. In freshwater ecosystems, disturbances initiating succession include natural processes such as floods or droughts, as well as anthropogenic alterations such as flow regulation, channelization, and chemical or organic pollution, all of which can substantially modify habitat conditions and community composition [4–6]. Importantly, the restoration of degraded freshwater ecosystems can likewise initiate succession, for instance by improving water quality or creating and increasing habitat heterogeneity [7]. Recolonization constitutes the earliest phase of succession, as species must first disperse into newly available habitats [8]. Recolonization and early succession are therefore influenced by habitat-specific characteristics such as environmental conditions and the surrounding species pool, and further shaped by species’ life-history traits, dispersal abilities, competitive interactions, and environmental filtering [1, 9]. Among free-living organisms, initial recolonization typically involves disturbance-tolerant, fast-dispersing species, followed by the establishment of more specialized taxa during early-, mid- and late-successional stages associated with ecosystem maturation [2, 3, 8, 10–12].

Parasites are intrinsically linked to the dynamics of free-living organisms, as they depend on the availability of suitable hosts for survival and transmission [13]. Consequently, parasite recolonization and succession in restored freshwater ecosystems is expected to broadly follow trajectories observed in free-living taxa, while simultaneously being shaped by species- and host-specific life-history traits that influence dispersal, establishment, and persistence in new habitats. First, dispersal capacity is generally expected to be higher in allogenic parasites, which cycle through both aquatic and terrestrial hosts, than in autogenic taxa that complete their life cycles exclusively within aquatic hosts [14–16]. As terrestrial hosts can disperse parasites among waterbodies, recolonization potential is likely to be higher in allogenic taxa, particularly those using highly mobile definitive hosts such as birds [17–19]. Second, host generalism is recognized as a key determinant of recolonization and establishment, as generalist parasites are more likely to encounter susceptible host species and thus persist following introduction into new habitats [20–23]. Third, parasites with simple life cycles are presumed more likely to establish and persist following introduction, as the probability of life cycle completion within the new habitat is comparatively high. In contrast, parasites with complex life cycles depend on the presence of multiple suitable hosts and thus stable biotic and trophic interactions developing over time [13, 24, 25].

Digenean trematodes are particularly well suited for investigating prerequisites and processes of parasite recolonization and succession in aquatic ecosystems, as they comprise both allogenic and autogenic species [26, 27]. Their complex life cycles typically involve aquatic gastropods as first intermediate hosts, a broad range of invertebrates and vertebrates as second intermediate hosts, and a vertebrate definitive host [27]. At each life cycle stage, trematodes can be classified as either host generalists infecting a range of hosts, or specialists restricted to one or few hosts [28]. While the typical trematode life cycle involves three successive hosts, several species exhibit truncated life cycles in which one or more hosts are omitted, with life cycle complexity potentially reflecting ecological conditions and host availability [29, 30].

**Fig. 1:**
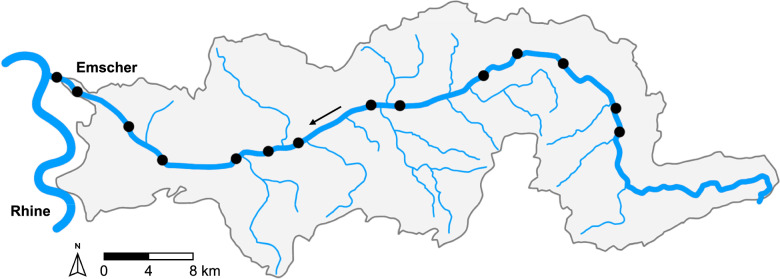
Map of the Emscher catchment showing sampling sites (black circles) and flow direction (black arrow)

The Emscher River drains one of Europe’s most densely populated regions in the Ruhr Metropolitan Area of western Germany, with a total stream length of 83 km and a catchment area of approximately 865 km2 (Fig. 1) [31]. Beginning with the region’s urbanization and industrial expansion in the early twentieth century, the Emscher and its tributaries were transformed into a heavily modified open sewer system with straightened channels and concrete beds [31]. For nearly a century, domestic and industrial wastewater was discharged directly into the river network, resulting in severe pollution and the near-complete loss of eukaryotic aquatic organisms, restricted to highly tolerant taxa such as oligochaetes [32]. In 1992, a large-scale restoration project was initiated with the aim of constructing underground sewers and returning the Emscher and its tributaries to a near-natural state [31]. Restoration measures included the removal of concrete beds, reversal of channelization and widening of stream profiles where possible. By the end of 2021, the Emscher was officially declared free of wastewater input, and in 2022 reconnection to the Rhine River restored longitudinal connectivity, enabling recolonization from both restored and unimpacted tributaries as well as upstream migration [31].

As communities of higher (i.e., metazoan) aquatic organisms were largely absent prior to restoration, the Emscher catchment provides a unique opportunity to study recolonization and successional dynamics among freshwater organisms. Accordingly, the aim of the present study was to investigate initial snail and trematode assemblages in the Emscher River. We hypothesized that abundance, species richness (α-diversity), and community composition (β-diversity) of snails and trematodes vary along spatial and temporal scales in the stream, reflecting successional processes associated with habitat recovery (H1). Based on the concepts of parasite recolonization outlined above, we further hypothesized that early trematode assemblages are dominated by allogenic taxa with high dispersal capacity (H2), taxa with obligatory or facultative life cycle truncation (H3), and/or host generalists toward first intermediate snail hosts (H4).

## Methods

### Field sampling and morphological analysis

Following an initial investigation of snails in the Emscher River in spring 2023, seasonal samplings were conducted in spring, summer, and autumn 2024 and 2025 at multiple sites along the main stem (Fig. 1, Additional file 1: Table S1). Snails were collected by hand or with hand nets from stones, fine sediments, submersed aquatic vegetation, and overhanging riparian vegetation. Abundance of snails per sampling site was recorded as the total number of snails sampled by one person within 30 min. Collected snails were initially identified based on shell morphology [33] and later verified molecularly. The snails were transported to the laboratory, separated in 20-mL beakers with filtered water from their respective sampling site, and placed under a light source to stimulate cercarial emergence from infected hosts. All beakers were examined daily for three consecutive days under a stereomicroscope for the presence of cercariae in the water column. Live cercariae were studied using an Olympus BX51 compound microscope (Tokyo, Japan) and microphotographs were taken with an UC30 digital camera (Tokyo, Japan) attached to the microscope. Subsequently, all snails were crushed between glass Petri dishes and dissected to detect prepatent (intramolluskan) trematode infections. Pooled trematode cercariae or parthenitae from individual infected snails as well as a small piece of foot tissue of selected snail specimens were fixed in 96% ethanol for molecular identification.

### Molecular and phylogenetic analysis

DNA of snail and trematode isolates was extracted using a salt precipitation protocol [34]. Molecular identification of snails was conducted on the mitochondrial cytochrome *c* oxidase subunit 1 (*cox*1) barcoding region. Molecular identification of trematode species was conducted on the nuclear D1–D3 region of 28S rDNA or the ITS1–5.8S–ITS2 region as well as a section of *cox*1 or the NADH dehydrogenase subunit 1 (*nad*1) mtDNA. Details on primer pairs and PCR thermocycling conditions are given in Additional file 1: Table S2. Amplified DNA was sent to Microsynth Seqlab (Germany) for purification and bidirectional Sanger sequencing using the PCR primers and additional sequencing primers for 28S rDNA.

Sequences were assembled and edited in Geneious Prime v. 2026.0.2 (https://www.geneious.com/), and subsequently compared to GenBank records using the BLASTn algorithm [35]. Trematode sequences were aligned with selected closely related reference sequences using MUSCLE v. 5.1 [36] (Additional file 1: Table S3). Mitochondrial *cox*1 and *nad*1 sequences were aligned with reference to the trematode mitochondrial code (https://www.ncbi.nlm.nih.gov/taxonomy/utils/wprintgc.cgi#SG21). All alignments were trimmed to the shortest sequence length. The best-fitting nucleotide substitution models were estimated using the corrected Akaike Information Criterion (cAIC) in ModelFinder [37] (Additional file 1: Table S4). Maximum likelihood (ML) phylogenetic trees with 1000 bootstrap replicates were generated using IQ-TREE v.2.4.0 [38]. Phylogenetic trees were visualized and edited using FigTree v. 1.4.4 (http://tree.bio.ed.ac.uk/software/figtree/) (Additional file 1: Figs. S1–S11).

### Data analysis

Data analysis was performed using RStudio v. 2024.12.0 based on R v. 4.4.2 [39, 40]. Figures were generated using *ggplot2* v. 3.5.1 [41]. Trematode species richness and prevalence were calculated following Bush et al. [42]. Overall prevalence was calculated as the sum of prevalences at a given scale (e.g., all allogenic taxa).

#### Successional dynamics of snails and trematodes

Successional dynamics of snails and trematodes (H1) were investigated at the levels of abundance, α-diversity (within-site species richness), and β-diversity (among-site dissimilarity). Snail metrics were quantified for each site × time combination, whereas trematode metrics were further quantified per host species (site × time × host species). However, as infections were almost exclusively limited to *Ampullaceana balthica* (Linnaeus, 1758), apart from two infections each in *Bithynia tentaculata* (Linnaeus, 1758) and *Stagnicola palustris* (O.F. Müller, 1774), all subsequent analyses were first conducted for pooled trematode infections regardless of host species and subsequently for *A. balthica* only to facilitate comparison at the host-level. Temporal analyses examined variation across sampling time points, years, and seasons, whereas spatial analyses examined longitudinal variation along the stream as well as distance decay in community composition with increasing distance between sites [43, 44].

Temporal and spatial variation in abundance and species richness (α-diversity) was analyzed using generalized linear mixed-effect models (GLMMs) with the *glmmTMB* package v. 1.1.11 [45]. Snail species richness was modeled using a Poisson error distribution with a log link, and snail abundance using negative binomial error distributions. For trematodes, species richness and overall prevalence (all trematode species combined) were modeled using Poisson and binomial error distributions, respectively. Trematode models were restricted to the years 2024–2025 as no infections were recorded in 2023. Fixed effects included year, season, and distance from the river mouth (km), and trematode models additionally included snail abundance and species richness as fixed effects. For all models, random intercepts were included to account for repeated sampling of sites, and Tukey-adjusted pairwise comparisons were performed between seasons using *emmeans* v. 1.11.0 on the response scale [46]. Model diagnostics and multicollinearity among predictors were assessed using *DHARMa* v. 0.4.7 [47] and *performance* v. 0.13.0 [48]. Coefficients of determination (R2) were calculated using *MuMIn* v. 1.48.11 [49]. Model outputs are presented in Additional file 2: Tables S5–S11.

To investigate temporal and spatial variation in snail and trematode community composition, β-diversity was quantified using Jaccard dissimilarity based on presence-absence data [50]. First, multiple-site β-diversity was calculated separately for each time point using *betapart* v. 1.6.1 [51], ranging from 0 for identical species composition among sites to 1 for complete dissimilarity. Total β-dissimilarity (β_JAC_) was further partitioned into turnover (β_JTU_), which assesses the extent of dissimilarity originating from species replacement among sites, and nestedness-resultant dissimilarity (β_JNE_), which assesses the extent to which species-poor communities represent subsets of species-rich communities [52]. Subsequently, pairwise dissimilarities were calculated for all site pairs sampled within each time point. Pairwise dissimilarities were then modeled as a function of time and river distance between sites to assess temporal changes and distance decay in community composition. Site pair was included as a random effect to account for repeated measurements of the same site combinations across sampling events. Snail communities were analyzed using linear mixed-effects models (LMMs) implemented in *lme4* v. 1.1.37 [53] and *lmerTest* v. 3.1.3, [101] whereas trematode communities were modeled using GLMMs with a beta error distribution and a logit link implemented in *glmmTMB*. Model predictions and pairwise comparisons were obtained using *emmeans*. Model diagnostics were assessed as described above. For all models, fixed effects were evaluated using Satterthwaite-adjusted *t*-tests for LMMs and Wald *z*-tests for GLMMs.

#### Life history traits of trematode taxa

Trematode life cycles were reconstructed based on published literature (H2, H3). Host specificity (H4) was calculated at both regional and global scales and included phylogenetic and geographic host specificity. For regional analyses, trematode–host data from the present study were combined with records from the Boye catchment, a tributary of the Emscher River [54]. To investigate host specificity on the global level, molecularly confirmed records of conspecific trematodes from additional localities were identified using a GenBank search and preferably matched based on mitochondrial DNA markers (*cox*1, *nad*1) (Additional file 2: Table S12). For each conspecific record, the snail host species and locality information were extracted from the corresponding source study to characterize the molecularly confirmed first intermediate host range of each trematode species. *Cox*1 sequences of these snail hosts were compiled and aligned together with sequences generated in the present study to reconstruct a host phylogenetic tree (Additional file 2: Fig. S17). Phylogenetic host specificity (PD) was then calculated as the total branch length of all infected hosts for each trematode taxon along the host phylogenetic tree using *picante* v. 1.8.2 [55–57]. To account for differences in host richness among taxa, standardized effect sizes of PD (SES-PD) were calculated based on 1000 replications, with SES-PD values < 0 indicating hosts are more closely related than expected by chance and values > 0 indicating hosts are more distantly related than expected by chance [56]. To facilitate comparison, trematode taxa were subsequently grouped as phylogenetic specialists (SES-PD ≤ − 1), generalists (SES-PD ≥ 1), or intermediate (see Additional file 2: Tables S13–S14). Geographic host specificity (β-specificity) was based on host turnover among geographic locations and quantified using multi-site Jaccard dissimilarity, ranging from 0 (identical host use across locations) to 1 (complete host turnover) using *betapart*. To facilitate comparison, trematode taxa were classified into geographic host specialist, intermediate, and generalist categories based on tertiles of β-specificity values (Additional file 2: Tables S13–S14).

## Results

### Identification and occurrence of snails and trematodes

Comparison of snail *cox*1 sequences with the NCBI database confirmed the morphological identification of all species. Representative sequences were deposited in GenBank with accession numbers PZ235457–PZ235483. In total, 1511 snails representing 12 species from five families were collected: Bithyniidae (1 species), Lymnaeidae (3), Physidae (1), Planorbidae (6), and Tateidae (1) (Table 1). The most abundant snail species in the catchment was *A. balthica* (*n* = 1091), followed by *Physella acuta* (Draparnaud, 1805) (*n* = 223), and *S. palustris* (*n* = 46). Patent or prepatent trematode infections were detected in 117 snails, corresponding to an overall prevalence of 7.8%. Trematode infections were restricted to *A. balthica* (overall prevalence of 10.3%), *B. tentaculata* (9.5%), and *S. palustris* (4.3%) (Table 1).

**Table 1:** Trematode species and prevalence per snail species from this study

Snail family and species (*n*)	Trematode family and species	Prevalence % (*n* infected)	Accession numbers		2nd int. host group (literature)	Definitive host group (literature)	Life cycle length	Reference
			28S/ITS1–5.8S–ITS2^a^	*cox*1/*nad*1^b^				
Bithyniidae Gray, 1857								
*Bithynia tentaculata* (Linnaeus, 1758) (*n* = 21)	Lecithodendriidae							
	*Lecithodendrium linstowi* (Dollfus, 1931)	4.8 (1)	PX972837	PX970447	Aquatic invertebrates	Bats	3	[97]
	Notocotylidae							
	*Notocotylus* sp. OK-2019 sensu [34]	4.8 (1)	PX972842	/	None (encyst externally)	Birds, mammals	2^d^	[34]
Lymnaeidae Rafinesque, 1815								
*Ampullaceana balthica* (Linnaeus, 1758) (*n* = 1091)	Cephalogonimidae							
	*Cephalogonimus* sp. EM-2024 sensu [58]	2.4 (26)	PX972818– PX972819	PX970442–PX970443	Amphibians	Amphibians	3(2)^c^	[58]
	Echinostomatidae							
	*Echinoparyphium recurvatum* (von Linstow, 1873)	2.1 (23)	PX972823–PX972828	PX993635^b^–PX993641^b^	Aquatic invertebrates	Waterfowl	3	[88]
	*Echinostoma revolutum* (Froelich, 1802)	0.4 (4)	PX972829–PX972832	PX993642^b^–PX993645^b^	Aquatic invertebrates	Waterfowl	3	[88]
	*Hypoderaeum conoideum* (Bloch, 1782)	0.6 (7)	PX972833–PX972836	PX993646^b^–PX993650^b^	Aquatic invertebrates	Waterfowl	3	[98]
	*Petasiger phalacrocoracis* (Yamaguti, 1939)	0.4 (4)	PX972844–PX972846/PZ225369^a^, PZ225375^a^–PZ225376^a^	/	Fishes	Fish-eating birds	3	[99]
	Notocotylidae							
	*Notocotylus* sp. AK-2017 sensu [54]	2.2 (24)	PX972838– PX972841	/	None	Waterfowl	2^d^	[59]
	Plagiorchiidae							
	*Plagiorchis* sp. 2 sensu [59]	0.1 (1)	PX972848	PX970449	Aquatic invertebrates	Unknown	3	[59]
	*Plagiorchis* sp. 3 sensu [59]	0.1 (1)	PX972849	PX970450	Aquatic invertebrates	Unknown	3	[59]
	*Plagiorchis* sp. 7 sensu [59]	0.3 (3)	PX972850– PX972852	PX970451–PX970453	Unknown	Unknown	3	[59]
	*Plagiorchis vespertilionis* (Müller, 1780)	0.1 (1)	PX972853	PX970454	Aquatic invertebrates	Bats	3	[93]
	Strigeidae							
	*Cotylurus* sp. lineage II sensu [60]	0.3 (3)	PX972820–PX972822	PX970444–PX970446	Aquatic invertebrates	Waterfowl	3	[60]
	*Australapatemon* sp.	0.9 (10)	PX972814–PX972817/PZ225368^a^, PZ225370^a^–PZ225374^a^	PX970438–PX970441	Aquatic invertebrates	Waterfowl	3	[100]
	Telorchiidae							
	*Opisthioglyphe ranae* (Frölich, 1791)	0.5 (5)	PX972843	/	Amphibians	Amphibians	3(2)^c^	[88]
*Lymnaea stagnalis* (Linnaeus, 1758) (*n* = 1)	–	–	–	–	–	–	–	–
*Stagnicola palustris* (O. F. Müller, 1774) (*n* = 46)	Plagiorchiidae							
	*Plagiorchis elegans* (Rudolphi, 1802)	2.2 (1)	PX972847	PX970448	Aquatic invertebrates	Birds, mammals	3	[88]
	Telorchiidae							
	*Opisthioglyphe ranae* (Frölich, 1791)	2.2 (1)	PV248753^e^	/	Amphibians	Amphibians	3(2)^c^	[88]
Physidae Fitzinger, 1833								
*Physella acuta* (Draparnaud, 1805) (*n* = 223)	–	–	–	–	–	–	–	–
Planorbidae Rafinesque, 1815								
*Ancylus fluviatilis* (O. F. Müller, 1774) (*n* = 1)	–	–	–	–	–	–	–	–
*Bathyomphalus contortus* (Linnaeus, 1758) (*n* = 10)	–	–	–	–	–	–	–	–
*Gyraulus albus* (O. F. Müller, 1774) (*n* = 21)	–	–	–	–	–	–	–	–
*Planorbarius corneus* Linnaeus, 1758 (*n* = 4)	–	–	–	–	–	–	–	–
*Planorbis carinatus* O. F. Müller, 1774 (*n* = 7)	–	–	–	–	–	–	–	–
*Planorbis planorbis* (Linnaeus, 1785) (*n* = 19)	–	–	–	–	–	–	–	–
Tateidae Thiele, 1925								
*Potamopyrgus antipodarum* (Gray, 1843) (*n* = 49)	–	–	–	–	–	–	–	–

Second intermediate and definitive host data were obtained from literature and were used to classify trematode species into second intermediate and definitive host groups. Life cycle length is given as the number of hosts needed for life cycle completion. Uninfected snail species are indicated with a dash (–), unsuccessful amplification of genetic markers is indicated with a forward slash (/)

^a^ITS1–5.8S–ITS2 sequences

^b^*Nad*1 sequences

^c^Facultative life cycle truncation

^d^Obligate life cycle truncation

^e^Published in [58]

Integrating morphological and molecular analyses, 16 trematode species belonging to seven families were identified (Table 1).

Cephalogonimidae: the newly generated 28S and *cox*1 sequences obtained from *A. balthica* were identical to isolates of *Cephalogonimus* sp. EM-2024 sensu [58] ex *A. balthica*. In the 28S phylogeny, these isolates formed a strongly supported clade distinct from other members of the Cephalogonimidae (Additional file 1: Fig. S1). Due to the scarcity of mitochondrial reference data, no phylogeny was conducted for *cox*1 sequences.

Echinostomatidae: the newly generated 28S sequences depicted four taxa belonging to the genera *Echinoparyphium* Dietz, 1909, *Echinostoma* Rudolphi, 1809, *Hypoderaeum* Dietz, 1909, and *Petasiger* Dietz, 1909 (Additional file 1: Fig. S2). In the *nad*1 phylogeny, the novel sequences grouped together with *Echinoparyphium recurvatum* s.s. (von Linstow, 1873), *Echinostoma revolutum* (Froelich, 1802), and *Hypoderaeum conoideum* (Bloch, 1782) with strong support (Additional file 1: Fig. S3). *Nad*1 amplification was unsuccessful for the isolates of genus *Petasiger*, thus species identification was based on 28S and ITS1–5.8S–ITS2 data. The newly generated 28S sequences were identical to each other and differed by 0–0.2% (0–2 nt) from published sequences of *Petasiger phalacrocoracis* (Yamaguti, 1939) (Additional file 1: Fig. S2). The newly generated ITS1–5.8S–ITS2 sequences were identical to each other and grouped together with published sequences of larval and adult *P. phalacrocoracis* with strong support (difference of 0–0.1% and 0–1 nt) (Additional file 1: Fig. S4).

Lecithodendriidae: the newly generated 28S sequence obtained from *B. tentaculata* was identical to published isolates of *Lecithodendrium linstowi* (Dollfus, 1931) from intermediate hosts and definitive bat hosts from Germany, Ireland, Ukraine, and the UK (Additional file 1: Fig. S5). The newly generated *cox*1 sequence was identical to isolates of *L. linstowi* ex *B. tentaculata* from Germany [54]. Due to the scarcity of reference data, no phylogeny was conducted for *cox*1 sequences.

Notocotylidae: based on the newly generated 28S sequences, two species of the genus *Notocotylus* Diesing, 1839 were identified. The isolate obtained from *B. tentaculata* was identical to *Notocotylus* sp. OK-2019 sensu [34] from *B. tentaculata* in Germany, and grouped together with isolates of *Notocotylus* sp. ex *B. tentaculata* from Ireland and *Anas platyrhynchos* (Linnaeus, 1758) from Russia. The isolates obtained from *A. balthica* were identical to *Notocotylus* sp. AK-2017 sensu [59] and clustered together with an unidentified species ‘Pronocephaloidea sp.’ ex *Potamopyrgus antipodarum* (Gray, 1843) from USA (difference of 0.1% and 1 nt) (Additional file 1: Fig. S6). Amplification of *cox*1 fragments for the isolates of family Notocotylidae was unsuccessful.

Plagiorchiidae: the newly generated 28S sequences depicted five species of the genus *Plagiorchis* Lühe, 1899 obtained from *A. balthica* and *S. palustris* (Additional file 1: Fig. S7). In the *cox*1 phylogeny, the isolates obtained from *A. balthica* grouped in strongly supported clades with larval isolates of *Plagiorchis vespertilionis* (Müller, 1780) from Germany and the Czech Republic, and *Plagiorchis* sp. 2, *Plagiorchis* sp. 3, and *Plagiorchis* sp. 7 sensu [59] from the Czech Republic, Iceland, Ireland, and Norway. The isolate obtained from *S. palustris* grouped together with larval isolates of *Plagiorchis elegans* (Rudolphi, 1802) from Ireland, Slovakia and the Czech Republic (Additional file 1: Fig. S8).

Strigeidae: the newly generated 28S sequences depicted two species of the genera *Cotylurus* Szidat, 1928 and *Australapatemon* Sudarikov, 1959 (Additional file 1: Fig. S9). In the *cox*1 phylogeny, the isolates of the genus *Cotylurus* clustered with larval and adult isolates from Japan, Poland, and the Czech Republic, corresponding to *Cotylurus* sp. lineage II sensu [60] (Additional file 1: Fig. S10). Published reference sequences for the *cox*1 sequences of the genus *Australapatemon* did not support species-level identification (closest reference sequence: *Australapatemon* sp. Lineage 9 C sensu [61], difference of 6.8% and 23 nt). Amplification of additional *cox*1 fragments for comparison with further published records of *Australapatemon* spp. was unsuccessful [62, 63]. Thus, identification of the novel isolates was based on 28S and ITS1–5.8S–ITS2 sequences. The 28S sequences were identical to each other and to sequences of adult *Australapatemon burti* ex *Anas diazi* (Ridgway, 1886) from Mexico as well as larval *Australapatemon* cf. *burti* and *Australapatemon* sp. ex *Planorbella* spp. from USA (Additional file 1: Fig. S9). The novel ITS1–5.8S–ITS2 sequences differed by 0–0.08% (0–1 nt) from each other and formed a well-supported clade with *Australapatemon* sp. ex *A. balthica* from Denmark (difference of 0–0.08% and 0–1 nt) (Additional file 1: Fig. S11). In the absence of further *cox*1 data for species-level refinement, the novel isolates were assigned to *Australapatemon* sp.

Telorchiidae: the 28S sequences obtained from *A. balthica* and *S. palustris* were identical to each other and to sequences of larval and adult isolates of *Opisthioglyphe ranae* (Frölich, 1791) from Germany and Ukraine. In the 28S phylogeny, these isolates clustered with strong support with sequences of *O. ranae* metacercariae from Russia (difference of 0.08–0.16% and 1–2 nt) (Additional file 1: Fig. S1). Amplification of *cox*1 sequences was unsuccessful, thus the isolates were assigned to *O. ranae* based on 28S data.

**Fig. 2:**
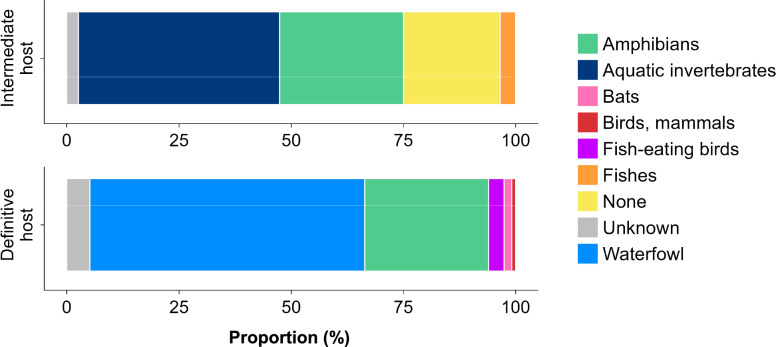
Proportion of second intermediate and definitive host groups of the trematode taxa identified in the Emscher River (based on literature data)

The most abundant trematode taxa were *Cephalogonimus* sp. EM-2024 sensu [54] (*n* = 26), *Notocotylus* sp. AK-2017 sensu [59] (*n* = 24), and *E. recurvatum* (*n* = 23). All remaining trematode taxa occurred in ≤ 10 individual snail hosts each (Table 1). Based on life cycle reconstructions, trematode taxa were predominantly introduced by waterfowl and amphibian definitive hosts, whereas the second intermediate hosts required for life cycle completion were predominantly aquatic invertebrates and amphibians (Fig. 2).

### Successional dynamics of snails and trematodes

**Fig. 3:**
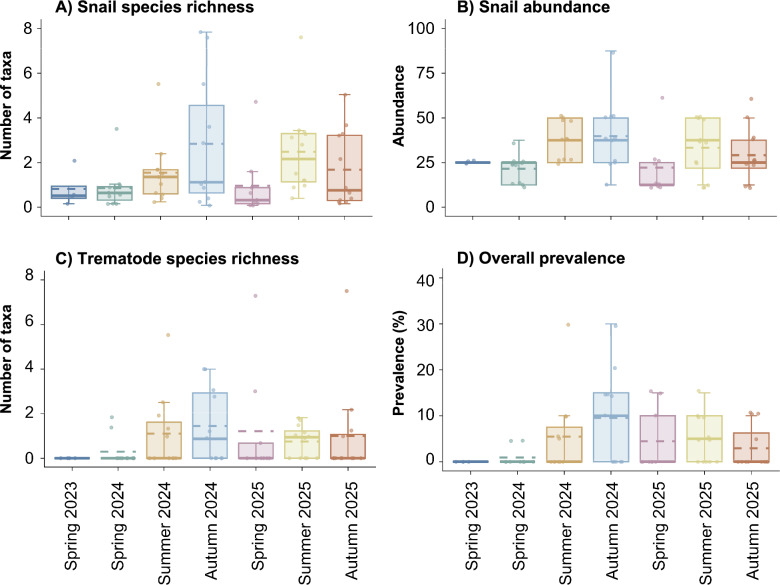
Species richness and total abundance of snails (**A**, **B**) and species richness and overall prevalence (%) of trematodes (**C**, **D**) in the first years following restoration in the Emscher River summarized for all sampling sites. Spring 2023 marked the onset of snails in the stream, whereas trematodes were first detected in spring 2024. Raw data points represent individual sampling sites, dashed lines indicate mean values

Initial snail samplings in spring 2023 consisted exclusively of the non-native species *P. acuta* and *P. antipodarum*, which harbored no trematode infections. No further samplings were conducted in 2023. In spring 2024, three additional snail species (*A. balthica, B. tentaculata, S. palustris*) were recorded, coinciding with the first detection of trematode infections in *A. balthica* (Fig. 3). Comparisons between pooled trematode data (all snail hosts combined) and host-specific analyses for *A. balthica* revealed no substantial differences in temporal or spatial distribution patterns. Therefore, subsequent results focus on pooled trematode data, while *A. balthica*-specific analyses are presented in Additional file 2. Host-specific occurrence and prevalence of individual trematode species over the study period are presented in Additional file 2: Fig. S16.

Once established, overall abundance and species richness of snails and trematodes did not show significant increases between the individual years following restoration (H1) (Fig. 3). Snail abundance exhibited pronounced seasonal variation (*χ*^2^ = 15.22, *df* = 2, *P* < 0.001), with significantly lower abundance in spring compared to summer (ratio = 0.414, *P* = 0.001) and autumn (ratio = 0.380, *P* = 0.004) (Fig. 3B). However, these dynamics were not mirrored in trematode assemblages, as no significant seasonal differences were detected in trematode species richness or overall prevalence (Fig. 3C, D). Site distance to the river mouth was no significant predictor among snail species richness, trematode species richness, and overall prevalence, but was positively associated with snail abundance (*z* = 3.21, *P* = 0.001) (Additional file 2: Table S5–S8, Fig. S12).

**Fig. 4:**
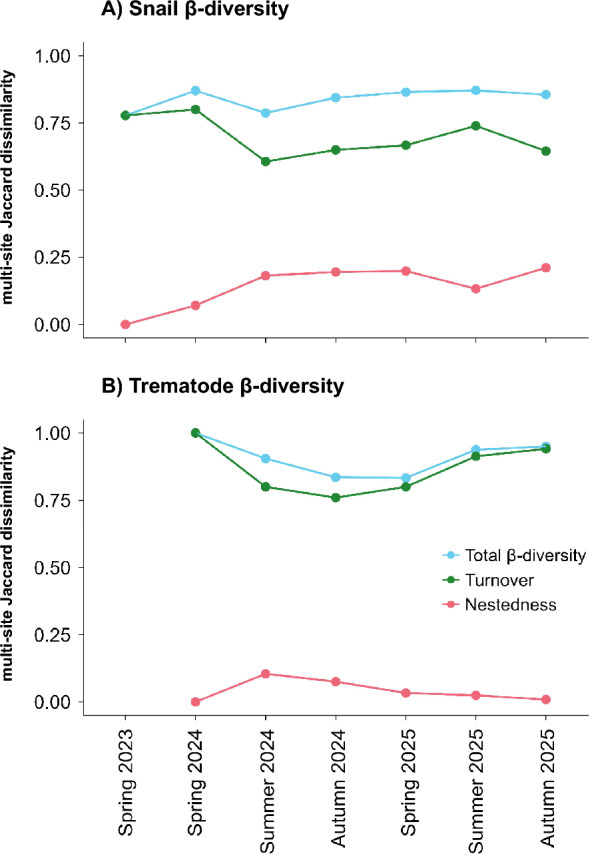
Temporal changes in community composition (β-diversity) of snails (**A**) and trematodes (**B**) based on multi-site Jaccard dissimilarity among sampling sites. Total β-diversity (β_JAC_) is partitioned into species turnover (β_JTU_), and nestedness-resultant dissimilarity (β_JNE_), where turnover reflects species replacement among sites and nestedness reflects the extends to which species-poor communities constitute subsets of species-rich communities

**Fig. 5:**
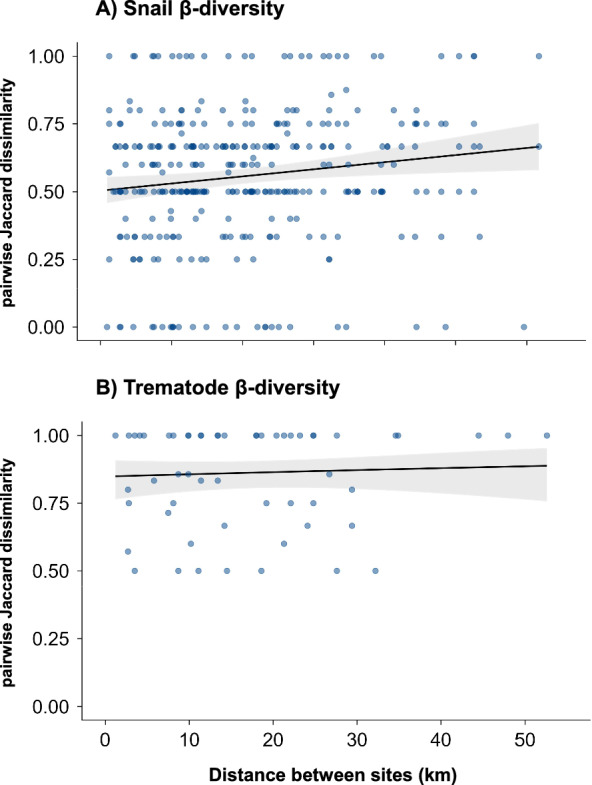
Distance decay of snail (**A**) and trematode (**B**) communities along the stream. Individual points represent pairwise Jaccard dissimilarity between sites within the same sampling time point. The solid line depicts predicted pairwise dissimilarity from mixed-effect models, with shaded areas indicating 95% confidence intervals. Snail communities exhibited significant distance decay (*t* = 2.65, *P* = 0.008), whereas no significant relationship was detected for trematode communities (*z* = 0.56, *P* = 0.578)

Both snail and trematode communities exhibited consistently high β-diversity over the sampling period (β_JAC_ snails = 0.78–0.87, β_JAC_ trematodes = 0.83–1), with species turnover (β_JTU_) contributing a higher share to differences among communities than nestedness-resultant dissimilarity (β_JNE_) (Fig. 4). Pairwise dissimilarity between sites (distance decay) increased significantly with river distance between sites (*t* = 2.65, *P* = 0.008) in snail communities, but did not change significantly over time (*t* = 0.09, *P* = 0.930). In contrast, trematode communities exhibited no significant relationship between pairwise dissimilarity and either river distance (*z* = 0.56, *P* = 0.578) or sampling time (*z* = 1.38, *P* = 0.168) (Fig. 5).

### Life history traits of trematode taxa

**Fig. 6:**
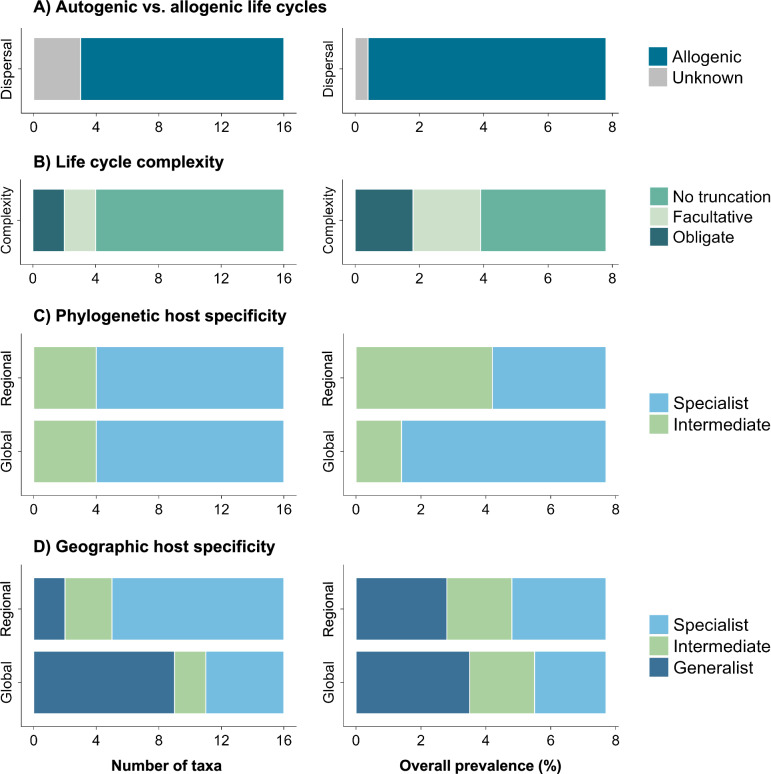
Number of taxa (left column) and overall prevalence (%, right column) of trematodes based on their life-history traits. For details on category assignment and classifications, see Additional file 2: Tables S13–S14

Trematode assemblages in the catchment were dominated by allogenic taxa (H2). Based on available life-history information, 13 of the 16 trematode taxa were classified as allogenic (7.4% of 7.8% total prevalence), whereas the remaining three taxa could not be classified as either allogenic or autogenic as their definitive hosts are currently unknown (Table 1, Fig. 6A).

Obligate or facultative life cycle truncation (H3) was observed in two taxa each, whereas the majority of trematode taxa exhibited complex three-host life cycles (Table 1). Accordingly, taxa with truncated life cycles were underrepresented in the dataset, while their overall prevalence was comparable to the pooled prevalence of taxa with three-host life cycles (Fig. 6B).

Phylogenetic host specificity (H4) toward first intermediate snail hosts was high at both regional and global scales, with most trematode taxa infecting one or few closely related host species, whereas only few taxa infected multiple snail genera or distantly related families (Fig. 6C, Additional file 2: Table S12). In contrast, β-specificity values reflected considerable geographic host turnover within the phylogenetic host ranges, particularly when considering host records beyond the scope of the present study, indicating that several of the identified taxa infect different but closely related snail species across localities rather than being restricted to a single host species (Fig. 6D, Additional file 2: Tables S13–S14).

## Discussion

The present study investigated recolonization and early successional dynamics of aquatic snails and trematodes following large-scale stream restoration. Trematode recolonization in first intermediate hosts was delayed, as initial snail communities consisted exclusively of uninfected non-native species, consistent with the commonly observed lack of trematode infections in invasive snails [64, 65]. The onset of trematode infections coincided with the establishment of native snails. *Ampullaceana balthica* harbored the highest trematode species richness and prevalence throughout the study period, highlighting its importance for trematode establishment during early succession in the system. Although *A. balthica* is highly susceptible to a range of trematode species, species richness and overall prevalence were low compared to previous studies [30, 54, 59, 66, 67], likely reflecting early successional dynamics in recovering free-living communities. Life cycle reconstructions further suggest that avian and amphibian definitive hosts were primarily responsible for introducing trematodes into the newly available habitats. Below, we discuss how host availability, dispersal capacity, and trematode life-history traits may have shaped the observed recolonization dynamics.

Snail abundance was significantly lower in spring than in summer and autumn, presumably reflecting recruitment, growth, and mortality dynamics of gastropods [68]. In contrast, no seasonal patterns were detected in trematode dynamics, which may partly reflect the overall low number of infections recorded. Notably, trematode communities were absent during the initial sampling period and only established following the recolonization of suitable snail hosts. However, once established, no significant changes in snail or trematode species richness and abundance were observed between the study years. Increases in species richness and abundance post-restoration are commonly reported among free-living organisms [8, 69], and comparable trends have been documented for trematode communities following restoration of coastal habitats, where increases in trematode diversity and prevalence are typically interpreted as indicators of increasing trophic complexity and host availability [70–72]. However, explicit studies of parasite recolonization and succession in newly available habitats remain scarce [73–76], and temporal dynamics may only become apparent over time as host and parasite populations mature. Although snail abundance increased with distance to the river mouth, no longitudinal gradient was observed for either snail richness or trematode dynamics, indicating that recolonization and early succession of trematodes did not follow an upstream–downstream pattern along the stream. In contrast to streams characterized by upstream–downstream gradients [77], these findings suggest that recolonization in the Emscher stream is not governed by unidirectional dispersal but instead reflects multiple recolonization sources in the catchment, including the reconnection to the Rhine River and several previously restored tributaries.

Similarly, both snail and trematode communities exhibited high spatial and temporal heterogeneity, with β-diversity driven predominantly by species turnover rather than nestedness-resultant dissimilarities. Although turnover is generally considered the dominant component of β-diversity in both free-living and parasitic taxa [78, 79], species turnover in the present study was more pronounced among trematode communities than among their snail hosts (Fig. 4). In addition, similarity among snail communities significantly decreased with increasing stream distance between sites, whereas trematode communities showed no distance decay as they remained highly dissimilar across the catchment. Because multi-host parasites rely on successive transmission among host species present in the ecosystem, trematode community composition reflects not only the distribution of snail hosts but also that of additional hosts involved in the life cycle. As most trematode species identified in the present study were associated with avian definitive hosts, the elevated turnover and patchy occurrence of individual trematode species (see Additional file 2: Fig. S16) likely reflect early successional dynamics in habitat frequentation and establishment of free-living host organisms. However, highly vagile hosts such as birds are typically associated with reduced spatial structuring and reduced distance decay in parasite communities through dispersal among sites [43, 44, 79], and spatial structuring of trematode communities may emerge with continued host establishment and community succession in the system.

Host-mediated dispersal was further reflected in the recolonization of predominantly allogenic trematode taxa and three taxa with so far unknown definitive hosts which cannot be classified as either autogenic or allogenic. This is consistent with the higher assumed colonization potential of allogenic compared to autogenic parasites [17, 19, 80], in which avian definitive hosts in particular have been identified as key drivers of dispersal and homogenization of trematode communities across spatial scales [81–83]. However, quick recovery following disturbance or restoration has likewise been documented for autogenic trematodes [71], suggesting that as with free-living organisms, recolonization patterns reflect not only dispersal capacity but also the composition of the surrounding species pool [69, 84, 85]. Previous research on trematodes in western Germany indicates that the majority of identified taxa are indeed known from the regional species pool [30, 34, 86–88], with all but one taxon recently documented from the Boye catchment, a restored tributary of the Emscher River [54]. Importantly, trematode communities in these surrounding water bodies are likewise dominated by allogenic taxa [30, 54, 86–88], and consequently, it remains unclear whether recolonization by allogenic trematodes was primarily driven by the high dispersal capacity of definitive hosts, the composition of the species pool, or a combination of both. Nevertheless, the reconnected Rhine River represents a hotspot for both native and introduced parasites [30, 89], and several autogenic trematode taxa have been recorded from snail and fish hosts in the system [30, 90, 91]. Continued documentation of succession in the Emscher River will therefore be necessary to assess temporal and spatial dynamics in recolonization and succession of autogenic and allogenic taxa over time.

Colonization of new habitats is often considered more likely for parasites with direct life cycles, as their establishment does not depend on the presence of multiple successive hosts [23, 81, 92]. In trematodes, life cycle complexity ranges from one to four hosts, with most species exhibiting two- or three-host life cycles [29]. Accordingly, trematode taxa with facultative or obligate life cycle truncation may be more successful in recolonization of restored habitats, as they rely on fewer host species for life cycle completion. In the present study, trematodes with truncated life cycles represented only four of the 16 detected species but accounted for approximately half of the overall prevalence (Fig. 6B), largely due to the high prevalence of *Cephalogonimus* sp. (Table 1). While such disproportionate contributions in prevalence may indicate a higher input of infective stages into the system and potentially reflect successful transmission within suitable host populations in the habitats, the present data do not allow drawing conclusions on establishment or life cycle completion of trematode taxa, as they only indicate the presence of definitive (i.e., upstream) hosts contributing infective stages into the system. In addition, trematode life cycles range from short but specialized to multi-host but generalist strategies that each face distinct challenges for recolonization and establishment [23], highlighting the need to consider trematode life cycle complexity in combination with further life-history traits when investigating recolonization dynamics.

Trematodes typically exhibit higher host specificity toward first intermediate hosts than toward second intermediate and definitive hosts, and molecularly confirmed phylogenetic host generalists among first intermediate snail hosts remain rare [93]. This was reflected in the high phylogenetic host specificity observed for most trematode taxa in this study, contradicting our hypothesis on higher recolonization potential in snail-host generalist trematodes. Across both regional and global scales, the identified trematode taxa were predominantly reported from one or several closely related snail species, typically within the same genus. However, these patterns can only reflect the current state of available data and may change as additional records become available. For instance, several taxa represent undescribed species that have so far been reported only from a small number of localities and hosts [59, 60, 66, 94]. Additional records will thus be required to more reliably assess host specificity in these taxa. Nevertheless, a small number of the identified trematode species were detected in multiple snail genera or families, particularly under consideration of global host records. For instance, *L. linstowi* is predominantly reported from the bithyniid snail *B. tentaculata* [34, 54, 95], but was initially documented from lymnaeid snail *A. balthica* [96]. Likewise, *E. recurvatum* has been documented in its primary lymnaeid snail hosts but also a planorbid snail host in the Boye catchment [54]. Such deviations from primary or preferred first intermediate host species have been reported for several trematode taxa and typically occur in single hosts or small subsets of hosts (e.g., [28, 96]). As these associations represent molecularly verified records, they are unlikely to result from morphological misidentifications and may instead reflect a degree of plasticity in first intermediate host use among these taxa. Further, several trematode taxa documented in this study were identified as geographic host generalists, exploiting different host species within their (narrow) phylogenetic host range across localities. The ability to infect different but closely related host species across locations may therefore be more important for successful recolonization of habitats than phylogenetic host specificity alone. Together, these results highlight the importance of applying multiple measures of host specificity, as well as incorporating host records beyond the scope of a single study [56].

## Conclusions

Due to their complex life cycles, trematodes exhibit distinct life-history traits that may facilitate or constrain recolonization and establishment success. However, the role of these traits in shaping recolonization dynamics has rarely been assessed. Our results indicate that trematode recolonization in the Emscher River was delayed relative to that of snail hosts and characterized by high spatial and temporal heterogeneity. Recolonization was primarily facilitated by mobile definitive hosts from the regional species pool, rather than by phylogenetic host specificity or life cycle complexity of trematodes. The exclusive occurrence of allogenic taxa suggests a key role of high dispersal capacity and flexible habitat use of definitive hosts during early phases of recovery. However, our findings further demonstrate that trematode recolonization cannot be attributed to individual life-history traits in isolation. For example, complex life cycles may facilitate recolonization when combined with geographic host plasticity or highly dispersive hosts, whereas simple life cycles may fail to establish in the absence of suitable hosts. Geographic host turnover within the observed phylogenetic ranges, the large contribution of taxa with truncated life cycles to the overall prevalence, and the strong variation in trematode communities during the early phase of recovery underscore the complex interactions shaping host–parasite dynamics in restored ecosystems. Interpretation of the observed patterns was limited by incomplete knowledge of the host associations and life-history strategies of several detected taxa, as well as the overall low number of infections. Continued monitoring of the Emscher River will be essential to evaluate how life-history traits may influence trematode recolonization and succession as the restored habitats mature.

## Supplementary Information

Additional file 1: **Table S1.** Coordinates of sampling sites and their respective distance from the river mouth. **Table S2.** Primers and PCR thermocycling conditions used in this study. **Table S3.** Summary of all sequences of trematodes used for 28S, ITS1–5.8S–ITS2, *cox*1, and *nad*1 phylogenetic analyses. **Table S4.** Trematode sequence alignments generated in this study.** Figs. S1–S11.** Maximum likelihood phylograms based on the alignments generated in this study.

Additional file 2: **Tables S5–S11.** Summary of GLMM results for all snails and for *A. balthica*. **Figs. S12–S13.** Host richness and abundance and trematode richness and overall prevalence in relation to site distance from the river mouth for all snails and for *A. balthica*. **Fig. S14.** Temporal changes in community composition of trematodes in *A. balthica*. **Fig. S15.** Distance decay of trematode communities in *A. balthica* along the stream. **Fig. S16.** Prevalence of trematode species in the respective snail species identified in this study. **Table S12.** Trematode-host associations included in analyses on host-specificity in this study. **Fig. S17.** Maximum likelihood phylogram based on the *cox*1 mtDNA alignment for gastropod hosts included in analyses on host-specificity. **Tables S13–S14**. Regional and global phylogenetic host specificity and geographic host specificity of trematodes, with resulting classifications.

## Data Availability

All data generated or analyzed during the current study are included in this published article or the Supplementary information. Subsets of this data have already been published and were referenced accordingly [54, 58].
