# Supplementary material for: Trematode recolonization in first intermediate snail hosts after large-scale stream restoration
Source: Parasit Vectors. 2026 Jul 28;19:306. doi: 10.1186/s13071-026-07583-y (PMC13418816; doi:10.1186/s13071-026-07583-y)
Supplement: Supplementary file 1 — Additional file 1: Table S1. Coordinates of sampling sites and their respective distance from the river mouth. Table S2. Primers and PCR thermocycling conditions used in this study. Table S3. Summary of all sequences of trematodes used for 28S, ITS1–5.8S–ITS2, cox1, and nad1 phylogenetic analyses. Table S4. Trematode sequence alignments generated in this study. Figs. S1–S11. Maximum likelihood phylograms based on the alignments generated in this study. [file 13071_2026_7583_MOESM1_ESM.docx]

**Additional file 1.**

**Table S1.** Coordinates of sampling sites and their respective distance from the river mouth.

**Table S2.** Primers and PCR thermocycling conditions used in this study.

**Table S3.** Summary of all sequences of trematodes used for 28S, ITS1–5.8S–ITS2, *cox*1, and *nad*1 phylogenetic analyses.

**Table S4.** Trematode sequence alignments generated in this study.

**Figs. S1–S11.** Maximum likelihood (ML) phylograms based on the alignments generated in this study.

**Table S1.** Coordinates of sampling sites and their respective distance from the river mouth.

| **Sampling site** | **Lat** | **Long** | **Distance from mouth (km)** |
| --- | --- | --- | --- |
| Em05b | 51.5331702 | 7.4234641 | 62.2 |
| Em06 | 51.5487069 | 7.4199889 | 60.6 |
| Em08 | 51.5899948 | 7.3482609 | 53.1 |
| Em08c | 51.5915378 | 7.2883476 | 48.5 |
| Em09 | 51.5756885 | 7.2502334 | 45.0 |
| Em10 | 51.5593037 | 7.2031689 | 40.9 |
| Em11a | 51.5512843 | 7.1393953 | 35.1 |
| Em11 | 51.5484877 | 7.1064348 | 33.9 |
| Em12a | 51.5153838 | 7.0123644 | 26.4 |
| Em13 | 51.5084615 | 6.9763738 | 23.7 |
| Em13c | 51.5036866 | 6.9384139 | 20.9 |
| Em14 | 51.5009370 | 6.8393883 | 13.6 |
| Em16a | 51.5282011 | 6.7946978 | 8.8 |
| Em17 | 51.5570267 | 6.7201967 | 2.6 |
| Em18 | 51.5603808 | 6.7038636 | 0.5 |

**Table S2.** Primers and PCR thermocycling conditions used in this study. The PCR protocol for all samples featured a 20 µL reaction volume with 10 µL DreamTaq^TM^ Hot Start Green PCR Master Mix, 1.6 µL of each primer (10 µM), 4.8 µL molecular-grade water, and 2 µL DNA.

| **Gene region** | **Primer pair** | **Nucleotide sequence (5’–3’)** | **Product size (base pair, bp)** | **Cycling parameters** | **Reference** |
| --- | --- | --- | --- | --- | --- |
| **Snails** | | | | | |
| *cox*1 | LCO1490 | GGT CAA CAA ATC ATA AAG ATA TTG G | ~658 bp | 95 °C-3 min,  [96 °C-30 s, 45 °C-25 s, 72 °C-55 s] x 10,  [98 °C-30 s, 54 °C-25 s, 72 °C, 55 s] x 35,  72 °C-5 min | Folmer et al. (1994) |
|  | HCO2198 | TAA ACT TCA GGG TGA CCA AAA AAT CA |  |  |  |
| **Trematodes** | | | | | |
| 28S | digl2 | AAG CAT ATC ACT AAG CGG | ~1200 bp | 94 °C-4 min,  [94 °C-30 s, 50-56 °C-30 s, 72 °C-2 min] x 40,  72 °C-5 min | Tkach et al. (2001a) |
|  | 1500R | GCT ATC CTG AGG GAA ACT TCG |  |  | Snyder and Tkach (2001) |
|  | 300F* | CAA GTA CCG TGA GGG AAA GTT G |  |  | Littlewood et al. (2000) |
|  | ECD2* | CCT TGG TCC GTG TTT CAA GAC GGG |  |  | Littlewood et al. (1997) |
| ITS1–5.8S–ITS2 | D1 | AGG AAT TCC TGG TAA GTG CAA G | ~1200 bp | 94 °C-2 min,  [94 °C-1 min, 56 °C-1 min, 72 °C-2 min] x 30,  72 °C-5 min | Galazzo et al. (2002) |
|  | D2 | CGT TAC TGA GGG AAT CCT GGT |  |  |  |
| *cox*1 | JB3 | TTT TTT GGG CAT CCT GAG GTT TAT | ~400 bp | 95 °C -5 min,  [94 °C-50 s, 53 °C-50 s, 72 °C-50 s] x 35,  72 °C-10 min | Bowles et al. (1995) |
|  | JB4.5 | TAA AGA AAG AAC ATA ATG AAA ATG |  |  |  |
| *nad*1 | NDJ11 | AGA TTC GTA AGG GGC CTA ATA | ~500 bp | 95 °C -5 min,  [94 °C-30 s, 48 °C-30 s, 72 °C-45 s] x 35,  72 °C-7 min | Kostadinova et al. (2003) |
|  | NDJ2a | CTT CAG CCT CAG CAT AAT |  |  |  |

^*^Sequencing primer.

**Table S3.** Summary of all sequences of trematodes used for 28S, ITS1–5.8S–ITS2, *cox*1, and *nad*1 phylogenetic analyses. Novel sequences generated in this study are highlighted in bold. Life cycle stage: A, adult; P, parthenitae; C, cercaria; M, metacercaria.

| **Taxon** | **Life cycle stage** | **Host** | **Country** | **GenBank Accession No.** | | **Reference** |
| --- | --- | --- | --- | --- | --- | --- |
|  |  |  |  | **28S/ ITS1–5.8S–ITS2^a^** | ***cox*1/ *nad*1^b^** |  |
| **Family Cephalogonimidae** | | | | | | |
| *Cephalogonimus americanus* | A | *Ambystoma velasci* | Mexico | HM137615 | – | Razo-Mendivil & Pérez-Ponce de León (2011) |
| *Cephalogonimus americanus* | A | *Lithobates pipiens* | USA | PQ013252 | – | Johnson et al. (2024) |
| *Cephalogonimus americanus* | C | *Planorbella trivolvis* | USA | PQ013254 | – | Johnson et al. (2024) |
| *Cephalogonimus retusus* | A | *Pelophylax ridibundus* | Bulgaria | AY222276 | – | Olson et al. (2003) |
| *Cephalogonimus* sp. | A | *Lithobathes* sp. | Mexico | MK648268 | – | Pérez-Ponce de León & Hernández-Mena (2019) |
| *Cephalogonimus* sp. | C | *Ampullaceana balthica* | Germany | PV248747 | – | Hüsken et al. (2025) |
| *Cephalogonimus* sp. | C | *Ampullaceana balthica* | Germany | PX641077 | PX637233 | Hüsken et al. (2026) |
| *Cephalogonimus* sp. | C | *Ampullaceana balthica* | Germany | PX641080 | – | Hüsken et al. (2026) |
| ***Cephalogonimus* sp.** | **C** | ***Ampullaceana balthica*** | **Germany** | **PX972818** | – | **This study** |
| ***Cephalogonimus* sp.** | **C** | ***Ampullaceana balthica*** | **Germany** | **PX972819** | – | **This study** |
| *Masenia nkomatiensis* | A | *Clarias gariepinus* | Mozambique | MH142268 | – | Dumbo et al. (2019) |
| **Family Clinostomidae** | | | | | | |
| *Clinostomum complanatum* | M | *Heteropneustes fossilis* | India | KF811011 | – | Tandon et al. (2013) |
| **Family Cyathocotylidae** | | | | | | |
| *Cyathocotyle bushiensis* | A | *Aythya affinis* | USA | MK650440 | – | Achatz et al. (2019) |
| **Family Diplostomidae** | | | | | | |
| *Alaria alata* | A | *Sus scrofa* | Poland | – | MT103225 | Bilska-Zając et al. (2021) |
| *Alaria americana* | A | – | Canada | – | MZ605229 | Bouchard et al. (2021) |
| *Hysteromorpha triloba* | – | *Ameiurus melas* | USA | HM114365 | – | Tkach et al. (2010) |
| *Tylodelphys clavata* | M | *Coregonus lavaretus* | Germany | JQ665459^a^ | – | Behrmann-Godel (2013) |
| *Tylodelphys scheuringi* | M | *Ambloplites rupestris* | Canada | FJ469596^a^ | – | Moszczynska et al. (2009) |
| **Family Echinochasmidae** | | | | | | |
| *Echinochasmus japonicus* | A | *Homo sapiens* | Vietnam | OR532444 | – | Le et al. (2024) |
| **Family Echinostomatidae** | | | | | | |
| *Ehinoparyphium ellisi* | A | *Anas platyrhynchos* | New Zealand | – | KY436405^b^ | Georgieva et al. (2017) |
| *Echinoparyphium recurvatum* | C | *Lymnaea peregra* | UK | – | AY168944^b^ | Kostadinova et al. (2003) |
| *Echinoparyphium recurvatum* | C | *Ampullaceana balthica* | Iceland | MZ409804 | – | Pantoja et al. (2021) |
| *Echinoparyphium recurvatum* | M | *Lithobates sylvaticus* | USA | JF820594 | – | Pulis et al. (2011) |
| *Echinoparyphium recurvatum* | C | *Radix ovata* | Slovakia | KT956913 | – | Tkach et al. (2016) |
| *Echinoparyphium recurvatum* | M | *Sphaerium* sp. | Norway | – | KY513267^b^ | Georgieva et al. (2013) |
| *Echinoparyphium recurvatum* | C | *Planorbis carinatus* | Germany | – | PX648502^b^ | Hüsken et al. (2026) |
| *Echinoparyphium recurvatum* | C | *Ampullaceana balthica* | Iceland | – | MZ404649^b^ | Pantoja et al. (2021) |
| *Echinoparyphium recurvatum* | C | *Stagnicola palustris* | Germany | PX641046 | – | Hüsken et al. (2026) |
| ***Echinoparyphium recurvatum*** | **C** | ***Ampullaceana balthica*** | **Germany** | **PX972823** | **PX993637^b^–PX993638^b^, PX993641^b^** | **This study** |
| *Echinoparyphium rubrum* | C | *Stagnicola elodes* | USA | – | MZ404659^b^ | Pantoja et al. (2021) |
| *Echinoparyphium* sp. | A | *Anas platyrhynchos* dom. | Thailand | – | LC599760**^b^** | Le et al. (2024) |
| *Echinoparyphium* sp. | C | *Ladislavella elodes* | Canada | – | MH368972^b^ | Gordy & Hanington (2019) |
| *Echinoparyphium* sp. | C | *Physella ancilaria* | USA | – | PQ381493^b^ | Schumacher & Minchella (2026) |
| *Echinostoma cinetorchis* | A | *Gallus gallus* dom. | Russia | – | MT592854^b^ | Izrailskaia et al. (2021) |
| *Echinostoma miyagawai* | A | *Anas platyrhynchos* dom. | Laos | – | KP455625^b^ | Nagataki et al. (2015) |
| *Echinostoma miyagawai* | A | *Anas platyrhynchos* dom. | Thailand | OR509027 | – | Le et al. (2024) |
| *Echinostoma novaezealandense* | A | *Anas platyrhynchos* | New Zealand | – | KY436399^b^ | Georgieva et al. (2017) |
| *Echinostoma paraulum* | C | *Lymnaea stagnalis* | Germany | KP065604 | – | Georgieva et al. (2014) |
| *Echinostoma revolutum* (s.s.) | C | *Lymnaea stagnalis* | Czech Republic | KP065594 | – | Georgieva et al. (2014) |
| *Echinostoma revolutum* (s.s.) | C | *Ampullaceana balthica* | Iceland | MZ409810 | – | Pantoja et al. (2021) |
| *Echinostoma revolutum* (s.s.) | C | *Lymnaea stagnalis* | Germany | – | KP065649^b^ | Georgieva et al. (2014) |
| *Echinostoma revolutum* (s.s.) | C | *Ampullaceana balthica* | Iceland | – | MZ404671^b^ | Pantoja et al. (2021) |
| *Echinostoma revolutum* (s.s.) | C | *Ampullaceana balthica* | Germany | PX641037 | PX648495^b^ | Hüsken et al. (2026) |
| *Echinostoma revolutum* (s.s.) | A | *Anas platyrhynchos* dom. | Bangladesh | – | LC224103^b^ | Mohanta et al. (2019) |
| ***Echinostoma revolutum*** (s.s.) | **C** | ***Ampullaceana balthica*** | **Germany** | **PX972830** | **PX993642^b^, PX993644^b^–PX993645^b^** | **This study** |
| *Echinostoma revolutum* (s.l.) | C | *Ladisvella elodes* | Canada | – | MH369201^b^ | Gordy & Hanington (2019) |
| *Echinostoma revolutum* (s.l.) | C | *Radix auricularia* | USA | – | MZ404673^b^ | Pantoja et al. (2021) |
| *Echinostoma revolutum* (s.l.) | A | *Anas gracilis* | Australia | – | OR257454^b^ | Ray et al. (2024) |
| *Echinostoma revolutum* (s.l.) | C | *Lymnaea elodes* | USA | – | GQ463082^b^ | Detwiler et al. (2010) |
| *Echinostoma robustum* | – | – | – | – | PQ421443^b^ | Schumacher & Minchella (2026) |
| *Drepanocephalus auratus* | C | *Biomphalaria havanensis* | USA | KY677977^a^ | – | Alberson et al. (2017) |
| *Hypoderaeum conoideum* | P | *Lymnaea stagnalis* | Poland | OQ672266 | – | Kanarek et al. (2023) |
| *Hypoderaeum conoideum* | A | *Anas platyrhynchos* | Ukraine | KT956918 | – | Tkach et al. (2016) |
| *Hypoderaeum conoideum* | C | *Lymnaea stagnalis* | Czech Republic | KP065607 | – | Georgieva et al. (2014) |
| *Hypoderaeum conoideum* | C | *Ampullaceana balthica* | Germany | PX641054 | – | Hüsken et al. (2026) |
| ***Hypoderaeum conoideum*** | **C** | ***Ampullaceana balthica*** | **Germany** | **PX972835** | **PX993646^b^– PX993648^b^** | **This study** |
| *Hypoderaeum conoideum* | C | *Lymnaea stagnalis* | Finland | – | MZ404681^b^ | Pantoja et al. (2021) |
| *Hypoderaeum conoideum* | C | *Lymnaea peregra* | Bulgaria | – | AY168949**^b^** | Kostadinova et al. (2003) |
| *Hypoderaeum conoideum* | C | *Radix auricularia* | UK | – | ON653297^b^ | Enabulele et al. (2023) |
| *Hypoderaeum* sp. | C | *Lymnaea elodes* | USA | – | GQ463097^b^ | Detwiler et al. (2010) |
| *Petasiger phalacrocoracis* | A | *Phalacrocorax carbo* | Ukraine | KT956926 | – | Tkach et al. (2016) |
| *Petasiger phalacrocoracis* | M | *Tinca tinca* | Lithuania | PX614069 | – | Kudlai et al. (2026) |
| *Petasiger phalacrocoracis* | M | *Rutilus rutilus* | Hungary | KY284004 | – | Cech et al. (2017) |
| *Petasiger phalacrocoracis* | M | *Scardinius erythrophthalmus* | Hungary | KY284005 | – | Cech et al. (2017) |
| *Petasiger phalacrocoracis* | A | *Phalacrocorax carbo* | Hungary | KY284006 | – | Cech et al. (2017) |
| *Petasiger phalacrocoracis* | A | *Phalacrocorax carbo* | Hungary | KY284008 | – | Cech et al. (2017) |
| *Petasiger phalacrocoracis* | P | *Radix plicatula* | Japan | LC818882 | – | Seo et al. (2024) |
| ***Petasiger phalacrocoracis*** | **C** | ***Ampullaceana balthica*** | **Germany** | **PX972844** | – | **This study** |
| *Petasiger radiatus* | M | *Carassius gibelio* | Lithuania | PX600354 | – | Kudlai et al. (2026) |
| *Petasiger* sp. | C | *Isidorella hainesii* | Australia | OM305031 | – | Barton et al. (2022) |
| *Petasiger* sp. | M | *Rutilus rutilus* | Hungary | KY284003 | – | Cech et al. (2017) |
| *Petasiger phalacrocoracis* | A | *Phalacrocorax carbo* | Israel | AY245709^a^ | – | Dzikowski et al. (2004) |
| *Petasiger phalacrocoracis* | A | *Phalacrocorax carbo* | Hungary | PP188697^a^ | – | Gyöngy et al. (2024) |
| *Petasiger phalacrocoracis* | C | *Ampullaceana balthica* | Denmark | MW001053^a^ | – | Duan et al. (2021) |
| *Petasiger phalacrocoracis* | M | *Rutilus rutilus* | Hungary | KJ720683^a^ | – | Molnar et al. (2015) |
| *Petasiger phalacrocoracis* | C | *Physa fontinalis* | Russia | OR037388^a^ | – | Vainutis et al. (2023) |
| *Petasiger phalacrocoracis* | A | *Phalacrocorax carbo* | Hungary | PP188696^a^ | – | Gyöngy et al. (2024) |
| ***Petasiger phalacrocoracis*** | **C** | ***Ampullaceana balthica*** | **Germany** | **PZ225369^a^, PZ225375^a^–PZ225376^a^** | – | **This study** |
| *Petasiger radiatus* | A | *Phalacrocorax carbo* | Hungary | KM973000^a^ | – | Molnar et al. (2015) |
| *Petasiger radiatus* | A | *Phalacrocorax carbo* | Israel | AY245708^a^ | – | Dzikowski et al. (2004) |
| *Petasiger* sp. | – | – | – | ON866948^a^ | – | Shamsi et al. (2024) |
| *Petasiger* sp. | – | – | – | OM305105^a^ | – | Shamsi et al. (2024) |
| *Petasiger* sp. | M | *Rutilus rutilus* | Hungary | KM972995^a^ | – | Molnar et al. (2015) |
| **Family Fasciolidae** | | | | | | |
| *Fasciola hepatica* | A | *Bos taurus* | Ecuador | – | LC273114^b^ | Amer and Maza (2017) |
| **Family Haematoloechidae** | | | | | | |
| *Haematoloechus longiplexus* | A | *Rana catesbeiana* | USA | AF387801 | – | Snyder and Tkach (2001) |
| **Family Lecithodendriidae** | | | | | | |
| *Lecithodendrium linstowi* | M | *Nyctalus noctula* | Ukraine | AF151919 | – | Tkach et al. (2000) |
| *Lecithodendrium linstowi* | A | *Pipistrellus pipistrellua* | England | JF784191 | – | Lord et al. (2012) |
| *Lecithodendrium linstowi* | C | *Ampullaceana balthica* | UK | MF498821 | – | Enabulele et al. (2018) |
| *Lecithodendrium linstowi* | C | *Bithynia tentaculata* | Ireland | PP849700 | – | Faltýnková et al. (2024) |
| *Lecithodendrium linstowi* | C | *Bithynia tentaculata* | Germany | MN726965 | – | Schwelm et al. (2020) |
| ***Lecithodendrium linstowi*** | **C** | ***Bithynia tentaculata*** | **Germany** | **PX972837** | – | **This study** |
| *Lecithodendrium skrjabini* | A | *Pipistrellus kuhlii* | Russia | MK575196 | – | Sokolov et al. (2020) |
| *Lecithodendrium* sp. | C | *Bithynia tentaculata* | Ireland | PP849702 | – | Faltýnková et al. (2024) |
| *Lecithodendrium* sp. | P | *Bithynia tentaculata* | Lithuania | KJ126726 | – | Kudlai et al. (2015) |
| *Lecithodendrium* sp. | C | *Bithynia siamensis goniomphalos* | Vietnam | ON986398 | – | Nguyen et al. (2022) |
| *Ochoterenatrema fratenum* | A | *Myotis diminutus* | Ecuador | PP534961 | – | Tkach et al. (2024) |
| *Ochoterenatrema gracilis* | A | *Perimyotis subflavus* | USA | OM574910 | – | Fernandes et al. (2022) |
| *Ochoterenatrema giovannionorei* | A | *Molossus molossus* | Ecuador | PP534962 | – | Tkach et al. (2024) |
| *Ochoterenatrema piriforme* | A | *Myotis diminutus* | Ecuador | PP069556 | – | Tkach et al. (2024) |
| *Paralecithodendrium hurkovaae* | M | *Myotis daubentoni* | Ukraine | AF151922 | – | Tkach et al. (2000) |
| *Paralecithodendrium* sp. | C | *Bithynia funiculate* | Vietnam | OM971672 | – | Nguyen et al. (2022) |
| *Pycnoporus heteroporus* | M | *Myotis daubentoni* | Ukraine | AF151918 | – | Tkach et al. (2000) |
| **Family Microphallidae** | | | | | | |
| *Microphallus similis* | A | *Carcinus maenas* | UK | AY220625 | – | Tkach et al. (2003) |
| **Family Notocotylidae** | | | | | | |
| *Notocotylus attenuatus* | A | *Aythya ferina* | Ukraine | AF184259 | – | Tkach et al. (2001b) |
| *Notocotylus atlanticus* | A | *Anas platyrhynchos* | Russia | MH818008 | – | Gonchar et al. (2019) |
| *Notocotylus ikutai* | C | *Radix auricularia* | Japan | LC596925 | – | Sasaki et al. (2021) |
| *Notocotylus intestinalis* | C | *Parafossarulus striatulus* | Vietnam | JQ890559 | – | Besprozvannykh et al. (2013) |
| *Notocotylus triserialis* | A | *Anser albifrons* | Russia | PV658391 | – | Vlasenkov et al. (2025) |
| *Notocotylus* sp. AK-2017 | C | *Ampullaceana balthica* | Norway | KY513158 | – | Soldánová et al. (2017) |
| ***Notocotylus* sp.** | **C** | ***Ampullaceana balthica*** | **Germany** | **PX972840** | – | **This study** |
| *Notocotylus* sp. OK-2019 | C | *Bithynia tentaculata* | Germany | MN726956– MN726956 | – | Schwelm et al. (2020) |
| *Notocotylus* sp. | C | *Bithynia tentaculata* | Ireland | PP849713 | – | Faltýnková et al. (2024) |
| *Notocotylus* sp. | A | *Anas platyrhynchos* | Russia | OP981945– OP981946 | – | Vinogradova et al. (2022) |
| ***Notocotylus* sp.** | **C** | ***Bithynia tentaculata*** | **Germany** | **PX972842** | – | **This study** |
| *Notocotylus* sp. | P | *Radix auricularia* | Japan | LC599519 | – | Nakao & Sasaki (2021) |
| *Notocotylus* sp. | P | *Radix auricularia* | Japan | LC599518 | – | Nakao & Sasaki (2021) |
| *Notocotylus* sp. | P | *Radix auricularia* | Poland | OQ672264 | – | Kanarek et al. (2023) |
| **Family Plagiorchiidae** | | | | | | |
| *Plagiorchis elegans* | C | *Lymnaea stagnalis* | Slovakia | KJ533392 | KJ533402 | Zikmundová et al. (2014) |
| *Plagiorchis elegans* | C | *Lymnaea stagnalis* | Germany | PX641036 | PX637202 | Hüsken et al. (2026) |
| ***Plagiorchis elegans*** | **C** | ***Stagnicola palustris*** | **Germany** | **PX972847** | **PX970448** | **This study** |
| *Plagiorchis elegans* | C | *Lymnaea stagnalis* | Czech Republic | – | PP396753 | Kundid et al. (2024) |
| *Plagiorchis muelleri* | A | *Eptesicus serotinus* | Ukraine | AF184250 | – | Tkach et al. (2001b) |
| *Plagiorchis vespertilionis* | A | *Myotis daubentoni* | Ukraine | AF151931 | – | Tkach et al. (2000) |
| *Plagiorchis vespertilionis* | C | *Radix auricularia* | Czech Republic | – | PP396758 | Kundid et al. (2024) |
| *Plagiorchis vespertilionis* | C | *Ampullaceana balthica* | Germany | PX641068 | PX637226 | Hüsken et al. (2026) |
| ***Plagiorchis vespertilionis*** | **C** | ***Ampullaceana balthica*** | **Germany** | **PX972853** | **PX970454** | **This study** |
| *Plagiorchis* sp. 2 AK-2017 | C | *Ampullaceana balthica* | Norway | KY513164 | – | Soldánová et al. (2017) |
| *Plagiorchis* sp. 2 AK-2017 | M | *Gammarus lacustris* | Norway | – | KY513254 | Soldánová et al. (2017) |
| *Plagiorchis* sp. 2 OK-2021 | C | *Ampullaceana balthica* | Iceland | – | MW520071 | Kudlai et al. (2021) |
| *Plagiorchis* sp. 2 | C | *Ampullaceana balthica* | Germany | PX641049 | PX637213 | Hüsken et al. (2026) |
| ***Plagiorchis* sp. 2** | **C** | ***Ampullaceana balthica*** | **Germany** | **PX972848** | **PX970449** | **This study** |
| *Plagiorchis* sp. 3 AK-2017 | C | *Ampullaceana balthica* | Norway | KY513166 | KY513257 | Soldánová et al. (2017) |
| *Plagiorchis* sp. 3 | C | *Ampullaceana balthica* | Iceland | – | MW520079 | Kudlai et al. (2021) |
| *Plagiorchis* sp. 3 | C | *Ampullaceana balthica* | Germany | PX641041 | PX637207 | Hüsken et al. (2026) |
| ***Plagiorchis* sp. 3** | **C** | ***Ampullaceana balthica*** | **Germany** | **PX972849** | **PX970450** | **This study** |
| *Plagiorchis* sp. 3 | C | *Ampullaceana balthica* | Czech Republic | – | PP396791 | Kundid et al. (2024) |
| *Plagiorchis* sp. 4 AK-2017 | C | *Ampullaceana balthica* | Norway | KY513169 | – | Soldánová et al. (2017) |
| *Plagiorchis* sp. 5 AK-2017 | M | *Oreodytes alpinus* | Norway | KY513170 | – | Soldánová et al. (2017) |
| *Plagiorchis* sp. 6 OK-2021 | C | *Ampullaceana balthica* | Ireland | – | MW520082 | Kudlai et al. (2021) |
| *Plagiorchis* sp. 7 AK-2017 | C | *Ampullaceana balthica* | Norway | KY513174 | KY513264 | Soldánová et al. (2017) |
| *Plagiorchis* sp. 7 OK-2021 | C | *Ampullaceana balthica* | Ireland | – | MW520083 | Kudlai et al. (2021) |
| *Plagiorchis* sp. 7 | C | *Ampullaceana balthica* | Germany | PX641050 | PX637214 | Hüsken et al. (2026) |
| ***Plagiorchis* sp. 7** | **C** | ***Ampullaceana balthica*** | **Germany** | **PX972850** | **PX970451** | **This study** |
| *Plagiorchis* sp. 8 OK-2021 | C | *Ampullaceana balthica* | Ireland | MW528619 | MW520084 | Kudlai et al. (2021) |
| *Plagiorchis* sp. 9 OK-2021 | C | *Stagnicola fuscus* | Ireland | MW528621 | – | Kudlai et al. (2021) |
| *Plagiorchis* sp. 11 | C | *Ampullaceana balthica* | Czech Republic | – | PP407075 | Kundid et al. (2024) |
| **Pronocephalidae** | | | | | | |
| ‘Pronocephaloidea sp.’ | C | *Potamopyrgus antipodarum* | USA | EU371602 | – | Adema et al. (2009) |
| **Family Strigeidae** | | | | | | |
| *Apatemon gracilis* | C | *Ampullaceana balthica* | Norway | KY513175 | – | Soldánová et al. (2017) |
| *Apatemon* sp. | M | *Gastrosteus aculeatus* | Norway | KY513178 | – | Soldánová et al. (2017) |
| *Apatemon* sp. 5 | C | *Planorbis planorbis* | Lithuania | OQ102386^a^ | – | Faltýnková et al. (2023) |
| *Apatemon* sp. 6 | C | *Gyraulus parvus* | Iceland | OQ102389^a^ | – | Faltýnková et al. (2023) |
| *Apharyngostrigea cornu* | A | *Ardea herodias* | Mexico | PX620571^a^ | – | López-Jiménez et al. (2025) |
| *Apharyngostrigea pipientis* | A | *Ardea alba* | Mexico | PX620580^a^ | – | López-Jiménez et al. (2025) |
| *Australapatemon burti* | A | *Anas diazi* | Mexico | MF398342 | – | Hernández-Mena et al. (2017) |
| *Australapatemon burti* | C | *Helisoma trivolvis* | Canada | KY207626^a^ | – | Gordy et al. (2017) |
| *Australapatemon burti* | C | *Planorbis planorbis* | Denmark | MW000965^a^ | – | Duan et al. (2021) |
| *Australapatemon burti* | A | *Anas diazi* | Mexico | JX977787^a^ | – | Hernández-Mena et al. (2014) |
| *Australapatemon burti* | A | *Oxyura jamaicensis* | Mexico | JX977788^a^ | – | Hernández-Mena et al. (2014) |
| *Australapatemon* cf. *burti* | C | *Planorbella trivolvis* | USA | PQ013264 | – | Johnson et al. (2024) |
| *Australapatemon fuligulae* | A | *Aythya ferina* | Poland | MW244656^a^ | **–** | Pyrka et al. (2021) |
| *Australapatemon mclaughlini* | C | *Physella gyrina* | Canada | KY207628^a^ | – | Gordy et al. (2017) |
| *Australapatemon niewiadomski* | A | *Anas platyrhynchos* | New Zealand | KT334165, KT334175^a^ | – | Blasco-Costa et al. (2016) |
| *Australapatemon* sp. | C | *Planorbella sp.* | USA | OK284407 | – | Keller et al. (2021) |
| ***Australapatemon* sp.** | **C** | ***Ampullaceana balthica*** | **Germany** | **PX972814 /  PZ225372^a^, PZ225374^a^** | **–** | **This study** |
| *Australapatemon* sp. | C | *Ampullaceana balthica* | Denkmark | MW000967^a^ | **–** | Duan et al. (2021) |
| *Australapatemon* sp. | C | *Stagnicola palustris* | Denmark | MW000966^a^ | **–** | Duan et al. (2021) |
| *Australapatemon* sp. | A | *Oxyura jamaicensis* | Canada | KY570946^a^ | **–** | Gordy et al. (2017) |
| *Australapatemon* sp. | C | Planorbidae sp. | Canada | KY570947^a^ | **–** | Gordy et al. (2017) |
| *Australapatemon* sp. | C | Planorbidae sp. | Canada | KY570948^a^ | **–** | Gordy et al. (2017) |
| *Australapatemon* sp. | M | *Haemopis sanguisuga* | Poland | MW244650^a^ | **–** | Pyrka et al. (2021) |
| *Australapatemon* sp. | C | *Ampullaceana balthica* | Denmark | MW001126^a^ | **–** | Duan et al. (2021) |
| *Cotylurus brandivitellata* | A | *Cygnus olor* | Poland | **–** | PX056574 | Gabrysiak et al. (2025) |
| *Cotylurus cornutus* | M | *Stagnicola palustris* | Lithuania | PX457623 | – | Stunzenas (2025) |
| *Cotylurus cornutus* | A | *Anas platyrhynchos* | Poland | – | MW204806 | Pyrka et al. (2021) |
| *Cotylurus cornutus* | A | *Anas platyrhynchos* | Poland | – | OM949020–OM949021 | Pyrka et al.(2022) |
| *Cotylurus marcogliesei* | A | *Lophodytes cucullatus* | Canada | MH521248 | MH536509 | Locke et al. (2018) |
| *Cotylurus strigeoides* | C | *Physa fontinalis* | Lithuania | PX457627 | – | Stunzenas (2025) |
| *Cotylurus strigeoides* | A | *Anas platyrhynchos* | Poland | – | MW204807 | Pyrka et al. (2021) |
| *Cotylurus strigeoides* | A | *Anas platyrhynchos* | Poland | – | OM949019 | Pyrka et al.(2022) |
| *Cotylurus strigeoides* | C | *Physella gyrina* | Canada | – | PX252461 | McPhail et al. (2026) |
| *Cotylurus syrius* | A | *Cygnus olor* | Poland | MW244648 | MW204819 | Pyrka et al. (2021) |
| *Cotylurus syrius* | A | *Cygnus olor* | Czech Republic | – | MF628056, MF628058 | Heneberg et al. (2018) |
| *Cotylurus* sp. (lineage II) | M | *Radix auricularia* | Poland | OM949867, OM949869 | OM949022–OM949025 | Pyrka et al. (2022) |
| *Cotylurus* sp. (lineage III) | M | *Planorbarius corneus* | Poland | – | OM949031 | Pyrka et al. (2022) |
| *Cotylurus* sp. (lineage V) | M | *Planorbis planorbis* | Poland | – | OM949040 | Pyrka et al. (2022) |
| ***Cotylurus* sp.** | **C** | ***Ampullaceana balthica*** | **Germany** | **PX972821** | **PX970444–PX970446** | **This study** |
| *Cotylurus* sp. | P | *Radix auricularia* | Japan | – | LC599701 | Nakao & Sasaki (2021) |
| *Cotylurus* sp. | P | *Radix auricularia* | Japan | – | LC599706, LC599707, LC599710 | Nakao & Sasaki (2021) |
| *Cotylurus* sp. | C | *Lymnaea stagnalis* | Canada | – | PV601174 | McPhail et al. (2025) |
| *Cotylurus* sp. | M | *Radix auricularia* | Japan | – | LC599685 | Nakao & Sasaki (2021) |
| *Cotylurus* sp. | M | *Radix auricularia* | Japan | – | LC599696 | Nakao & Sasaki (2021) |
| *Cotylurus* sp. | C | *Ampullaceana balthica* | Germany | – | PX637219 | Hüsken et al. (2026) |
| **Family Telorchiidae** | | | | | | |
| *Opisthiogylphe ranae* | A | *Rana arvalis* | Ukraine | AF151929 | – | Tkach et al. (2000) |
| *Opisthiogylphe ranae* | M | *Pelophylax ridibundus* | Russia | MK585340 | – | Svinin et al. (2023) |
| *Opisthiogylphe ranae* | M | *Pelophylax ridibundus* | Russia | MK585341 | – | Svinin et al. (2023) |
| *Opisthiogylphe ranae* | C | *Stagnicola palustris* | Germany | PX641055 | – | Hüsken et al. (2026) |
| ***Opisthioglyphe ranae*** | **C** | ***Ampullaceana balthica*** | **Germany** | **PX972843** | – | **This study** |
| *Opisthioglyphe* sp. | C | *Tibetoradix hookeri* | China | ON792555 | – | Khrebtova & Kondakov (2022) |
| *Opisthioglyphe* sp. | C | *Tibetoradix hookeri* | China | ON792554 | – | Khrebtova & Kondakov (2022) |
| *Opisthioglyphe* sp. | C | *Ampullaceana balthica* | Spain | ON792556 | – | Khrebtova & Kondakov (2022) |
| *Telorchis assula* | C | *Ampullaceana fontinalis* | Russia | ON792557 | – | Khrebtova & Kondakov (2022) |
| *Telorchis bonnerensis* | A | *Chelydra serpentine* | USA | JF820592 | – | Pulis et al. (2011) |
| *Telorchis corti* | – | *Radix coreana* | South Korea | ON792561 | – | Khrebtova & Kondakov (2022) |

^a^ ITS1–5.8S–ITS2 sequences

^b^ *nad*1 sequences

| **Trematode family** | **Gene region** | **No. of sequences** | **Alignment length (bp)** | **Model ML** | **Figure** |
| --- | --- | --- | --- | --- | --- |
| Cephalogonimidae, Telorchiidae | 28S | 23 | 1,108 | TVM+F+G4 | S1 |
| Echinostomatidae | 28S | 28 | 1,006 | TIM3+F+I | S2 |
|  | nad1 | 35 | 437 | K3Pu+F+I+R3 | S3 |
|  | ITS1–5.8S–ITS2 | 15 | 973 | TVM+F+G4 | S4 |
| Lecithodendriidae | 28S | 18 | 815 | TIM2+F+G4 | S5 |
| Notocotylidae | 28S | 19 | 1,259 | TVM+F+I | S6 |
| Plagiorchiidae | 28S | 21 | 1,117 | TVM+F+R3 | S7 |
|  | cox1 | 24 | 315 | TN+F+I+R2 | S8 |
| Strigeidae | 28S | 15 | 1,053 | TVM+F+I | S9 |
|  | cox1 | 30 | 283 | TN+F+I+R2 | S10 |
|  | ITS1–5.8S–ITS2 | 22 | 884 | TPM2u+F+G4 | S11 |

**Table S4.** Trematode sequence alignments generated in this study.

**
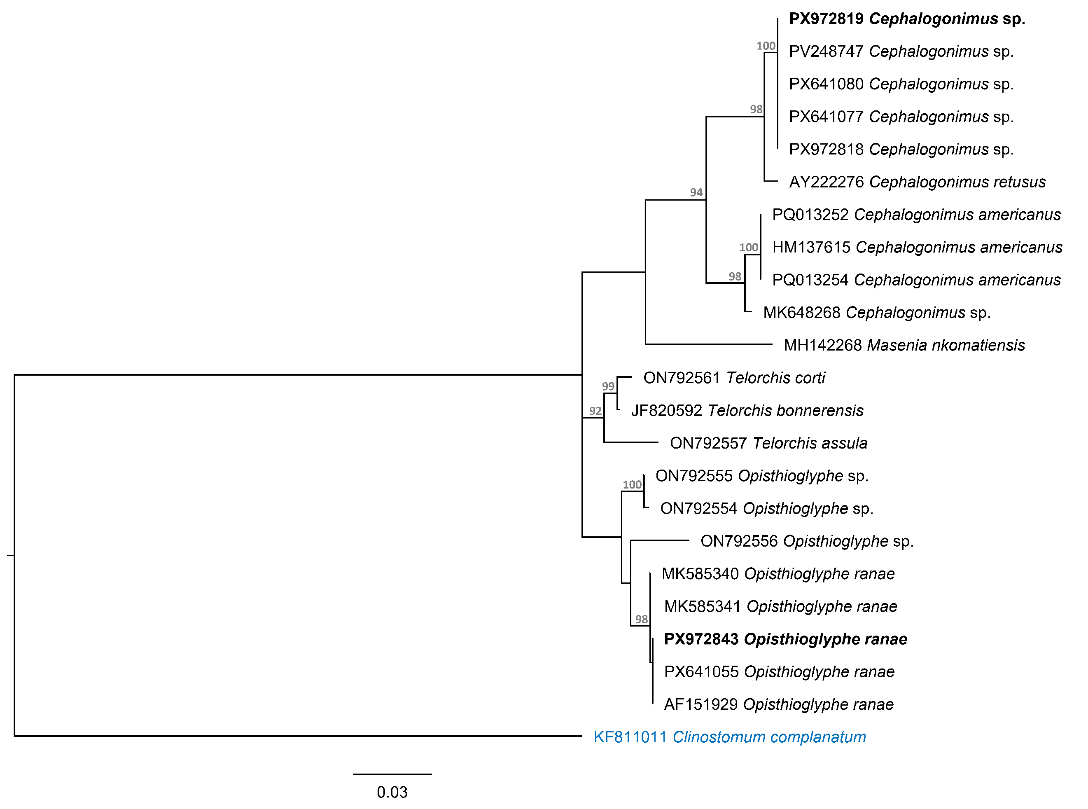

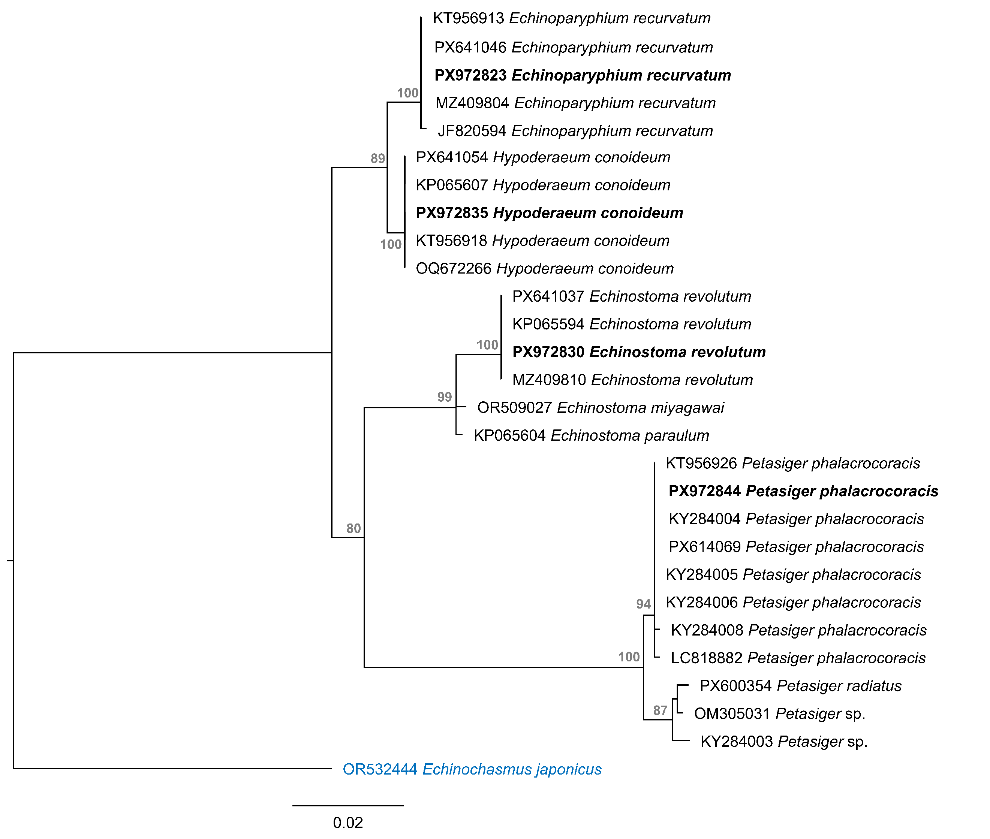
Fig. S1.** Maximum likelihood (ML) phylogram based on the 28S rDNA alignment of Cephalogonimidae and Telorchiidae. Novel sequences are highlighted in bold. Node support values >75 (1000 bootstrap replicates) are shown in grey. Scale bar indicates the expected number of substitutions per site, the outgroup is indicated in blue.

**Fig. S2.** Maximum likelihood (ML) phylogram based on the 28S rDNA alignment of Echinostomatidae. Novel sequences are highlighted in bold. Node support values >75 (1000 bootstrap replicates) are shown in grey. Scale bar indicates the expected number of substitutions per site, the outgroup is indicated in blue.


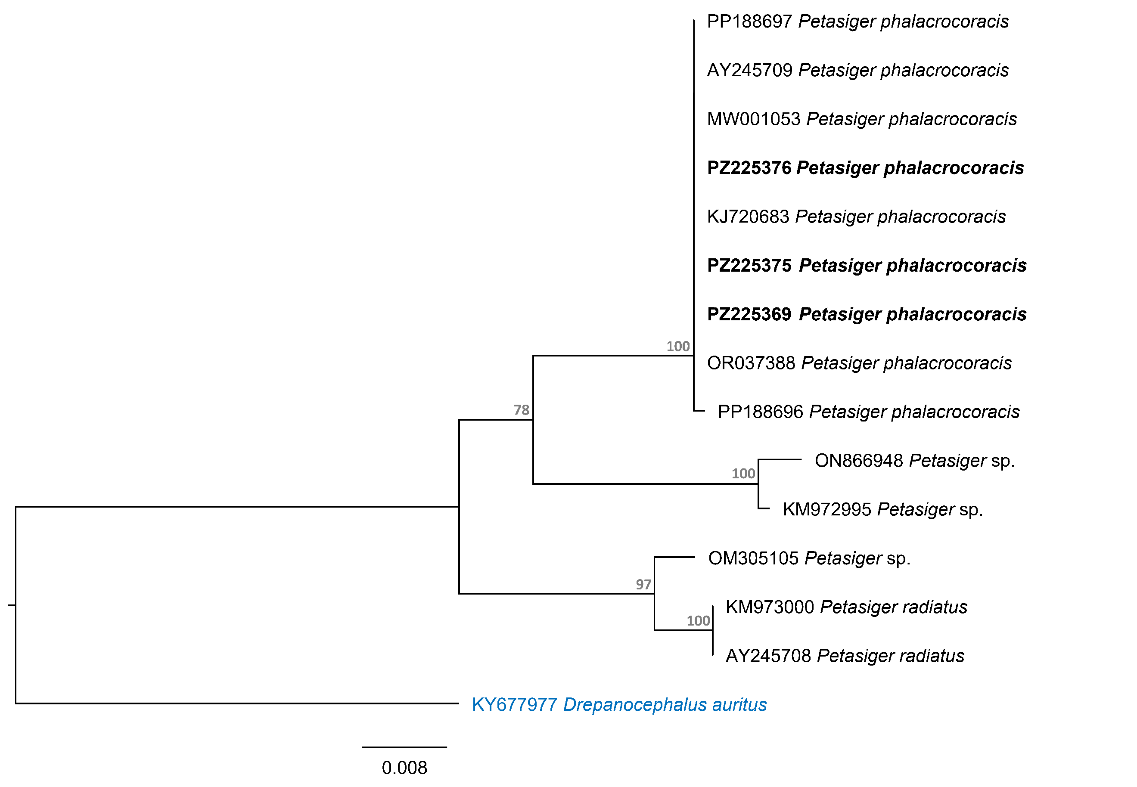

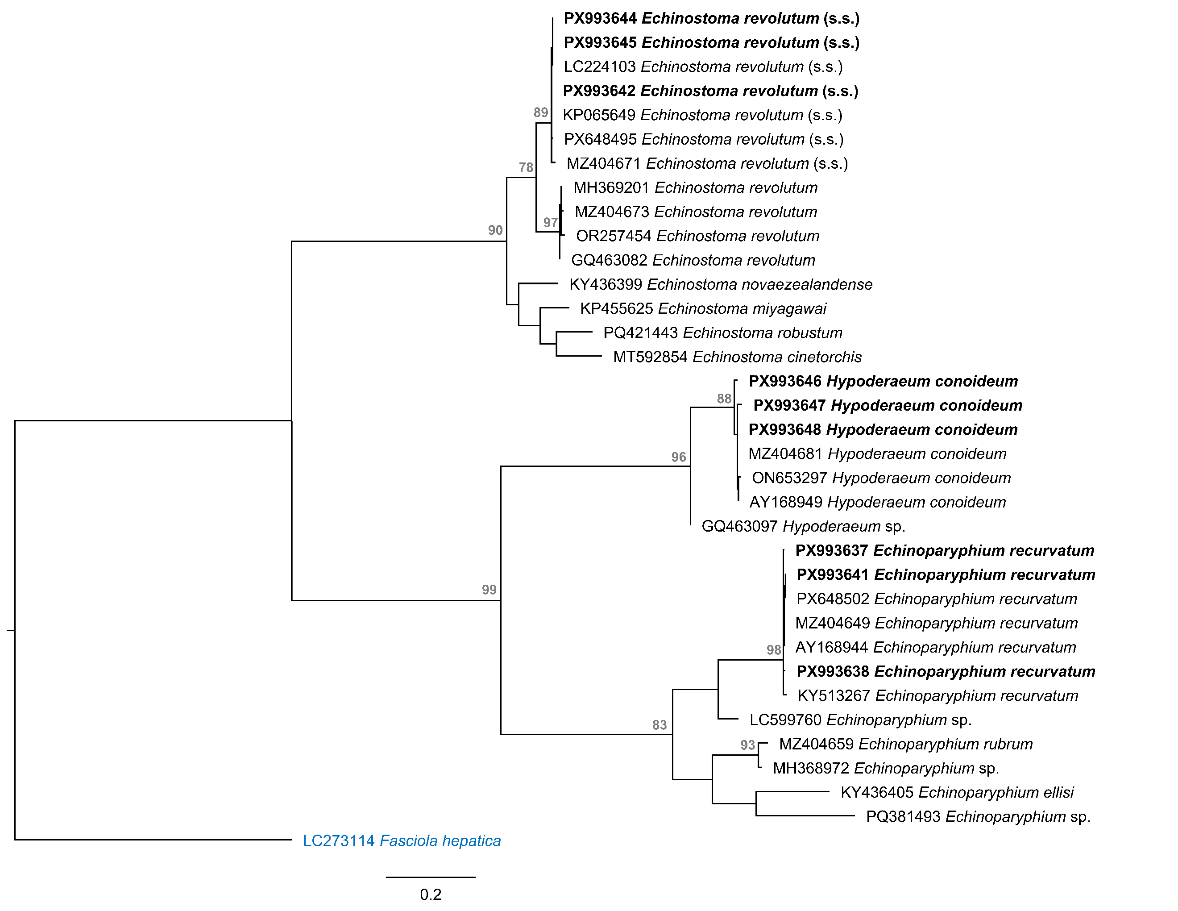
**Fig. S3.** Maximum likelihood (ML) phylogram based on the *nad*1 mtDNA alignment of Echinostomatidae. Novel sequences are highlighted in bold. Node support values >75 (1000 bootstrap replicates) are shown in grey. Scale bar indicates the expected number of substitutions per site, the outgroup is indicated in blue.

**Fig. S4.** Maximum likelihood (ML) phylogram based on the ITS1–5.8S–ITS2 rDNA alignment of Echinostomatidae (*Petasiger* spp.). Novel sequences are highlighted in bold. Node support values >75 (1000 bootstrap replicates) are shown in grey. Scale bar indicates the expected number of substitutions per site, the outgroup is indicated in blue.

**
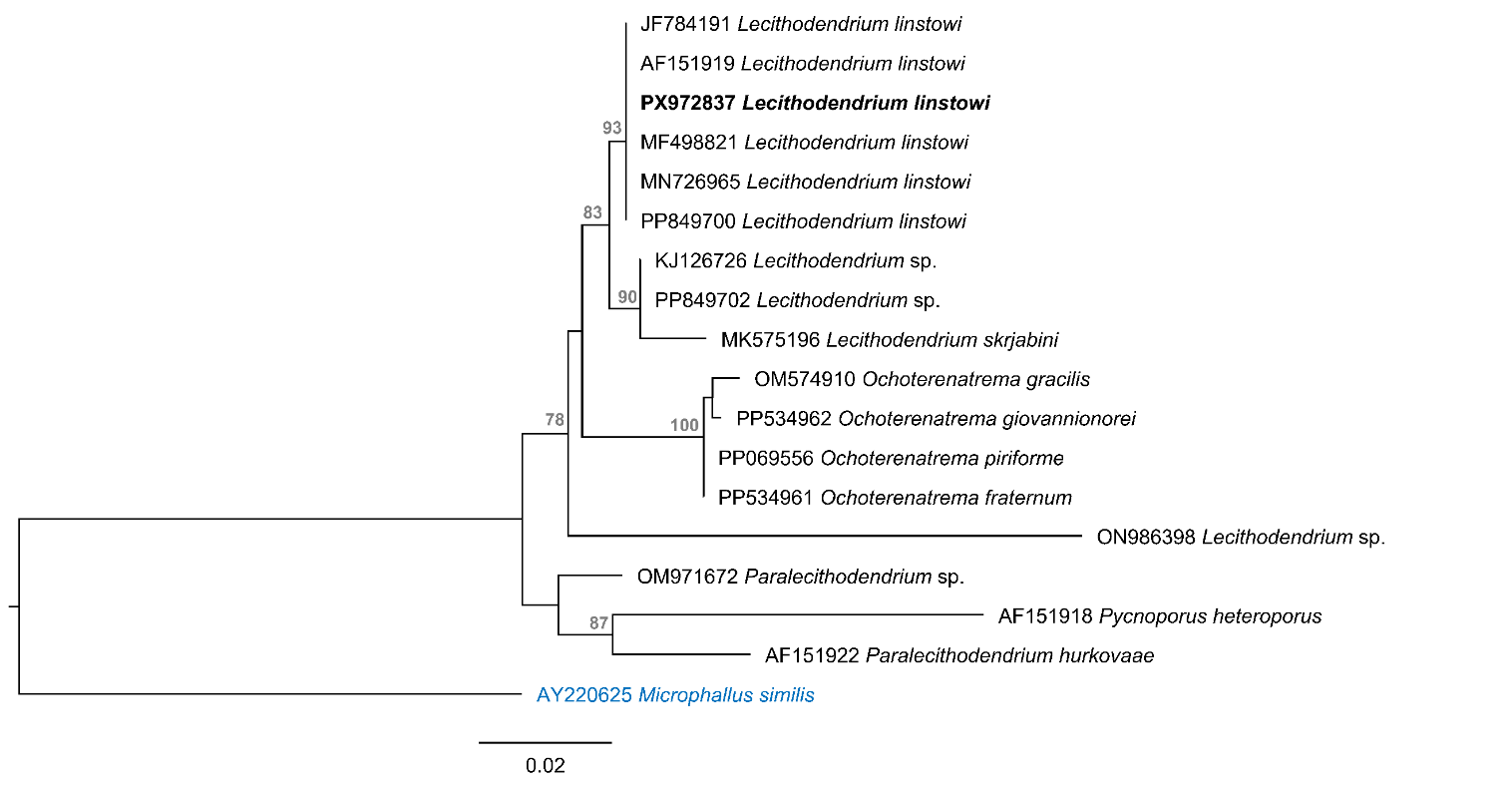

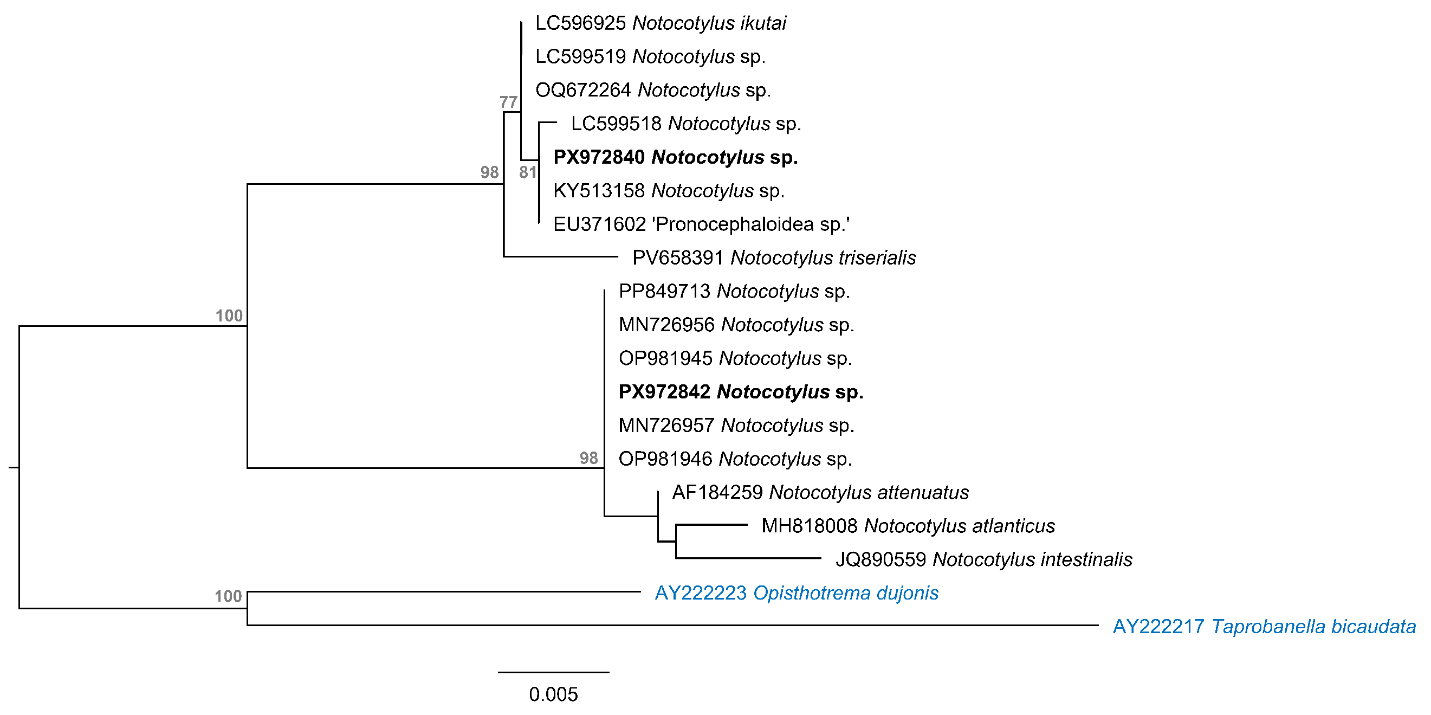
Fig. S5.** Maximum likelihood (ML) phylogram based on the 28S rDNA alignment of Lecithodendriidae. The novel sequence is highlighted in bold. Node support values >75 (1000 bootstrap replicates) are shown in grey. Scale bar indicates the expected number of substitutions per site, the outgroup is indicated in blue.

**Fig. S6.** Maximum likelihood (ML) phylogram based on the 28S rDNA alignment of Notocotylidae. Novel sequences are highlighted in bold. Node support values >75 (1000 bootstrap replicates) are shown in grey. Scale bar indicates the expected number of substitutions per site, the outgroup is indicated in blue.


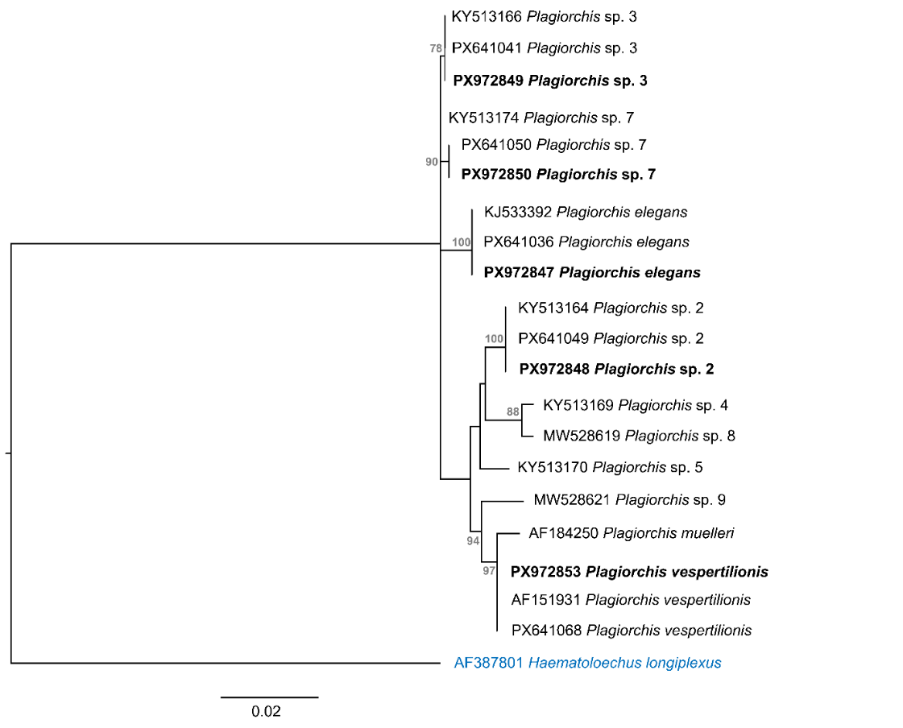


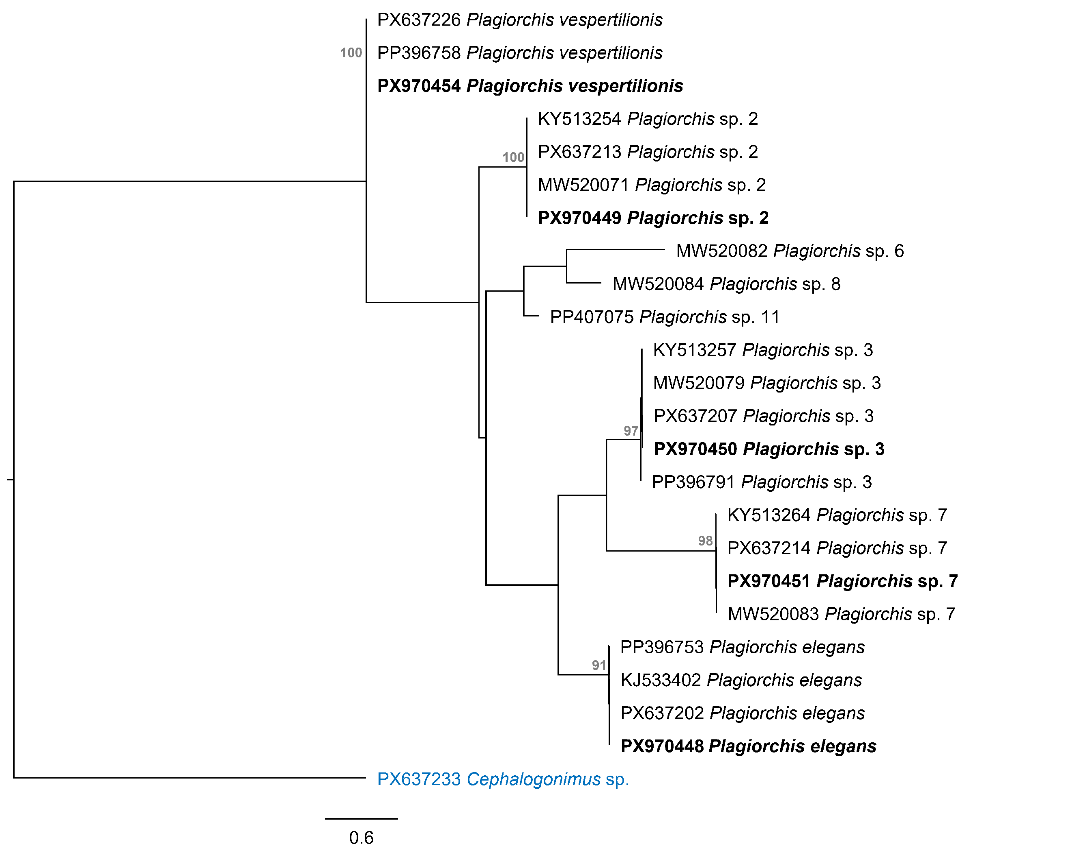
**Fig. S7.** Maximum likelihood (ML) phylogram based on the 28S rDNA alignment of Plagiorchiidae. Novel sequences are highlighted in bold. Node support values >75 (1000 bootstrap replicates) are shown in grey. Scale bar indicates the expected number of substitutions per site, the outgroup is indicated in blue.

**Fig. S8.** Maximum likelihood (ML) phylogram based on the *cox*1 mtDNA alignment of Plagiorchiidae. Novel sequences are highlighted in bold. Node support values >75 (1000 bootstrap replicates) are shown in grey. Scale bar indicates the expected number of substitutions per site, the outgroup is indicated in blue.


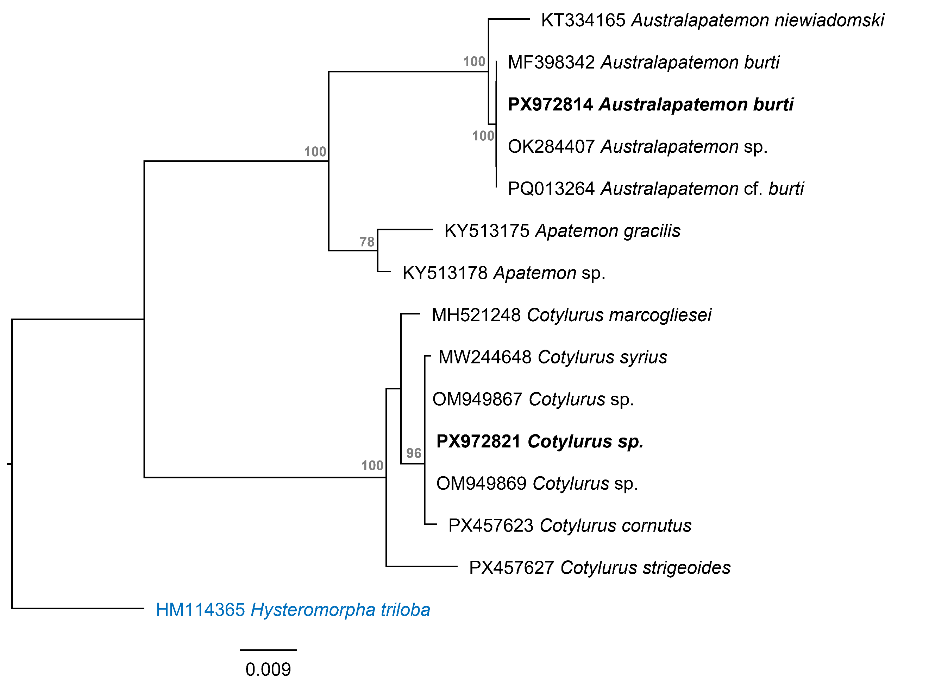


**
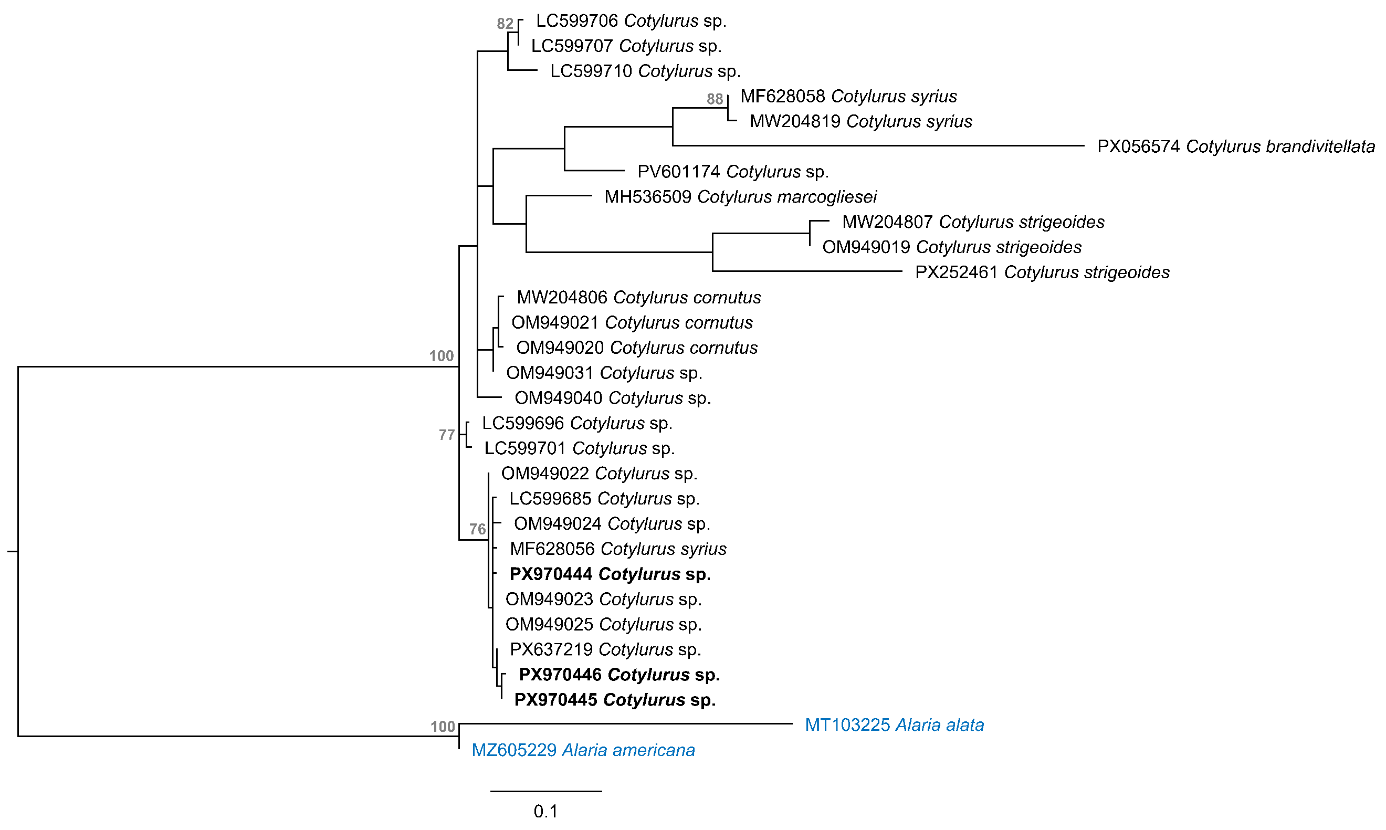
Fig. S9.** Maximum likelihood (ML) phylogram based on the 28S rDNA alignment of Strigeidae. Novel sequences are highlighted in bold. Node support values >75 (1000 bootstrap replicates) are shown in grey. Scale bar indicates the expected number of substitutions per site, the outgroup is indicated in blue.

**Fig. S10.** Maximum likelihood (ML) phylogram based on the *cox*1 mtDNA alignment of Strigeidae (*Cotylurus* spp.). Novel sequences are highlighted in bold. Node support values >75 (1000 bootstrap replicates) are shown in grey. Scale bar indicates the expected number of substitutions per site, the outgroup is indicated in blue.


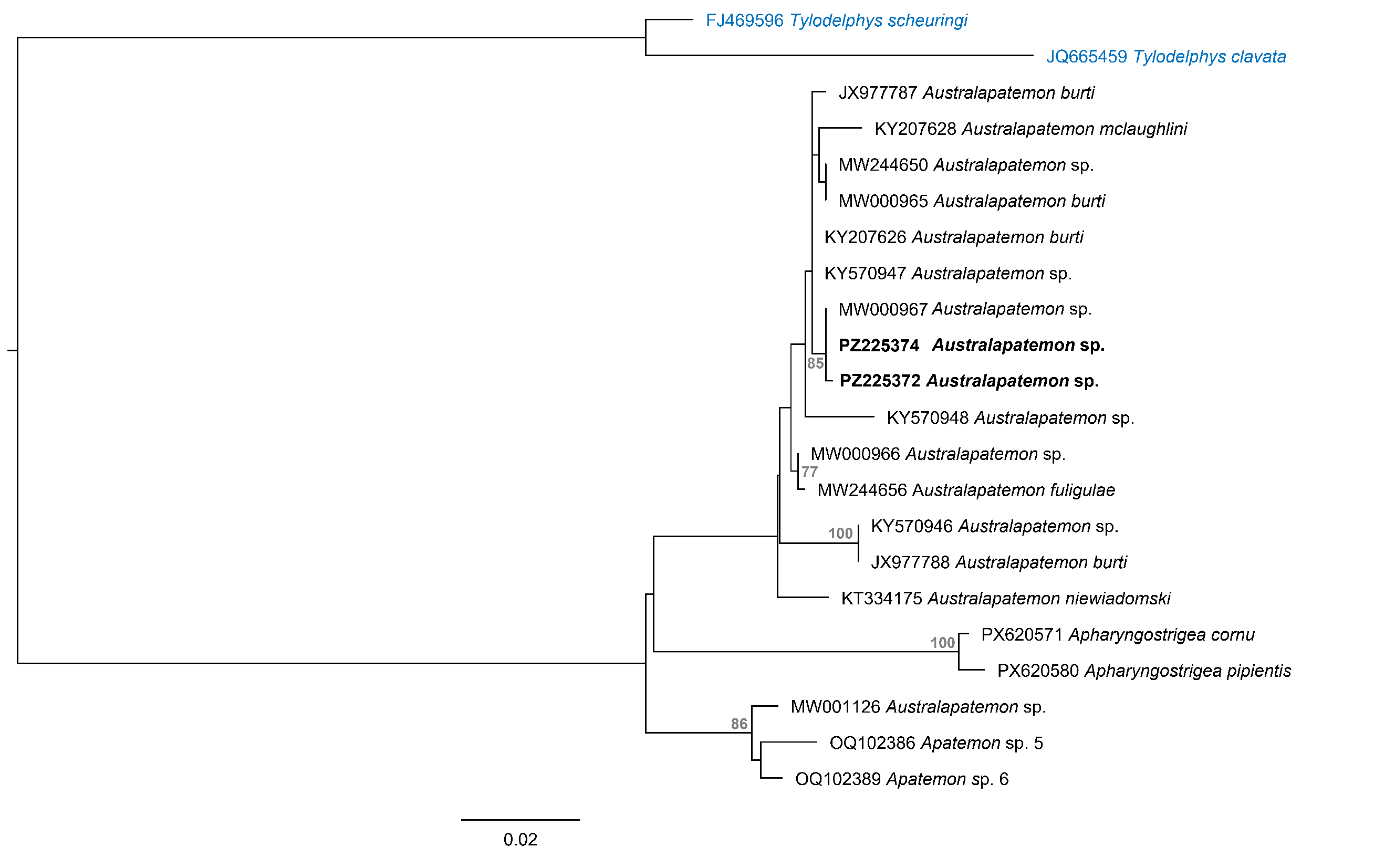
**Fig. S11.** Maximum likelihood (ML) phylogram based on the ITS1–5.8S–ITS2 rDNA alignment of Strigeidae (*Australapatemon* spp.). Novel sequences are highlighted in bold. Node support values >75 (1000 bootstrap replicates) are shown in grey. Scale bar indicates the expected number of substitutions per site, the outgroup is indicated in blue.

**References**

Achatz, T.J., Pulis, E.E., Junker, K., Binh, T.T., Snyder, S.D., Tkach, V.V. (2019). Molecular phylogeny of the Cyathocotylidae (Digenea, Diplostomoidea) necessitates systematic changes and reveals a history of host and environment switches. Zool Scr, 48, 545–556. https://doi.org/10.1111/zsc.12360.

Adema, C.M., Lun, C.M., Hanelt, B., Seville, R.S. (2009). Digenean trematode infections of native freshwater snails and invasive *Potamopyrgus antipodarum* in the Grand Teton National Park/John D. Rockefeller Memorial Parkway Area. J Parasitol, 95, 224–227. https://doi.org/10.1645/GE-1614.1.

Alberson, N.R., Rosser, T.G., Buddenborg, S.K., Khoo, L.H., Loker, E.S., Richardson, T.D., Woodyard, E.T., Wise, D.J., Pote, L.M., Griffin, M.J. (2017). North and South American haplotypes of *Drepanocephalus auritus* (Digenea: Echinostomatidae) are released from *Biomphalaria havanensis* (Mollusca: Planorbidae) inhabiting catfish aquaculture ponds in Mississippi, U.S.A. Comp Parasitol, 84, 87–101. https://doi.org/10.1654/1525-2647-84.2.87.

Amer, S., Maza, F. (2017). Direct submission.

Barton, D.P., Zhu, X., Nuhoglu, A., Pearce, L., McLellan, M., Shamsi, S. (2022). Parasites of Selected Freshwater Snails in the Eastern Murray Darling Basin, Australia. Int J Environ Res Public Health, 19, 7236. https://doi.org/10.3390/ijerph19127236.

Behrmann-Godel, J. (2013). Parasite identification, succession and infection pathways in perch fry (*Perca fluviatilis*): new insights through a combined morphological and genetic approach. Parasitology, 140, 509–520. https://doi.org/10.1017/S0031182012001989.

Besprozvannykh, V., Ngo, H., Nguyen, H., Hung, N., Rozhkovan, K., Ermolenko, A.V. (2013). Descriptions of digenean parasites from three snail species, *Bithynia fuchsiana* (Morelet), *Parafossarulus striatulus* Benson and *Melanoides tuberculata* Müller, in North Vietnam. Helminthologia 50, 190–204. 10.2478/s11687-013-0131-5.

Bilska-Zając, E., Marucci, G., Piróg-Komorowska, A., Cichocka, M., Różycki, M., Karamon, J., Sroka, J., Bełcik, A., Mizak, I., Cencek, T. (2021). Occurrence of *Alaria alata* in wild boars (*Sus scrofa*) in Poland and detection of genetic variability between isolates. Parasitol Res, 120, 83–91. https://doi.org/10.1007/s00436-020-06914-x.

Blasco-Costa, I., Poulin, R., Presswell, B. (2016). Species of *Apatemon* Szidat, 1928 and *Australapatemon* Sudarikov, 1959 (Trematoda: Strigeidae) from New Zealand: linking and characterising life cycle stages with morphology and molecules. Parasitol Res, 115, 271–289. https://doi.org/10.1007/s00436-015-4744-0.

Bouchard, É., Schurer, J.M., Kolapo, T., Wagner, B., Massé, A., Locke, S.A., Leighton, P., Jenkins, E.J. (2021). Host and geographic differences in prevalence and diversity of gastrointestinal helminths of foxes (*Vulpes vulpes*), coyotes (*Canis latrans*) and wolves (*Canis lupus*) in Québec, Canada. Int J Parasitol Parasites Wildl, 16, 126–137. https://doi.org/10.1016/j.ijppaw.2021.09.002.

Bowles, J., Blair, D., McManus, D.P. (1995). A molecular phylogeny of the human schistosomes. Molecular Phylog Evol, 4, 103–109. https://doi.org/10.1006/mpev.1995.1011.

Cech, G., Molnár, K., Székely, C. (2017). Molecular biological studies of adult and metacercarial stages of *Petasiger exaeretus* Dietz, 1909 (Digenea: Echinostomatidae). Acta Vet Hung, 65, 198–207. https://doi.org/10.1556/004.2017.020.

Detwiler, J.T., Bos, D.H., Minchella, D.J. (2010). Revealing the secret lives of cryptic species: Examining the phylogenetic relationships of echinostome parasites in North America. Mol Phylogenet Evol, 55, 611–620. https://doi.org/10.1016/j.ympev.2010.01.004.

Duan, Y., Al-Jubury, A., Kania, P.W., Buchmann, K. (2021). Trematode diversity reflecting the community structure of Danish freshwater systems: molecular clues. Parasites Vectors, 14, 43. https://doi.org/10.1186/s13071-020-04536-x.

Dumbo, C.J., Dos Santos, Q.M., Avenant-Oldewage, A. (2019). *Masenia nkomatiensis* n. sp. (Digenea: Cephalogonimidae) from *Clarias gariepinus* (Burchell) (Clariidae) in Incomati Bazin, Mozambique. Syst Parasitol, 96, 311–326. https://doi.org/10.1007/s11230-019-09848-w.

Dzikowski, R., Levy, M.G., Poore, M.F., Flowers, J.R., Paperna, I. (2004). Use of rDNA polymorphism for identification of Heterophyidae infecting freshwater fishes. Dis Aquat Org, 59, 35–41. https://doi.org/10.3354/dao059035.

Enabulele, E.E., Lawton, S.P., Walker, A.J., Kirk, R.S. (2018). Molecular and morphological characterization of the cercariae of *Lecithodendrium linstowi* (Dollfus, 1931), a trematode of bats, and incrimination of the first intermediate snail host, *Radix balthica*. Parasitology, 145, 307–312. https://doi.org/10.1017/S0031182017001640.

Enabulele, E.E., Lawton, S.P., Walker, A.J., Kirk, R.S. (2023). Molecular epidemiological analyses reveal extensive connectivity between *Echinostoma revolutum* (sensu stricto) populations across Eurasia and species richness of zoonotic echinostomatids in England. PLOS ONE, 18, e0270672. https://doi.org/10.1371/journal.pone.0270672.

Faltýnková, A., Kudlai, O., Pantoja, C., Jouet, D., Skírnisson, K. (2023). Prey-mimetism in cercariae of *Apatemon* (Digenea, Strigeidae) in freshwater in northern latitudes. Parasitol Res, 122, 815–831. https://doi.org/10.1007/s00436-023-07779-6.

Faltýnková, A., O'Dwyer, K., Pantoja, C., Jouet, D., Skírnisson, K., Kudlai, O. (2024). Trematode species diversity in the faucet snail, *Bithynia tentaculata* at the western edge of its native distribution, in Ireland. J Helminthol, 98, e52. https://doi.org/10.1017/S0022149X24000397.

Fernandes, T.F., Dos Santos, J.N., de Vasconcelos Melo, F.T., Achatz, T.J., McAllister, C.T., Carrion Bonilla, C., Tkach, V.V. (2022). Phylogenetic relationships of *Ochoterenatrema* Caballero, 1943 (Digenea: Lecithodendriidae) with descriptions of two new species. Parasitol Int, 89, 102575. https://doi.org/10.1016/j.parint.2022.102575.

Folmer, O., Black, M., Hoeh, W., Lutz, R., Vrijenhoek, R. (1994). DNA primers for amplification of mitochondrial cytochrome c oxidase subunit I from diverse metazoan invertebrates. Mol Mar Biol Biotechnol, 3, 294–299.

Gabrysiak, J., Kanarek, G., Rydelek, B., Wydra, S., Zaleśny, G., Hildebrand, J. (2025). Back from the dead: validity and taxonomic position of *Cotylurus brandivitellatus* (Belogurov, Maksimova et Tolkacheva, 1966) in light of the integrative taxonomy approach. Parasitology, 152, 1037–1046. https://doi.org/10.1017/S003118202510067X.

Galazzo, D.E., Dayanandan, S., Marcogliese, D.J., McLaughlin, J.D. (2002). Molecular systematics of some North American species of Diplostomum (Digenea) based on rDNA-sequence data comparisons with European congeners. Can J Zool, 80, 2207–2217. https://doi.org/10.1139/z02-198.

Georgieva, S., Blasco-Costa, I., Kostadinova, A. (2017). Molecular characterisation of four echinostomes (Digenea: Echinostomatidae) from birds in New Zealand, with descriptions of *Echinostoma novaezealandense* n. sp. and *Echinoparyphium poulini* n. sp. Syst. Parasitol, 94, 477–497. https://doi.org/10.1007/s11230-017-9712.

Georgieva, S., Faltýnková, A., Brown, R., Blasco-Costa, I., Soldánová, M., Sitko, J., Scholz, T., Kostadinova, A. (2014). *Echinostoma* *'revolutum'* (Digenea: Echinostomatidae) species complex revisited: species delimitation based on novel molecular and morphological data gathered in Europe. Parasites Vectors 7, 520. https://doi.org/10.1186/s13071-014-0520-8.

Georgieva, S., Selbach, C., Faltýnková, A., Soldánová, M., Sures, B., Skírnisson, K., Kostadinova, A. (2013). New cryptic species of the *'revolutum'* group of *Echinostoma* (Digenea: Echinostomatidae) revealed by molecular and morphological data. Parasites Vectors, 6, 64. https://doi.org/10.1186/1756-3305-6-64.

Gonchar, A., Jouet, D., Skírnisson, K., Krupenko, D., Galaktionov, K.V. (2019). Transatlantic discovery of *Notocotylus atlanticus* (Digenea: Notocotylidae) based on life cycle data. Parasitol Res, 118, 1445–1456. https://doi.org/10.1007/s00436-019-06297-8.

Gordy, M.A., Hanington, P.C. (2019). A fine-scale phylogenetic assessment of digenean trematodes in central Alberta reveals we have yet to uncover their total diversity. Ecol Evol, 9, 3153–3238. https://doi.org/10.1002/ece3.4939.

Gordy, M.A., Locke, S.A., Rawlings, T.A., Lapierre, A.R., Hanington, P.C. (2017). Molecular and morphological evidence for nine species in North American *Australapatemon* (Sudarikov, 1959): a phylogeny expansion with description of the zygocercous *Australapatemon* *mclaughlini* n. sp. Parasitol Res, 116, 2181–2198. https://doi.org/10.1007/s00436-017-5523-x.

Gyöngy, M., Juhász, L., Sellyei, B., Székely, C., Cech, G. (2024). Digenean trematodes (Trematoda: Digenea) parasitizing the digestive system of the great cormorant (*Phalacrocorax* *carbo*) in Hungary. Helminthologia, 61, 308–316. https://doi.org/10.2478/helm-2024-0033.

Heneberg, P., Sitko, J., Těšínský, M., Rząd, I., Bizos, J. (2018). Central European Strigeidae Railliet, 1919 (Trematoda: Strigeidida): Molecular and comparative morphological analysis suggests the reclassification of *Parastrigea* *robusta* Szidat, 1928 into *Strigea* Abildgaard, 1790. Parasitol Int, 67, 688–701. https://doi.org/10.1016/j.parint.2018.07.003.

Hernández-Mena, D.I., García-Prieto, L., García-Varela, M. (2014). Morphological and molecular differentiation of *Parastrigea* (Trematoda: Strigeidae) from Mexico, with the description of a new species. Parasitol Int, 63, 315–323. https://doi.org/10.1016/j.parint.2013.11.012.

Hernández-Mena, D.I., García-Varela, M., Pérez-Ponce de León, G. (2017). Filling the gaps in the classification of the Digenea Carus, 1863: systematic position of the Proterodiplostomidae Dubois, 1936 within the superfamily Diplostomoidea Poirier, 1886, inferred from nuclear and mitochondrial DNA sequences. Syst Parasitol, 94, 833–848. https://doi.org/10.1007/s11230-017-9745-1.

Hüsken, A., Schwelm, J., Rückert, S., Sures, B. (2025). Intermediate insights: tracing trematodes infecting amphibians via their first intermediate snail hosts. Parasites Vectors, 18, 285. https://doi.org/10.1186/s13071-025-06920-x.

Hüsken, A., Schwelm, J., Sures, B. (2026). Land use drives trematode dynamics in a restored stream system. Curr Res Parasitol Vector Borne Dis, 9, 100357. https://doi.org/10.1016/j.crpvbd.2026.100357.

Izrailskaia, A.V., Besprozvannykh, V.V., Tatonova, Y.V. (2021). *Echinostoma chankensis* nom. nov., other *Echinostoma* spp. and *Isthmiophora hortensis* in East Asia: morphology, molecular data and phylogeny within Echinostomatidae. Parasitology, 148, 1366–1382. https://doi.org/10.1017/S0031182021000950.

Johnson, P.T.J., Calhoun, D.M., Achatz, T.J., Greiman, S.E., Gestos, A., Keeley, W.H. (2024). Outbreak of parasite-induced limb malformations in a declining amphibian species in Colorado. Int J Parasitol Parasit Wildl, 24, 100965. https://doi.org/10.1016/j.ijppaw.2024.100965.

Kanarek, G., Gabrysiak, J., Pyrka, E., Jeżewski, W., Stanicka, A., Cichy, A., Żbikowska, E., Zaleśny, G., Hildebrand, J. (2023). Hyperparasitism among larval stages of Digenea in snail hosts: sophisticated life strategy or pure randomness? The scenario of *Cotylurus* sp. Zool J Linn Soc. https://doi.org/10.1093/zoolinnean%2Fzlad102.

Keller, S., Roderick, C. L., Caris, C., Grear, D. A., & Cole, R. A. (2021). Acute mortality in California tiger salamander (*Ambystoma californiense*) and Santa Cruz long-toed salamander (*Ambystoma macrodactylum croceum*) caused by *Ribeiroia ondatrae* (Class: Trematoda). Int J Parasitol Parasit Wildl, 16, 255–261. https://doi.org/10.1016/j.ijppaw.2021.10.008.

Khrebtova, I.S., Kondakov, A.V. (2022). Direct submission.

Kostadinova, A., Herniou, E.A., Barrett, J., Littlewood, D.T. (2003). Phylogenetic relationships of Echinostoma Rudolphi, 1809 (Digenea: Echinostomatidae) and related genera re-assessed via DNA and morphological analyses. Syst Parasitol, 54, 159–176. https://doi.org/10.1023/a:1022681123340.

Kudlai, O., Binkienė, R., Rakauskas, V., Baker, N.J. (2026). Comparison of historic and novel data reveals higher contemporary diversity of trematode metacercariae in freshwater fish. Parasite, 33. https://doi.org/10.1051/parasite/2025067.

Kudlai, O., Pantoja, C., O’Dwyer, K., Jouet, D., Skírnisson, K., Faltýnková, A. (2021). Diversity of *Plagiorchis* (Trematoda: Digenea) in high latitudes: Species composition and snail host spectrum revealed by integrative taxonomy. J Zool Syst Evol Res, 59, 937–962. https://doi.org/10.1111/jzs.12469.

Kudlai, O., Stunzenas, V., Tkach, V.V. (2015). The taxonomic identity and phylogenetic relationships of *Cercaria pugnax* and *C. helvetica* XII (Digenea: Lecithodendriidae) based on morphological and molecular data. Folia Parasitol, 62. http://dx.doi.org/10.14411/fp.2015.003.

Kundid, P., Pantoja, C., Janovcová, K., Soldánová, M. (2024). Molecular Diversity of the Genus *Plagiorchis* Lühe, 1899 in Snail Hosts of Central Europe with Evidence of New Lineages. Diversity, 16, 158. https://doi.org/10.3390/d16030158.

Le, T.H., Pham, L.T.K., Van Quyen, D., Nguyen, K.T., Doan, H.T.T., Saijuntha, W., Blair, D. (2024). The ribosomal transcription units of five echinostomes and their taxonomic implications for the suborder Echinostomata (Trematoda: Platyhelminthes). Parasitol Res, 123, 103. https://doi.org/10.1007/s00436-023-08110-z.

Littlewood, D.T.J., Curini-Galletti, M., Herniou, E.A. (2000). The interrelationships of proseriata (Platyhelminthes: seriata) tested with molecules and morphology. Mol Phylogenet Evol, 16, 449–466. https://doi.org/10.1006/mpev.2000.0802.

Littlewood, D.T.J., Rohde, K., Clough, K.A. (1997). Parasite speciation within or between host species? Phylogenetic evidence from site-specific polystome monogeneans. Int J Parasitol, 27, 1289–1297. https://doi.org/10.1016/s0020-7519(97)00086-6.

Locke, S.A., Van Dam, A., Caffara, M., Pinto, H.A., López-Hernández, D., Blanar, C. A. (2018). Validity of the Diplostomoidea and Diplostomida (Digenea, Platyhelminthes) upheld in phylogenomic analysis. Int J Parasitol, 48, 1043–1059. https://doi.org/10.1016/j.ijpara.2018.07.001.

López-Jiménez, A., García-Varela, M., Aguilar-Aguilar, R. (2025). Species delimitation of *Apharyngostrigea* Ciurea, 1927 (Digenea: Diplostomoidea) based on morphology and molecular data from the Neotropical region of Mexico. Parasitology, 1–15. Advance online publication. https://doi.org/10.1017/S0031182025101315.

Lord, J.S., Parker, S., Parker, F., Brooks, D.R. (2012). Gastrointestinal helminths of pipistrelle bats (*Pipistrellus pipistrellus*/*Pipistrellus pygmaeus*) (Chiroptera: Vespertilionidae) of England. Parasitology, 139, 366–374. https://doi.org/10.1017/S0031182011002046.

McPhail, B.A., Tomusiak, S., Veinot, H., Dodds, N., Hanington, P.C. (2025). Reclaimed wetlands support rich trematode and host diversity: findings from a four-year survey. Int J Parasitol, S0020-7519(25)00140-7. https://doi.org/10.1016/j.ijpara.2025.08.006.

McPhail, B.A., Veinot, H.E.S., Podruzny, A., Shen, N., Tomusiak, S., Dodds, N., Hanington, P.C. (2026). Integrating eDNA, molecular cercariometry, and snail surveys enhances characterization of digenetic trematode diversity. Parasitol Res, 125, 8. https://doi.org/10.1007/s00436-025-08622-w.

Mohanta, U.K., Watanabe, T., Anisuzzaman, Ohari, Y., Itagaki, T. (2019). Characterization of *Echinostoma revolutum* and *Echinostoma robustum* from ducks in Bangladesh based on morphology, nuclear ribosomal ITS2 and mitochondrial *nad*1 sequences. Parasitol Int, 69, 1–7. https://doi.org/10.1016/j.parint.2018.11.002.

Molnar, K., Gibson, D.I., Cech, G., Papp, M., Deak-Paulus, P., Juhasz, L., Toth, N., Szekely, C. (2015). The occurrence of metacercariae of *Petasiger* (Digenea: Echinostomatidae) in an unusual site, within the lateral line scales of cyprinid fishes. Folia Parasitol, 62, 2015.017. https://doi.org/10.14411/fp.2015.017.

Moszczynska, A., Locke, S.A., McLaughlin, J.D., Marcogliese, D.J., Crease, T.J. (2009). Development of primers for the mitochondrial cytochrome c oxidase I gene in digenetic trematodes (Platyhelminthes) illustrates the challenge of barcoding parasitic helminths. Mol Ecol Resour 9, Suppl s1, 75–82. https://doi.org/10.1111/j.1755-0998.2009.02634.x.

Nagataki, M., Tantrawatpan, C., Agatsuma, T., Sugiura, T., Duenngai, K., Sithithaworn, P., Andrews, R.H., Petney, T.N., Saijuntha, W. (2015). Mitochondrial DNA sequences of 37 collar-spined echinostomes (Digenea: Echinostomatidae) in Thailand and Lao PDR reveals presence of two species: *Echinostoma revolutum* and *E. miyagawai*. Infect Genet Evol, 35, 56–62. https://doi.org/10.1016/j.meegid.2015.07.022.

Nakao, M., Sasaki, M. (2021). Trematode diversity in freshwater snails from a stopover point for migratory waterfowls in Hokkaido, Japan: An assessment by molecular phylogenetic and population genetic analyses. Parasitol Int, 83, 102329. https://doi.org/10.1016/j.parint.2021.102329.

Nguyen, H.M., Greiman, S.E., Van Hoang, H., Ngoc, C.N., Van Nguyen, H., Pham, T.C., Madsen, H. (2022). The diversity of cercariae from freshwater snails in lowland areas of Binh Dinh and Phu Yen provinces, Vietnam. J Parasitol, 108, 601–612. https://doi.org/10.1645/21-125.

Olson, P.D., Cribb, T.H., Tkach, V.V., Bray, R.A., Littlewood, D.T. (2003). Phylogeny and classification of the Digenea (Platyhelminthes: Trematoda). Int J Parasitol. 33, 733–755. https://doi.org/10.1016/s0020-7519(03)00049-3.

Pantoja, C., Faltýnková, A., O'Dwyer, K., Jouet, D., Skírnisson, K., Kudlai, O. (2021). Diversity of echinostomes (Digenea: Echinostomatidae) in their snail hosts at high latitudes. Parasite, 28. https://doi.org/10.1051/parasite/2021054.

Pérez-Ponce de León, G., Hernández-Mena, D.I. (2019). Testing the higher-level phylogenetic classification of Digenea (Platyhelminthes, Trematoda) based on nuclear rDNA sequences before entering the age of the 'next-generation' Tree of Life. J Helminthol, 93, 260–276. https://doi.org/10.1017/S0022149X19000191.

Pulis, E., Tkach, V.V., Newman, R. (2011). Helminth parasites of the Wood Frog, *Lithobates sylvaticus*, in prairie pothole wetlands of the Northern Great Plains. Wetlands, 31, 675–685. 10.1007/s13157-011-0183-6.

Pyrka, E., Kanarek, G., Gabrysiak, J., Jeżewski, W., Cichy, A., Stanicka, A., Żbikowska, E., Zaleśny, G., Hildebrand, J. (2022). Life history strategies of *Cotylurus* spp. Szidat, 1928 (Trematoda, Strigeidae) in the molecular era - Evolutionary consequences and implications for taxonomy. Int J Parasitol Parasit Wildl, 18, 201–211. https://doi.org/10.1016/j.ijppaw.2022.06.002.

Pyrka, E., Kanarek, G., Zaleśny, G., Hildebrand, J. (2021). Leeches as the intermediate host for strigeid trematodes: genetic diversity and taxonomy of the genera *Australapatemon* Sudarikov, 1959 and *Cotylurus* Szidat, 1928. Parasites Vectors 14, 44. https://doi.org/10.1186/s13071-020-04538-9.

Ray, M., Trinidad, M., Francis, N., Shamsi, S. (2024). Characterization of *Echinostoma* spp. (Trematoda: Echinostomatidae Looss, 1899) infecting ducks in south-eastern Australia. Int J Food Microbiol, 421, 110754. https://doi.org/10.1016/j.ijfoodmicro.2024.110754.

Razo-Mendivil, U., Pérez-Ponce de León, G. (2011). Testing the evolutionary and biogeographical history of *Glypthelmins* (Digenea: Plagiorchiida), a parasite of anurans, through a simultaneous analysis of molecular and morphological data. Mol Phyl Evol, 59, 331–341. https://doi.org/10.1016/j.ympev.2011.02.018.

Sasaki, M., Kobayashi, M., Yoshino, T., Asakawa, M., Nakao, M. (2021). *Notocotylus ikutai* n. sp. (Digenea: Notocotylidae) from lymnaeid snails and anatid birds in Hokkaido, Japan. Parasitol Int, 83, 102318. https://doi.org/10.1016/j.parint.2021.102318.

Schumacher, G.A., Minchella, D.J. (2026). Diversity, host range, and specificity of echinostomes (Family Echinostomatidae; Looss, 1899) in the Midwestern United States. J Parasitol, 112, 170–182. https://doi.org/10.1645/24-138.

Schwelm, J., Kudlai, O., Smit, N.J., Selbach, C., Sures, B. (2020). High parasite diversity in a neglected host: larval trematodes of *Bithynia tentaculata* in Central Europe. J Helminthol, 94, e120. https://doi.org/10.1017/S0022149X19001093.

Seo, H., Ansai, E., Sase, T., Saito, T., Takano, T., Kojima, Y., Waki, T. (2024). Introduction of a snake trematode of the genus *Ochetosoma* in eastern Japan. Parasitol Int, 103, 102947. https://doi.org/10.1016/j.parint.2024.102947.

Shamsi, S., Banfield, A., Francis, N., Barton, D.P., McLellan, M. (2024). Characterisation of Nematoda and Digenea in selected Australian freshwater snails. J Invertebr Path, 204, 108116. https://doi.org/10.1016/j.jip.2024.108116.

Sokolov, S.G., Shchenkov, S.V., Kalmykov, A.P., Smirnova, A.D. (2020). Morphology and phylogenetic position of two microphalloid trematode species, parasites of the vesper bat *Pipistrellus kuhlii* in the Lower Volga Region of Russia. Zool Zhurnal 99, 261–274. https://doi.org/10.31857/S0044513420030125.

Soldánová, M., Georgieva, S., Roháčová, J., Knudsen, R., Kuhn, J.A., Henriksen, E.H., Siwertsson, A., Shaw, J.C., Kuris, A.M., Amundsen, P.A., Scholz, T., Lafferty, K.D., Kostadinova, A. (2017). Molecular analyses reveal high species diversity of trematodes in a sub-Arctic lake. Int J Parasitol 47, 327–345. https://doi.org/10.1016/j.ijpara.2016.12.008.

Snyder, S.D., Tkach, V.V. (2001). Phylogenetic and biogeographical relationships among some holarctic frog lung flukes (Digenea: Haematoloechidae). J Parasitol, 87, 1433–1440. https://doi.org/10.1645/0022-3395(2001)087[1433:PABRAS]2.0.CO;2.

Stunzenas, V. (2025). Direct submission.

Svinin, A.O., Chikhlyaev, I.V., Bashinskiy, I.W., Osipov, V.V., Neymark, L.A., Ivanov, A.Y., Stoyko, T.G., Chernigova, P.I., Ibrogimova, P.K., Litvinchuk, S.N., Ermakov, O.A. (2023). Diversity of trematodes from the amphibian anomaly P hotspot: Role of planorbid snails. PLOS ONE 18, e0281740. https://doi.org/10.1371/journal.pone.0281740.

Tandon, V., Athokpam, V.D., Jyrwa, D.B., Thapa, S. (2013). Direct submission.

Tkach, V.V., Gasperetti, R., Fernandes, T.F., Carrión-Bonilla, C.A., Cook, J.A., Achatz, T.J. (2024). Uncovering further diversity of *Ochoterenatrema* Caballero, 1943 (Digenea: Lecithodendriidae) in South American bats. Syst Parasitol 101, 43. https://doi.org/10.1007/s11230-024-10165-0

Tkach, V.V., Greiman, S., Snyder, S.D. (2010). On the phylogenetic relationships of the Liolopidae. Unpublished.

Tkach, V.V., Kudlai, O., Kostadinova, A. (2016). Molecular phylogeny and systematics of the Echinostomatoidea Looss, 1899 (Platyhelminthes: Digenea). Int J Parasitol, 46, 171–185. https://doi.org/10.1016/j.ijpara.2015.11.001.

Tkach, V.V., Littlewood, D.T.J., Olson, P.D., Kinsella, M., Zdzislaw, S. (2003). Molecular phylogenetic analysis of the Microphalloidea Ward, 1901 (Trematoda: Digenea). Syst Parasitol, 56, 1–15. https://doi.org/10.1023/A:1025546001611.

Tkach, V., Pawlowski, J., Mariaux, J. (2000). Phylogenetic analysis of the suborder plagiorchiata (Platyhelminthes, Digenea) based on partial lsrDNA sequences. Int J Parasitol, 30, 83–93. https://doi.org/10.1016/s0020-7519(99)00163-0.

Tkach, V., Pawlowski, J., Mariaux, J., Swiderski, Z. (2001b). Molecular phylogeny of the suborder Plagiorchiata and its position in the system of Digenea. In: Littlewood, D.T.J, Bray, R.A. (Eds.), Interrelationships of platyhelminthes. Taylor & Francis, London, 186–193.

Tkach, V.V., Snyder, S.D., Świderski, Z. (2001a). On the phylogenetic relationships of some members of Macroderoididae and Ochetosomatidae (Digenea, Plagiorchioidea). Acta Parasitol 46, 267–275.

Vainutis, K., Andreev, M., Voronova, A., Zyumchenko, N. (2023). Direct submission.

Vinogradova, A.A., Ataev, G.L., Isakova, N.P. and Prokhorova, E.E. (2022). Direct submission.

Vlasenkov, S.A., Yakovleva, G.A., Sokolov, S.G. (2025). Phylogenetic assessment of the type species of genus *Notocotylus* Diesing, 1839 (Digenea: Notocotylidae) and other congeners from some Palearctic wetland birds. J Helminthol, 99, e122. https://doi.org/10.1017/S0022149X25100849.

Zikmundová, J., Georgieva, S., Faltýnková, A., Soldánová, M., Kostadinova, A. (2014). Species diversity of *Plagiorchis* Lühe, 1899 (Digenea: Plagiorchiidae) in lymnaeid snails from freshwater ecosystems in central Europe revealed by molecules and morphology. Syst Parasitol, 88, 37–54. https://doi.org/10.1007/s11230-014-9481-8.
