# Supplementary material for: Trematode recolonization in first intermediate snail hosts after large-scale stream restoration
Source: Parasit Vectors. 2026 Jul 28;19:306. doi: 10.1186/s13071-026-07583-y (PMC13418816; doi:10.1186/s13071-026-07583-y)
Supplement: Supplementary file 2 — Additional file 2: Tables S5–S11. Summary of GLMM results for all snails and for A. balthica. Figs. S12–S13. Host richness and abundance and trematode richness and overall prevalence in relation to site distance from the river mouth for all snails and for A. balthica. Fig. S14. Temporal changes in community composition of trematodes in A. balthica. Fig. S15. Distance decay of trematode communities in A. balthica along the stream. Fig. S16. Prevalence of trematode species in the respective snail species identified in this study. Table S12. Trematode-host associations included in analyses on host-specificity in this study. Fig. S17. Maximum likelihood phylogram based on the cox1 mtDNA alignment for gastropod hosts included in analyses on host-specificity. Tables S13–S14. Regional and global phylogenetic host specificity and geographic host specificity of trematodes, with resulting classifications. [file 13071_2026_7583_MOESM2_ESM.docx]

**Additional file 2.**

**Tables S5–S11.** Summary of GLMM results for all snails (Tables S5–S8) and for *A. balthica* (Tables S9–S11).

**Figs. S12–S13.** Host richness and abundance (A, B) and trematode richness and overall prevalence (%) (C, D) in relation to site distance from the river mouth for all snails (Fig. S12) and for *A. balthica* (Fig. S13).

**Fig. S14.** Temporal changes in community composition (β-diversity) of trematodes in *A. balthica*.

**Fig. S15.** Distance decay of trematode communities in *A. balthica* along the stream*.*

**Fig. S16.** Prevalence of trematode species in the respective snail species identified in this study.

**Table S12.** Trematode-host associations included in analyses on host-specificity in this study.

**Fig. S17.** Maximum likelihood (ML) phylogram based on the *cox*1 mtDNA alignment for gastropod hosts included in analyses on host-specificity.

**Tables S13–S14.** Regional and global phylogenetic host specificity (PD, SES-PD) and geographic host specificity (β-specificity) of trematodes, with resulting classifications.

**Table S5.** Summary of GLMM results for snail abundance.

| Family: | nbinom2 (log) | | | | | | | | |
| --- | --- | --- | --- | --- | --- | --- | --- | --- | --- |
| Formula: | Host_abundance ~ Year + Season + Distance + (1 \| Year/Location_name) | | | | | | | | |
| AIC | | BIC | | logLik | | -2*log(L) | | df.resid | |
| 564.9 | | 585.2 | | -273.5 | | 546.9 | | 61 | |
| R2m R2c  delta 0.3187099 0.4553826  lognormal 0.3655075 0.5222485  trigamma 0.2610092 0.3729382 | | | | | | | | | |
| Fixed effects: | | | | | | | | | |
|  | | | Estimate | | Std. Error | | z-value | P-value | Sign. |
| (Intercept) | | | 2.72319 | | 0.57855 | | 4.707 | 2.51e-06 | *** |
| Year2024 | | | -0.29601 | | 0.55667 | | -0.532 | 0.594 |  |
| Year2025 | | | -0.36162 | | 0.56643 | | -0.638 | 0.695 |  |
| SeasonSpring | | | -0.96874 | | 0.27277 | | -3.551 | 0.0001 | *** |
| SeasonSummer | | | -0.02650 | | 0.00826 | | -0.339 | 0.795 |  |
| Distance | | | 0.02650 | | 0.00826 | | 3.209 | 0.001 | ** |
|  | | | | | | | | | |
| Contrast | | | Ratio | | Std. Error | | z-ratio | P-value | Sign. |
| Autumn/Spring | | | 2.635 | | 0.719 | | 2.551 | 0.001 | ** |
| Autumn/Summer | | | 1.091 | | 0.279 | | 0.339 | 0.939 |  |
| Spring/Summer | | | 0.414 | | 0.114 | | -3.191 | 0.004 | ** |

**Table S6.** Summary of GLMM results for snail species richness.

| Family: | poisson (log) | | | | | | | | |
| --- | --- | --- | --- | --- | --- | --- | --- | --- | --- |
| Formula: | Host_richness ~ Year + Season + Distance + (1 \| Year/Location_name) | | | | | | | | |
| AIC | | BIC | | logLik | | -2*log(L) | | df.resid | |
| 236.6 | | 254.5 | | -110.3 | | 220.6 | | 62 | |
| R2m R2c  delta 0.1232129 0.1232194  lognormal 0.1435784 0.1435859  trigamma 0.1024036 0.1024090 | | | | | | | | | |
| Fixed effects: | | | | | | | | | |
|  | | | Estimate | | Std. Error | | z-value | P-value | Sign. |
| (Intercept) | | | 1.078699 | | 0.418594 | | 2.577 | 0.009 | ** |
| Year2024 | | | -0.111526 | | 0.400111 | | -0.297 | 0.780 |  |
| Year2025 | | | -0.282539 | | 0.407137 | | -0.649 | 0.488 |  |
| SeasonSpring | | | -0.467880 | | 0.211282 | | -2.215 | 0.029 | * |
| SeasonSummer | | | 0.003586 | | 0.178823 | | 0.020 | 0.984 |  |
| Distance | | | 0.004696 | | 0.004670 | | 1.006 | 0.315 |  |
|  | | | | | | | | | |
| Contrast | | | Ratio | | Std. Error | | z-ratio | P-value | Sign. |
| Autumn/Spring | | | 1.597 | | 0.337 | | 2.214 | 0.069 |  |
| Autumn/Summer | | | 0.996 | | 0.178 | | -0.020 | 0.999 |  |
| Spring/Summer | | | 0.624 | | 0.132 | | -2.236 | 0.065 |  |

**Table S7.** Summary of GLMM results for overall trematode prevalence.

| Family: | binomial (logit) | | | | | | | | |
| --- | --- | --- | --- | --- | --- | --- | --- | --- | --- |
| Formula: | cbind(N_infected, N_hosts - N_infected) ~ Year + Season + Host_richness + Host_abundance + Distance + (1 \| Year/Location_name) | | | | | | | | |
| AIC | | BIC | | logLik | | -2*log(L) | | df.resid | |
| 177.8 | | 197.5 | | -79.9 | | 159.8 | | 57 | |
| R2m R2c  theoretical 0.14373039 0.8390155  delta 0.08889569 0.5189220 | | | | | | | | | |
| Fixed effects: | | | | | | | | | |
|  | | | Estimate | | Std. Error | | z-value | P-value | Sign. |
| (Intercept) | | | -2.711471 | | 0.699731 | | -3.875 | 0.0001 | *** |
| Year2025 | | | -0.303535 | | 0.445275 | | -0.682 | 0.495 |  |
| SeasonSpring | | | -0.497801 | | 0.438191 | | -1.136 | 0.256 |  |
| SeasonSummer | | | -0.366345 | | 0.276845 | | -1.323 | 0.186 |  |
| Host_richness | | | -0.149168 | | 0.110834 | | -1.346 | 0.178 |  |
| Host_abundance | | | 0.009084 | | 0.005182 | | 1.753 | 0.080 | . |
| Distance | | | 0.008218 | | 0.015280 | | 0.538 | 0.591 |  |
|  | | | | | | | | | |
| Contrast | | | Ratio | | Std. Error | | z-ratio | P-value | Sign. |
| Autumn/Spring | | | 1.645 | | 0.721 | | 1.136 | 0.492 |  |
| Autumn/Summer | | | 1.442 | | 0.399 | | 1.323 | 0.382 |  |
| Spring/Summer | | | 0.877 | | 0.352 | | -0.327 | 0.943 |  |

**Table S8.** Summary of GLMM results for trematode species richness.

| Family: | poisson (log) | | | | | | | | |
| --- | --- | --- | --- | --- | --- | --- | --- | --- | --- |
| Formula: | Trem_richness ~ Year + Season + Host_richness + Host_abundance + Distance  + (1 \| Year/Location_name) | | | | | | | | |
| AIC | | BIC | | logLik | | -2*log(L) | | df.resid | |
| 160.0 | | 179.7 | | -71.0 | | 142.0 | | 57 | |
| R2m R2c  delta 0.3559962 0.3560015  lognormal 0.4516491 0.4516558  trigamma 0.2415007 0.2415043 | | | | | | | | | |
| Fixed effects: | | | | | | | | | |
|  | | | Estimate | | Std. Error | | z-value | P-value | Sign. |
| (Intercept) | | | -1.579431 | | 0.552509 | | -2.859 | 0.004 | ** |
| Year2025 | | | -0.079615 | | 0.276037 | | -0.288 | 0.773 |  |
| SeasonSpring | | | 0.116925 | | 0.444835 | | 0.263 | 0.793 |  |
| SeasonSummer | | | 0.177636 | | 0.301913 | | 0.588 | 0.556 |  |
| Host_richness | | | 0.135918 | | 0.107085 | | 1.269 | 0.204 |  |
| Host_abundance | | | 0.028453 | | 0.004285 | | 6.640 | 3.13e-11 | *** |
| Distance | | | 0.003566 | | 0.009589 | | 0.372 | 0.710 |  |
|  | | | | | | | | | |
| Contrast | | | Ratio | | Std. Error | | z-ratio | P-value | Sign. |
| Autumn/Spring | | | 0.890 | | 0.396 | | -0.263 | 0.963 |  |
| Autumn/Summer | | | 0.837 | | 0.253 | | -0.588 | 0.826 |  |
| Spring/Summer | | | 0.941 | | 0.386 | | -0.148 | 0.988 |  |

**Table S9.** Summary of GLMM results for snail abundance (*A. balthica* only).

| Family: | nbinom2 (log) | | | | | | | | |
| --- | --- | --- | --- | --- | --- | --- | --- | --- | --- |
| Formula: | Host_abundance_AB ~ Year + Season + Distance + (1 \| Year/Location_name) | | | | | | | | |
| AIC | | BIC | | logLik | | -2*log(L) | | df.resid | |
| 474.8 | | 493.9 | | -228.4 | | 456.8 | | 53 | |
| R2m R2c  delta 0.3759275 0.4831438  lognormal 0.4393286 0.5646271  trigamma 0.2935242 0.3772387 | | | | | | | | | |
| Fixed effects: | | | | | | | | | |
|  | | | Estimate | | Std. Error | | z-value | P-value | Sign. |
| (Intercept) | | | -0.11993 | | 1.08314 | | -0.111 | 0.912 | *** |
| Year2024 | | | 1.98296 | | 1.06521 | | 1.862 | 0.063 |  |
| Year2025 | | | 1.63024 | | 1.07164 | | 1.521 | 0.128 |  |
| SeasonSpring | | | -0.46956 | | 0.33013 | | -1.442 | 0.155 |  |
| SeasonSummer | | | 0.33863 | | 0.32591 | | 1.039 | 0.299 |  |
| Distance | | | 0.03361 | | 0.01035 | | 3.248 | 0.001 | ** |
|  | | | | | | | | | |
| Contrast | | | Ratio | | Std. Error | | z-ratio | P-value | Sign. |
| Autumn/Spring | | | 1.599 | | 0.528 | | 1.422 | 0.329 |  |
| Autumn/Summer | | | 0.713 | | 0.232 | | -1.039 | 0.552 |  |
| Spring/Summer | | | 0.446 | | 0.150 | | -2.395 | 0.044 |  |

**Table S10.** Summary of GLMM results for overall trematode prevalence (*A. balthica* only).

| Family: | binomial (logit) | | | | | | | | |
| --- | --- | --- | --- | --- | --- | --- | --- | --- | --- |
| Formula: | cbind(N_infected_AB, N_hosts_AB - N_infected_AB) ~ Year + Season + Distance + Mean_size_AB + N_hosts_AB + Host_richness + (1 \| Year/Location_name) | | | | | | | | |
| AIC | | BIC | | logLik | | -2*log(L) | | df.resid | |
| 163.9 | | 184.9 | | -72.0 | | 143.9 | | 50 | |
| R2m R2c  theoretical 0.3758093 0.769123  delta 0.2333500 0.477569 | | | | | | | | | |
| Fixed effects: | | | | | | | | | |
|  | | | Estimate | | Std. Error | | z-value | P-value | Sign. |
| (Intercept) | | | -4.583194 | | 1.548156 | | -2.960 | 0.003 | ** |
| Year2025 | | | 0.028897 | | 0.391012 | | 0.074 | 0.941 |  |
| SeasonSpring | | | -1.009682 | | 0.437335 | | -2.309 | 0.021 | * |
| SeasonSummer | | | -0.711769 | | 0.278996 | | -2.551 | 0.011 | * |
| Distance | | | 0.011917 | | 0.014438 | | 0.825 | 0.409 |  |
| Mean_size_AB | | | 0.173065 | | 0.105503 | | 1.640 | 0.100 | . |
| N_hosts_AB | | | 0.002572 | | 0.004715 | | 0.545 | 0.585 |  |
| Host_richness | | | -0.007525 | | 0.112774 | | -0.067 | 0.947 |  |
|  | | | | | | | | | |
| Contrast | | | Ratio | | Std. Error | | z-ratio | P-value | Sign. |
| Autumn/Spring | | | 2.745 | | 1.200 | | 2.309 | 0.055 |  |
| Autumn/Summer | | | 2.038 | | 0.568 | | 2.551 | 0.029 |  |
| Spring/Summer | | | 0.742 | | 0.325 | | -0.681 | 0.775 |  |

**Table S11.** Summary of GLMM results for trematode species richness (*A. balthica* only).

| Family: | poisson (log) | | | | | | | | |
| --- | --- | --- | --- | --- | --- | --- | --- | --- | --- |
| Formula: | Trem_richness_AB ~ Year + Season + Distance + Mean_size_AB + N_hosts_AB + Host_richness + (1 \| Year/Location_name) | | | | | | | | |
| AIC | | BIC | | logLik | | -2*log(L) | | df.resid | |
| 145.3 | | 166.3 | | -62.7 | | 125.3 | | 50 | |
| R2m R2c  delta 0.4051693 0.4051863  lognormal 0.5012469 0.5012679  trigamma 0.2851353 0.2851472 | | | | | | | | | |
| Fixed effects: | | | | | | | | | |
|  | | | Estimate | | Std. Error | | z-value | P-value | Sign. |
| (Intercept) | | | -3.116375 | | 0.932292 | | -3.343 | 0.0008 | *** |
| Year2025 | | | 0.017971 | | 0.311978 | | 0.058 | 0.954 |  |
| SeasonSpring | | | -0.639876 | | 0.491667 | | -1.301 | 0.193 |  |
| SeasonSummer | | | 0.316446 | | 0.318039 | | -0.995 | 0.320 |  |
| Distance | | | 0.013611 | | 0.011375 | | 1.197 | 0.231 |  |
| Mean_size_AB | | | 0.150604 | | 0.067080 | | 2.245 | 0.025 | * |
| N_hosts_AB | | | 0.024799 | | 0.004461 | | 5.559 | 2.72e-08 | *** |
| Host_richness | | | 0.106962 | | 0.101948 | | 1.049 | 0.294 |  |
|  | | | | | | | | | |
| Contrast | | | Ratio | | Std. Error | | z-ratio | P-value | Sign. |
| Autumn/Spring | | | 1.896 | | 0.932 | | 1.301 | 0.394 |  |
| Autumn/Summer | | | 1.372 | | 0.436 | | 0.995 | 0.580 |  |
| Spring/Summer | | | 0.724 | | 0.343 | | -0.682 | 0.774 |  |

**

Fig. S12.** Host richness and abundance of all snails (A, B) and trematode richness and overall prevalence (%) (C, D) in relation to site distance from the river mouth. Points represent raw data of individual sampling events (site × month). Colored lines depict loess-estimated smoothing functions per year (2023-2025), with shaded areas indicating 95% confidence intervals.

**
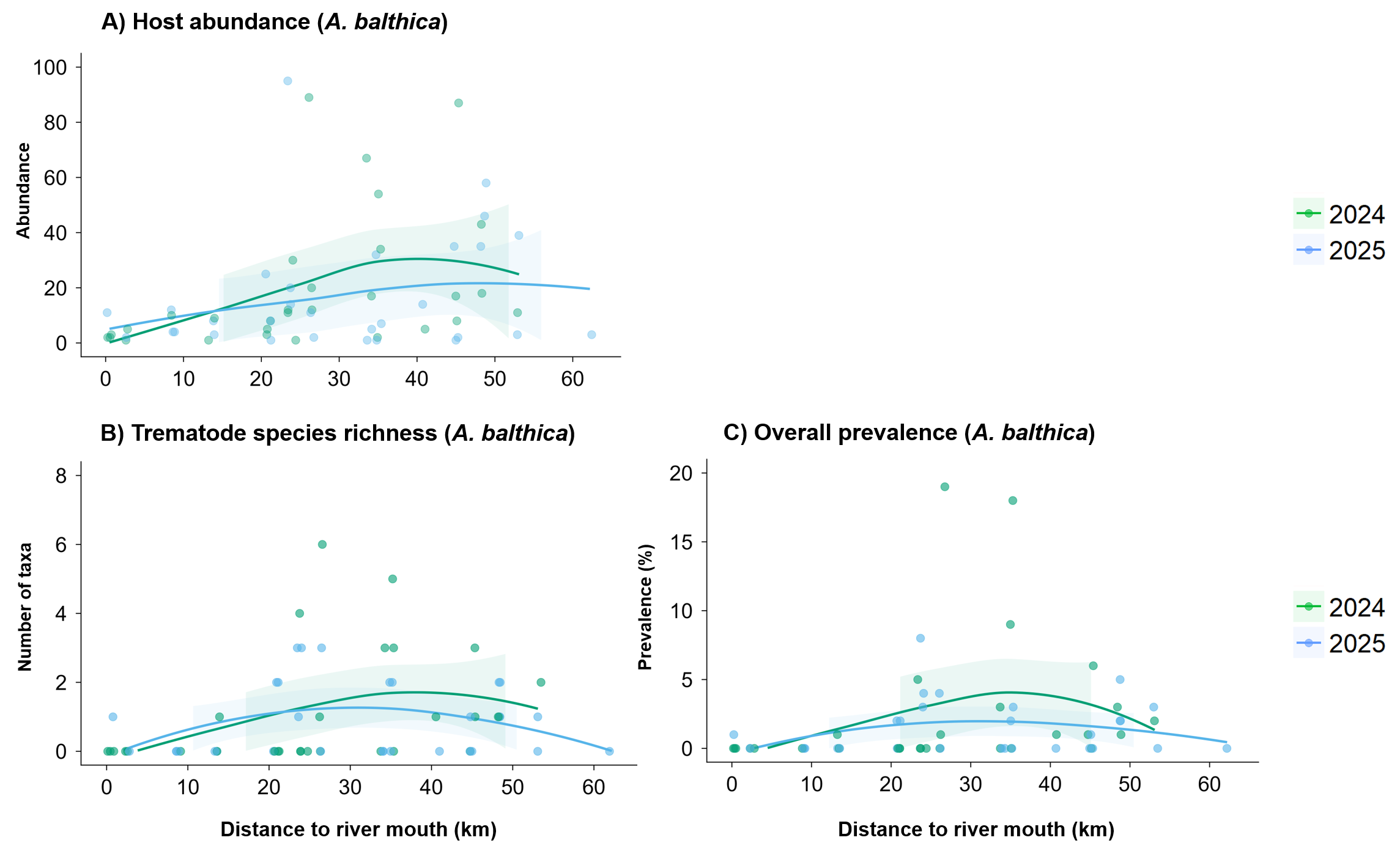
Fig. S13.** Abundance of all snails (*A. balthica*) (A) and trematode richness and overall prevalence (%) in *A. balthica* (B, C) in relation to site distance from the river mouth. Points represent raw data of individual sampling events (site × month). Colored lines depict loess-estimated smoothing functions per year (2024-2025), with shaded areas indicating 95% confidence intervals.

**
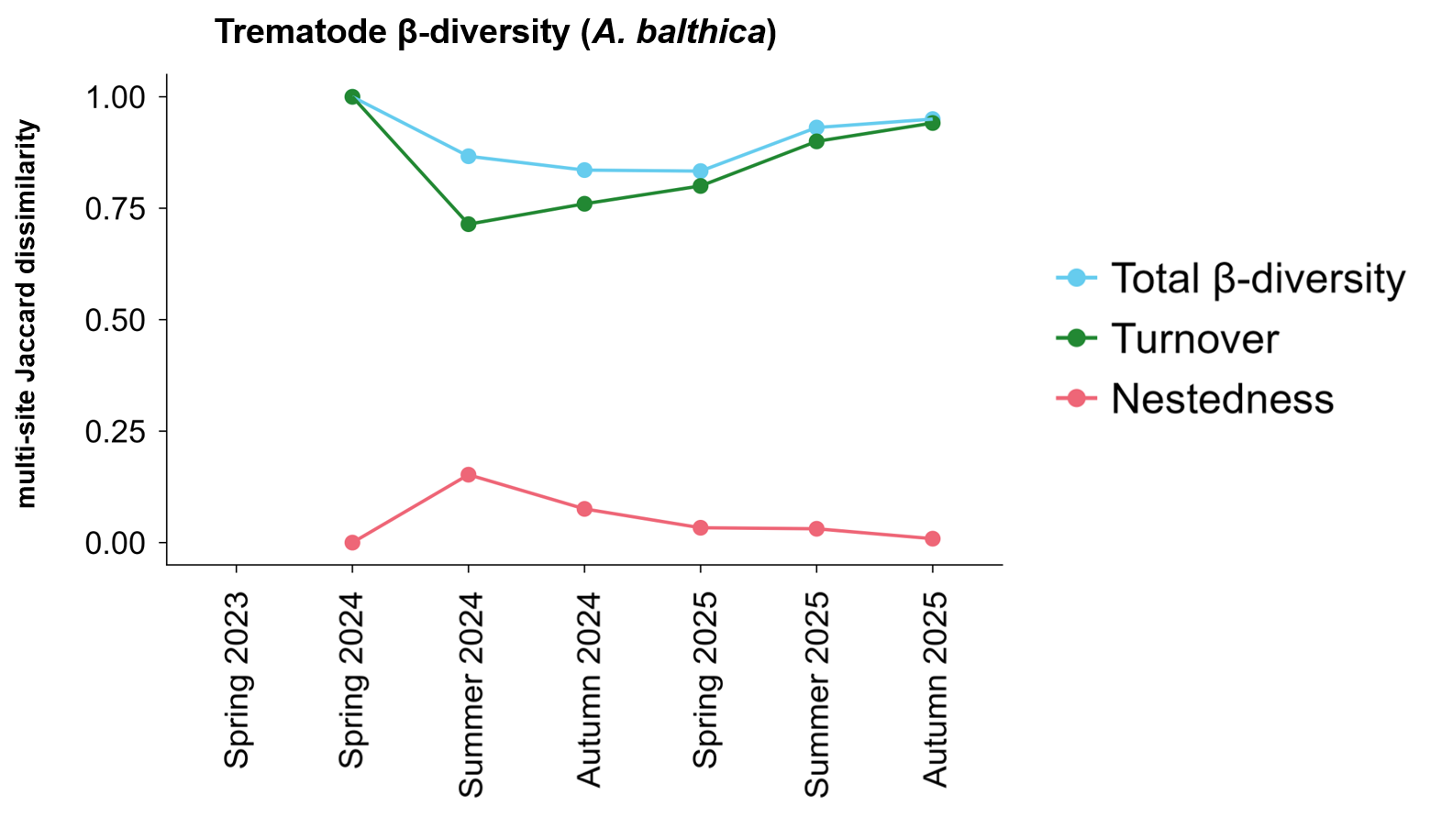
**

**Fig. S14.** Temporal changes in community composition (β-diversity) of trematodes in *A. balthica* based on multi-site Jaccard dissimilarity among sampling sites. Total β-diversity (β_JAC_) is partitioned into species turnover (β_JTU_) and nestedness-resultant dissimilarity (β_JNE_), where turnover reflects species replacement among sites and nestedness reflects the extent to which species-poor communities constitute subsets of species-rich communities.

**
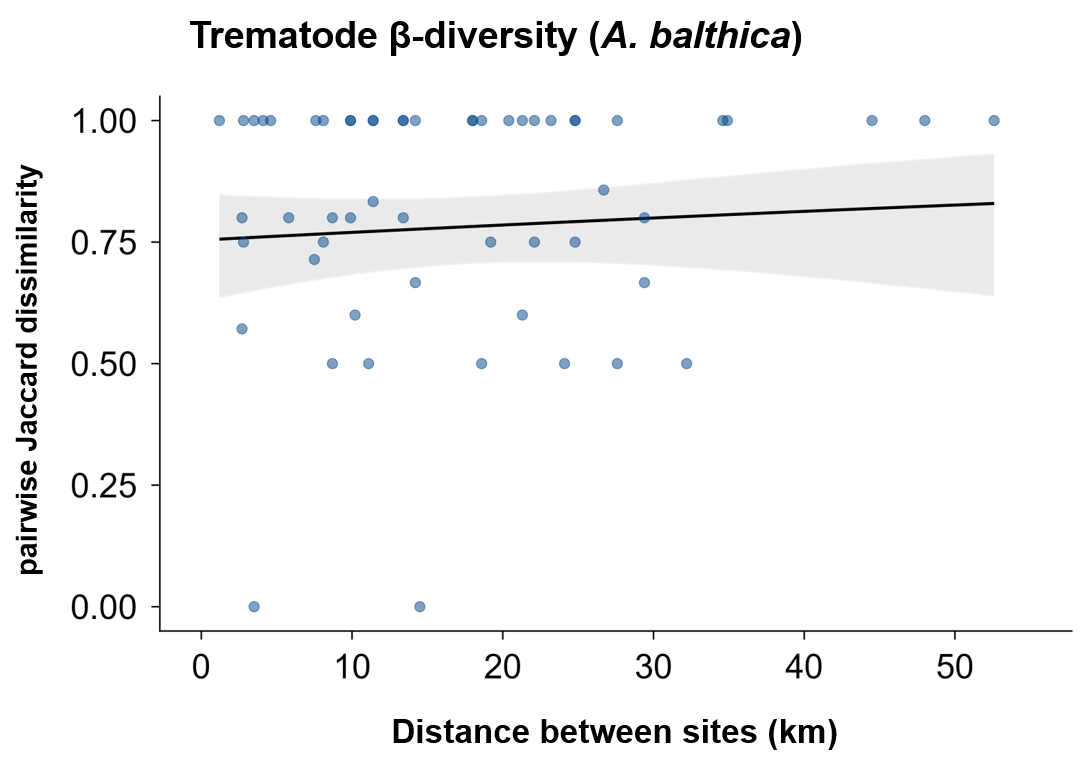
**

**Fig. S15.** Distance decay of trematode communities in *A. balthica* along the stream. Individual points represent pairwise Jaccard dissimilarity between sites within the same sampling time point. The solid line depicts predicted pairwise dissimilarity from mixed-effect models, with shaded areas indicating 95 % confidence intervals. No significant effect was detected for distance decay of trematode communities (p = 0.51).

**
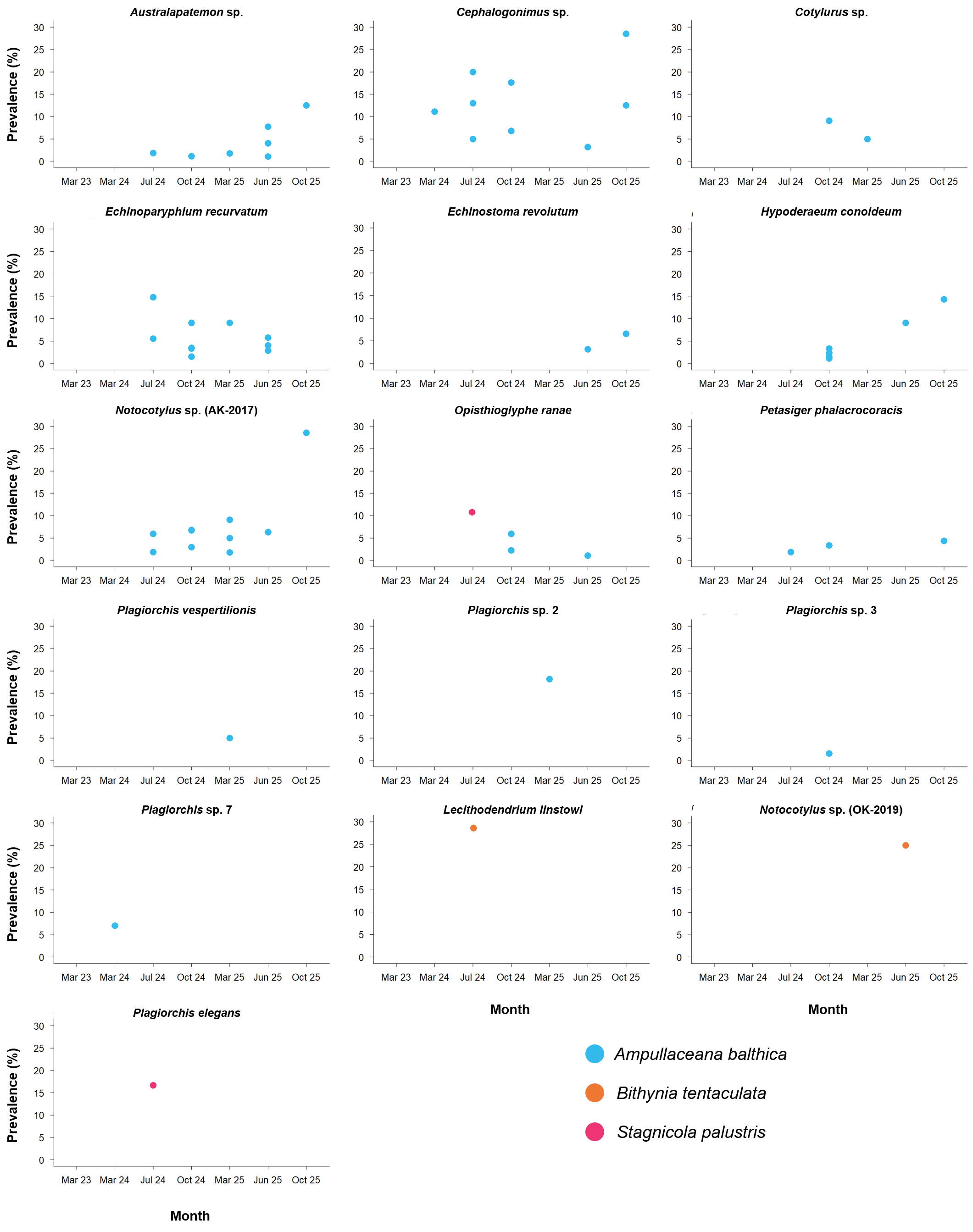
Fig. S16.** Prevalence of trematode species in the respective snail species identified in this study. Individual data points represent site × month combinations of the respective trematode species. Panels are sorted by snail host and then alphabetically.

**Table S12.** Trematode-host associations included in analyses on host-specificity in this study. Trematode-host associations included in local analyses are indicated in bold.

| **Trematode family and species** | **Location** | **Country** | **Accession number** | **Genetic marker** | **Host species** | **Accession number** | **References** |
| --- | --- | --- | --- | --- | --- | --- | --- |
| **Cephalogonimidae** | | | | | | | |
| *Cephalogonimus* sp. EM-2024 | **River Emscher** | **Germany** | **PX970442** | ***cox*1** | ***A. balthica*** | **PZ235457 ^b^** | **This study** |
|  | **River Boye** | **Germany** | **PX637233** | ***cox*1** | ***A. balthica*** | **PX919754 ^b^** | **Hüsken et al. (2026)** |
|  | River Rotbach | Germany | PV605649 | *cox*1 | *A. balthica* | PX919754 ^c^ | Hüsken et al. (2025, 2026) |
| **Echinostomatidae** | | | | | | | |
| *Echinoparyphium recurvatum* | **River Emscher** | **Germany** | **PX993635** | ***nad1*** | ***A. balthica*** | **PZ235457 ^b^** | **This study** |
|  | **River Boye** | **Germany** | **PX648493** | ***nad1*** | ***A. balthica*** | **PX919754 ^b^** | **Hüsken et al. (2026)** |
|  |  |  | **PX648497** | ***nad1*** | ***S. palustris*** | **PX919753 ^b^** |  |
|  |  |  | **PX648502** | ***nad1*** | ***P. carinatus*** | **PX919748 ^b^** |  |
|  | – | Iceland | MZ404647 | *nad1* | *A. balthica* | KM067657 ^c^ | Bolotov et al. (2017), Pantoja et al. (2021), Aksenova et al. (2024) |
|  | – | Finland | MZ404650 | *nad1* | *A. balthica* | KM067657 ^c^ |  |
|  |  |  | MZ404655 | *nad1* | *M. glutinosa* | ON603541 ^c^ |  |
|  | Lake Takvatn | Norway | KY513265 | *nad1* | *A. balthica* | KM067657 ^c^ | Bolotov et al. (2017), Soldánová et al. (2017) |
|  | Pen Ponds | England | ON653266 | *nad1* | *R. auricularia* | ON653347 ^a^ | Enabulele et al. (2023) |
|  | Tundry Pond | England | ON653270 | *nad1* | *R. auricularia* | ON653351 ^a^ |  |
|  | Hill Pond | England | ON653272 | *nad1* | *R. auricularia* | ON653353 ^a^ |  |
|  | Queen's River | England | ON653273 | *nad1* | *A. balthica* | ON653354 ^a^ |  |
|  | Peg Pond | England | ON653280 | *nad1* | *A. balthica* | ON653361 ^a^ |  |
|  | Rams Paddock Pond | England | ON653285 | *nad1* | *A. balthica* | ON653366 ^a^ |  |
|  | Wildlife Pond | England | ON653296 | *nad1* | *A. balthica* | ON653377 ^a^ |  |
| *Echinostoma revolutum* | **River Emscher** | **Germany** | **PX993642** | ***nad*1** | ***A. balthica*** | **PZ235457 ^b^** | **This study** |
|  | **River Boye** | **Germany** | **PX648495** | ***nad*1** | ***A. balthica*** | **PX919754 ^b^** | **Hüsken et al. (2026)** |
|  | – | Iceland | MZ404671 | *nad*1 | *A. balthica* | KM067657 ^c^ | Bolotov et al. (2017), Pantoja et al. (2021) |
|  | Pen Ponds | England | ON653232 | *nad*1 | *R. auricularia* | ON653313 ^a^ | Enabulele et al. (2023) |
|  | Tundry Pond | England | ON653241 | *nad*1 | *A. balthica* | ON653322 ^a^ |  |
|  | Hill Pond | England | ON653231 | *nad*1 | *R. auricularia* | ON653312 ^a^ |  |
|  | Peg Pond | England | ON653236 | *nad*1 | *A. balthica* | ON653317 ^a^ |  |
|  | Rams Paddock Pond | England | ON653238 | *nad*1 | *A. balthica* | ON653319 ^a^ |  |
|  | Bury Lake | England | ON653233 | *nad*1 | *R. auricularia* | ON653314 ^a^ |  |
|  | Wildlife Pond | England | ON653240 | *nad*1 | *A. balthica* | ON653321 ^a^ |  |
|  |  |  | ON653242 | *nad*1 | *S. palustris* | ON653323 ^a^ |  |
|  | Hengsteysee | Germany | KC618461 | *nad*1 | *S. palustris* | PX919753 ^b^ | Georgieva et al. (2013) |
|  |  |  | KC618454 | *nad*1 | *L. stagnalis* | PX919755 ^b^ |  |
|  | Baldeneysee | Germany | KP065652 | *nad*1 | *L. stagnalis* | PX919755 ^b^ | Georgieva et al. (2014) |
|  |  |  | KP065649 | *nad*1 | *L. stagnalis* | PX919755 ^b^ |  |
|  | Pond Hluboky´ u Hamru | Czech Republic | KP065657 | *nad*1 | *L. stagnalis* | PX919755 ^b^ |  |
|  | Krausenbechhofen | Germany | KP065656 | *nad*1 | *L. stagnalis* | PX919755 ^b^ |  |
|  | Lake Huumojärvi, Oulu | Finland | KP065655 | *nad*1 | *L. stagnalis* | PX919755 ^b^ |  |
| *Hypoderaeum conoideum* | **River Emscher** | **Germany** | **PX993646** | ***nad*1** | ***A. balthica*** | **PZ235457 ^b^** | **This study** |
|  | **River Boye** | **Germany** | **PX648499** | ***nad*1** | ***A. balthica*** | **PX919754 ^b^** | **Hüsken et al. (2026)** |
|  |  |  | **PX648505** | ***nad*1** | ***S. palustris*** | **PX919753 ^b^** |  |
|  |  |  | **PX648494** | ***nad*1** | ***L. stagnalis*** | **PX919755 ^b^** |  |
|  | – | Finland | MZ404681 | *nad*1 | *L. stagnalis* | OP084803 ^c^ | Pantoja et al. (2021), Aksenova et al. (2024) |
|  | Tundry Pond | England | ON653297 | *nad*1 | *R. auricularia* | ON653378 | Enabulele et al. (2023) |
|  | Rams Paddock Pond | England | ON653298 | *nad*1 | *A. balthica* | ON653379 |  |
| *Petasiger phalacrocoracis* | **River Emscher** | **Germany** | **PZ225369** | **ITS1-5.8S-ITS2** | ***A. balthica*** | **PZ235457 ^b^** | **This study** |
|  | Lake Furesoe | Denmark | MW001053 | ITS1-5.8S-ITS2 | *A. balthica* | PX919754 ^c^ | Duan et al. (2021), Hüsken et al. (2026) |
|  | Lake Esrum | Denmark | MW001052 | ITS1-5.8S-ITS2 | *A. balthica* | PX919754 ^c^ |  |
|  | Luchegorsk | Russia | OR037388 | ITS1-5.8S-ITS2 | *P. fontinalis* | MW600069 ^c^ | Menabit (2021),  Vainutis et al. (2023) |
| **Lecithodendriidae** | | | | | | | |
| *Lecithodendrium linstowi* | **River Emscher** | **Germany** | **PX972837** | **28S rDNA** | ***B. tentaculata*** | **PZ235464 ^b^** | **This study** |
|  | **River Boye** | **Germany** | **PX641056** | **28S rDNA** | ***B. tentaculata*** | **PZ235464 ^c^** | **Hüsken et al. (2026)** |
|  | K2 | Germany | MN726965 | 28S rDNA | *B. tentaculata* | MN720141 ^b^ | Schwelm et al. (2020) |
|  | Queen's River | England | MF498821 | 28S rDNA | *A. balthica* | MF498822 ^a^ | Enabulele et al. (2018) |
|  | Lough Corrib | Ireland | PP849700 | 28S rDNA | *B. tentaculata* | MW138435 ^c^ | Katokhin et al. (2020), Faltýnková et al. (2024) |
| **Notocotylidae** | | | | | | | |
| *Notocotylus* sp.  AK-2017 | **River Emscher** | **Germany** | **PX972838** | **28S rDNA** | ***A. balthica*** | **PZ235457 ^b^** | **This study** |
|  | **River Boye** | **Germany** | **PX641062** | **28S rDNA** | ***A. balthica*** | **PX919754 ^b^** | **Hüsken et al. (2026)** |
|  |  |  | **PX641066** | **28S rDNA** | ***S. palustris*** | **PX919753 ^b^** |  |
|  | Lake Takvatn | Norway | KY513158 | 28S rDNA | *A. balthica* | KM067657 ^c^ | Bolotov et al. (2017), Soldánová et al. (2017) |
| *Notocotylus* sp.  OK-2019 | **River Emscher** | **Germany** | **PX972842** | **28S rDNA** | ***B. tentaculata*** | **PZ235464 ^b^** | **This study** |
|  | K4 | Germany | MN726957 | 28S rDNA | *B. tentaculata* | MN720141 ^b^ | Schwelm et al. (2020) |
|  | Lough Corrib | Ireland | PP849713 | 28S rDNA | *B. tentaculata* | MW138435 ^c^ | Katokhin et al. (2020), Faltýnková et al. (2024) |
| **Plagiorchiidae** | | | | | | | |
| *Plagiorchis elegans* | **River Emscher** | **Germany** | **PX970448** | ***cox1*** | ***S. palustris*** | **PZ235481 ^b^** | **This study** |
|  | **River Boye** | **Germany** | **PX637202** | ***cox1*** | ***L. stagnalis*** | **PX919755 ^b^** | **Hüsken et al. (2026)** |
|  |  |  | **PX637216** | ***cox1*** | ***S. palustris*** | **PX919753 ^b^** |  |
|  | Lough Mask | Ireland | MW520066 | *cox1* | *S. fuscus* | LN515539 ^c^ | Schniebs et al. (2016), Kudlai et al. (2021), Aksenova et al. (2024) |
|  |  |  | MW519475 | *cox1* | *L. stagnalis* | OP084803 ^c^ |  |
|  | Lough Corrib | Ireland | MW519476 | *cox1* | *S. fuscus* | LN515539 ^c^ |  |
|  | Lake Konnevesi | Finland | MW519480 | *cox1* | *L. stagnalis* | OP084803 ^c^ |  |
|  | Danube near Gabcikovo | Slovakia | KJ533400 | *cox1* | *L. stagnalis* | PP203064 ^c^ | Zikmundová et al. (2014), Schols et al. (2024) |
|  | Pond Bohdanecsky | Czech Republic | KJ533413 | *cox1* | *L. stagnalis* | PP203064 ^c^ |  |
|  | Pond Hluboky u Hamru | Czech Republic | KJ533414 | *cox1* | *L. stagnalis* | PP203064 ^c^ |  |
|  | Milada | Czech Republic | PP396751 | *cox1* | *L. stagnalis* | PP203064 ^c^ | Kundid et al. (2024), Schols et al. (2024) |
| *Plagiorchis* sp. 2 | **River Emscher** | **Germany** | **PX970449** | ***cox1*** | ***A. balthica*** | **PZ235457 ^b^** | **This study** |
|  | **River Boye** | **Germany** | **PX637213** | ***cox1*** | ***A. balthica*** | **PX919754 ^b^** | **Hüsken et al. (2026)** |
|  | Lake Takvatn | Norway | KY513249 | *cox1* | *A. balthica* | KM067657 ^c^ | Bolotov et al. (2017), Soldánová et al. (2017) |
|  | Lake Konnevesi | Finland | MW519490 | *cox1* | *A. balthica* | KM067657 ^c^ | Bolotov et al. (2017), Kudlai et al. (2021) |
|  | Lake Myvatn | Iceland | MW519486 | *cox1* | *A. balthica* | KM067657 ^c^ |  |
|  | Lake Raudavatn | Iceland | MW520071 | *cox1* | *A. balthica* | KM067657 ^c^ |  |
| *Plagiorchis* sp. 3 | **River Emscher** | **Germany** | **PX970450** | ***cox1*** | ***A. balthica*** | **PZ235457 ^b^** | **This study** |
|  | **River Boye** | **Germany** | **PX637207** | ***cox1*** | ***A. balthica*** | **PX919754 ^b^** | **Hüsken et al. (2026)** |
|  | Lake Takvatn | Norway | KY513256 | *cox1* | *A. balthica* | KM067657 ^c^ | Bolotov et al. (2017), Soldánová et al. (2017) |
|  | Lough Corrib | Ireland | MW520076 | *cox1* | *A. balthica* | KM067657 ^c^ | Bolotov et al. (2017), Kudlai et al. (2021) |
|  | Lake Raudavatn | Iceland | MW520077 | *cox1* | *A. balthica* | KM067657 ^c^ |  |
|  | Medard | Czech Republic | PP396791 | *cox1* | *A. balthica* | PX919754 ^c^ | Kundid et al. (2024), Hüsken et al. (2026) |
| *Plagiorchis* sp. 7 | **River Emscher** | **Germany** | **PX970451** | ***cox1*** | ***A. balthica*** | **PZ235457 ^b^** | **This study** |
|  | **River Boye** | **Germany** | **PX637214** | ***cox1*** | ***A. balthica*** | **PX919754 ^b^** | **Hüsken et al. (2026)** |
|  | Lake Takvatn | Norway | KY513264 | *cox1* | *A. balthica* | KM067657 ^c^ | Bolotov et al. (2017), Soldánová et al. (2017) |
|  | Lough Corrib | Ireland | MW520083 | *cox1* | *A. balthica* | KM067657 ^c^ | Bolotov et al. (2017), Kudlai et al. (2021) |
| *Plagiorchis vespertilionis* | **River Emscher** | **Germany** | **PX970454** | ***cox1*** | ***A. balthica*** | **PZ235457 ^b^** | **This study** |
|  | **River Boye** | **Germany** | **PX637226** | ***cox1*** | ***A. balthica*** | **PX919754 ^b^** | **Hüsken et al. (2026)** |
|  | Medard | Czech Republic | PP396795 | *cox1* | *A. balthica* | PX919754 ^c^ | Vinarski et al. (2011), Bolotov et al. (2017), Kundid et al. (2024), Hüsken et al. (2026) |
|  | Barbora | Czech Republic | PP396758 | *cox1* | *R. auricularia* | FR797878 ^c^ |  |
|  | Otakar | Czech Republic | PP396760 | *cox1* | *R. auricularia* | FR797878 ^c^ |  |
|  | Most | Czech Republic | PP396784 | *cox1* | *A. lagotis* | KM067625 ^c^ |  |
| **Strigeidae** | | | | | | | |
| *Cotylurus* sp.  lineage II | **River Emscher** | **Germany** | **PX970444** | ***cox1*** | ***A. balthica*** | **PZ235457 ^b^** | **This study** |
|  | **River Boye** | **Germany** | **PX637219** | ***cox1*** | ***A. balthica*** | **PX919754 ^b^** | **Hüsken et al. (2026)** |
|  | – | Poland | OM949025 | *cox1* | *R. auricularia* | FR797878 ^c^ | Vinarski et al. (2011), Pyrka et al. (2022) |
|  |  |  | OM949022 | *cox1* | *R. labiata* | HG932227 ^c^ | Vinarski et al. (2014), Pyrka et al. (2022) |
| *Australapatemon* sp.  (*Australapatemon* cf. *burti*) | **River Emscher** | **Germany** | **PX637227** | ***cox1*** | ***A. balthica*** | **PZ235457 ^b^** | **This study** |
|  | **River Boye** | **Germany** | **PX637227** | ***cox1*** | ***A. balthica*** | **PX919754 ^b^** | **Hüsken et al. (2026)** |
|  |  |  | **–** | **–** | ***S. palustris*** | **PX919753 ^b^** |  |
|  |  |  | **–** | **–** | ***L. stagnalis*** | **PX919755 ^b^** |  |
|  |  |  | **PX637228** | ***cox1*** | ***P. carinatus*** | **PX919748 ^b^** |  |
| **Telorchiidae** | | | | | | | |
| *Opisthioglyphe ranae* | **River Emscher** | **Germany** | **PX972843** | **28S rDNA** | ***A. balthica*** | **PX919754 ^b^** | **This study** |
|  |  |  | **PV248801** | **28S rDNA** | ***S. palustris*** | **PZ235481 ^c^** | **Hüsken et al. (2025, 2026)** |
|  | **River Boye** | **Germany** | **PX641055** | **28S rDNA** | ***S. palustris*** | **PX919753 ^b^** | **Hüsken et al. (2026)** |

^a^ Exact host association, ^b^ Representative host from the same study, ^c^ Representative host from a different study.


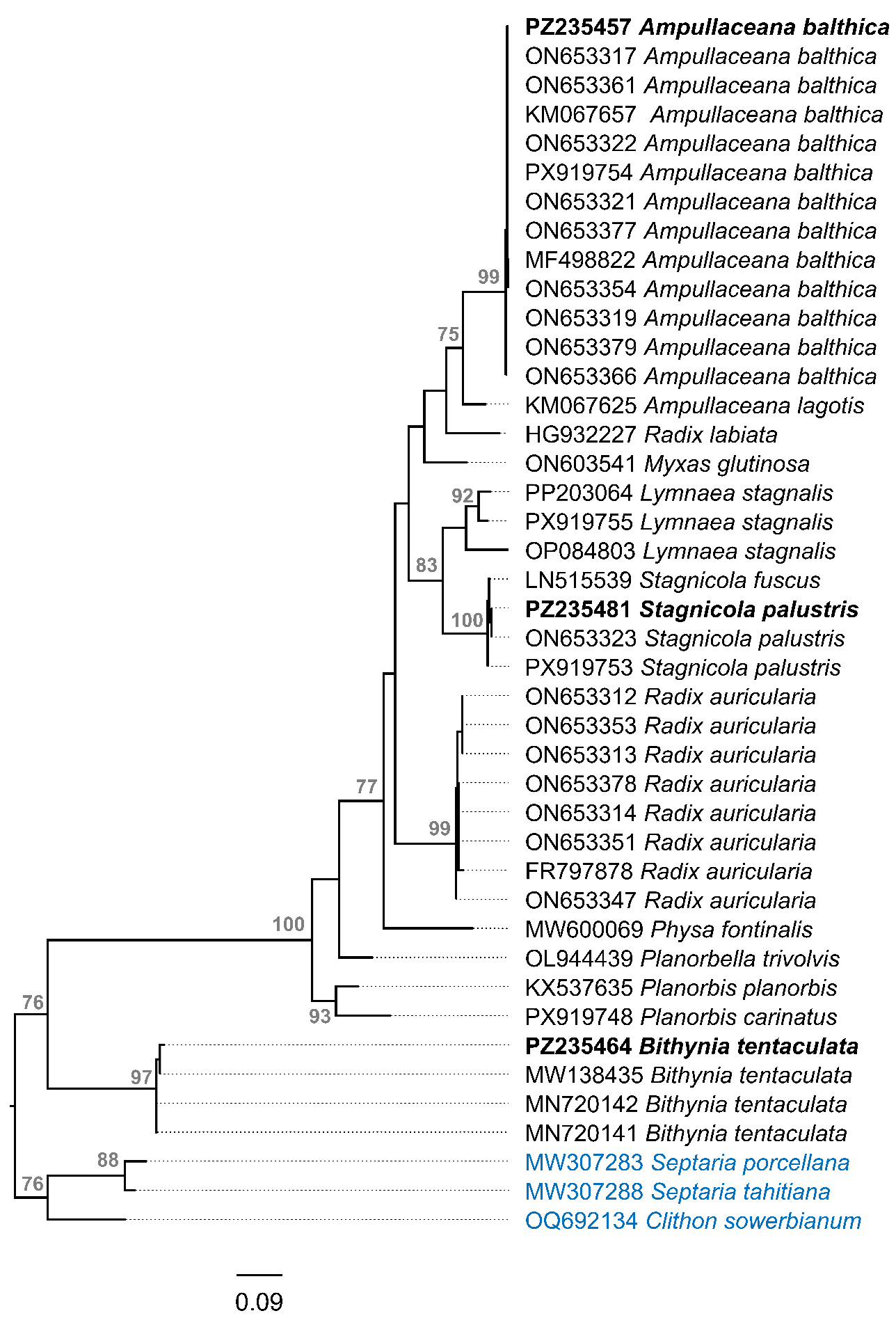
**Fig. S17.** Maximum likelihood (ML) phylogram based on the *cox*1 alignment (42 sequences, 434 bp, K3Pu+F+I+G4) for gastropod hosts included in analyses on host-specificity. Node support values >75 (1,000 bootstrap replicates) are shown in grey. Sequences generated in this study are presented in bold. Scale bar indicates the expected number of substitutions per site, outgroups are indicated in blue.

**Table S13.** Local phylogenetic host specificity (PD, SES-PD) and geographic host specificity (β-specificity) of trematodes, with resulting classifications. Trematode taxa were classified as phylogenetic specialists (SES-PD ≤ -1), generalists (SES-PD ≥ 1), or intermediate. Trematode taxa were classified into geographic host specialist, intermediate, and generalist categories based on tertiles of β-specificity values.

| **Trematode family and species** | **No. locations** | **No. host species** | **PD** | **SES-PD** | **Sign.** | **SES-PD classification** | **β-specificity** | **β-specificity classification** |
| --- | --- | --- | --- | --- | --- | --- | --- | --- |
| **Cephalogonimidae** | | | | | | | | |
| *Cephalogonimus* sp. EM-2024 | 2 | 1 | 0 | -1.32 | n.s. | Specialist | 0 | Specialist |
| **Echinostomatidae** | | | | | | | | |
| *Echinoparyphium recurvatum* | 2 | 4 | 0.78 | -0.78 | n.s. | Intermediate | 0.75 | Generalist |
| *Echinostoma revolutum* | 2 | 1 | 0 | -1.32 | n.s. | Specialist | 0 | Specialist |
| *Hypoderaeum conoideum* | 2 | 3 | 0.44 | -1.22 | n.s. | Specialist | 0.77 | Generalist |
| *Petasiger phalacrocoracis* | 1 | 1 | 0 | – | – | Specialist | 0 | Specialist |
| **Lecithodendriidae** | | | | | | | | |
| *Lecithodendrium linstowi* | 2 | 1 | 0.02 | -1.27 | n.s. | Specialist | 0 | Specialist |
| **Notocotylidae** | | | | | | | | |
| *Notocotylus* sp. AK-2017 | 2 | 2 | 0.35 | -0.88 | n.s. | Intermediate | 0.5 | Intermediate |
| *Notocotylus* sp. OK-2019 | 1 | 1 | 0 | – | – | Specialist | 0 | Specialist |
| **Plagiorchiidae** | | | | | | | | |
| *Plagiorchis elegans* | 2 | 2 | 0.18 | -1.35 | n.s. | Specialist | 0.5 | Intermediate |
| *Plagiorchis* sp. 2 | 2 | 1 | 0 | -1.29 | n.s. | Specialist | 0 | Specialist |
| *Plagiorchis* sp. 3 | 2 | 1 | 0 | -1.28 | n.s. | Specialist | 0 | Specialist |
| *Plagiorchis* sp. 7 | 2 | 1 | 0 | -1.25 | n.s. | Specialist | 0 | Specialist |
| *Plagiorchis vespertilionis* | 2 | 1 | 0 | -1.31 | n.s. | Specialist | 0 | Specialist |
| **Strigeidae** | | | | | | | | |
| *Cotylurus* sp. lineage II | 2 | 1 | 0 | -1.30 | n.s. | Specialist | 0 | Specialist |
| *Australapatemon* cf. *burti* | 2 | 4 | 0.78 | -0.84 | n.s. | Intermediate | 0.75 | Generalist |
| **Telorchiidae** | | | | | | | | |
| *Opisthioglyphe ranae* | 2 | 2 | 0.36 | -0.95 | n.s. | Intermediate | 0.5 | Intermediate |

**Table S14.** Global phylogenetic host specificity (PD, SES-PD) and geographic host specificity (β-specificity) of trematodes, with resulting classifications. Trematode taxa were classified as phylogenetic specialists (SES-PD ≤ -1), generalists (SES-PD ≥ 1), or intermediate. Trematode taxa were classified into geographic host specialist, intermediate, and generalist categories based on tertiles of β-specificity values.

| **Trematode family and species** | **No. locations** | **No. host species** | **PD** | **SES-PD** | **Sign.** | **SES-PD classification** | **β-specificity** | **β-specificity classification** |
| --- | --- | --- | --- | --- | --- | --- | --- | --- |
| **Cephalogonimidae** | | | | | | | | |
| *Cephalogonimus* sp. EM-2024 | 3 | 1 | 0 | – | – | Specialist | 0 | Specialist |
| **Echinostomatidae** | | | | | | | | |
| *Echinoparyphium recurvatum* | 12 | 6 | 1.01 | -3.4 | n.s. | Specialist | 0.91 | Generalist |
| *Echinostoma revolutum* | 16 | 4 | 0.69 | -4.70 | n.s. | Specialist | 0.93 | Generalist |
| *Hypoderaeum conoideum* | 5 | 4 | 0.67 | -1.71 | n.s. | Specialist | 0.87 | Generalist |
| *Petasiger phalacrocoracis* | 4 | 2 | 0.42 | -0.83 | n.s. | Intermediate | 0.72 | Generalist |
| **Lecithodendriidae** | | | | | | | | |
| *Lecithodendrium linstowi* | 5 | 2 | 1.13 | 0.04 | n.s. | Intermediate | 0.73 | Generalist |
| **Notocotylidae** | | | | | | | | |
| *Notocotylus* sp. AK-2017 | 3 | 2 | 0.35 | -1.43 | n.s. | Specialist | 0.50 | Intermediate |
| *Notocotylus* sp. OK-2019 | 3 | 1 | 0.02 | -1.80 | n.s. | Specialist | 0 | Specialist |
| **Plagiorchiidae** | | | | | | | | |
| *Plagiorchis elegans* | 9 | 3 | 0.30 | -2.22 | n.s. | Specialist | 0.84 | Generalist |
| *Plagiorchis* sp. 2 | 6 | 1 | 0.002 | -1.80 | n.s. | Specialist | 0 | Specialist |
| *Plagiorchis* sp. 3 | 7 | 1 | 0.002 | -1.87 | n.s. | Specialist | 0 | Specialist |
| *Plagiorchis* sp. 7 | 4 | 1 | 0.002 | -1.72 | n.s. | Specialist | 0 | Specialist |
| *Plagiorchis vespertilionis* | 6 | 3 | 0.40 | -1.26 | n.s. | Specialist | 0.88 | Generalist |
| **Strigeidae** | | | | | | | | |
| *Cotylurus* sp. lineage II | 4 | 3 | 0.46 | 1.13 | n.s. | Intermediate | 0.84 | Generalist |
| *Australapatemon* cf. *burti* | 2 | 4 | 0.78 | -0.84 | n.s. | Intermediate | 0.75 | Generalist |
| **Telorchiidae** | | | | | | | | |
| *Opisthioglyphe ranae* | 2 | 2 | 0.36 | -0.97 | n.s. | Intermediate | 0.5 | Intermediate |

**References**

Aksenova, O.V., Vinarski, M.V., Itagaki, T., Ohari, Y., Oshida, T., Kim, S.K., Lee, J.H., Kondakov, A.V., Khrebtova, I.S., Soboleva, A.A., Travina, O.V., Sokolova, S.E., Palatov, D.M., Bespalaya, Y.V., Vikhrev, I., Gofarov, M.Y., Bolotov, I.N. (2014). Taxonomy and trans-Beringian biogeography of the pond snails (Gastropoda: Lymnaeidae) of East Asia: an integrative view. Zool J Linn Soc, 201, zlae083. https://doi.org/10.1093/zoolinnean/zlae083.

Bolotov, I.N., Aksenova, O.V., Bespalaya, Y.V., Gofarov, M.Y., Kondakov, A.V., Paltser, I.S., Stefansson, A., Travina, O.V., Viarski, M.V. (2017). Origin of a divergent mtDNA lineage of a freshwater snail species, *Radix balthica*, in Iceland: cryptic glacial refugia or a postglacial founder event?. Hydrobiologia 787, 73–98. https://doi.org/10.1007/s10750-016-2946-9.

Duan, Y., Al-Jubury, A., Kania, P.W., Buchmann, K. (2021). Trematode diversity reflecting the community structure of Danish freshwater systems: molecular clues. Parasites Vectors, 14, 43. https://doi.org/10.1186/s13071-020-04536-x.

Enabulele, E.E., Lawton, S.P., Walker, A.J., Kirk, R.S. (2018). Molecular and morphological characterization of the cercariae of *Lecithodendrium linstowi* (Dollfus, 1931), a trematode of bats, and incrimination of the first intermediate snail host, *Radix balthica*. Parasitology, 145, 307–312. https://doi.org/10.1017/S0031182017001640.

Enabulele, E.E., Lawton, S.P., Walker, A.J., Kirk, R.S. (2023). Molecular epidemiological analyses reveal extensive connectivity between *Echinostoma revolutum* (*sensu stricto*) populations across Eurasia and species richness of zoonotic echinostomatids in England. PLOS ONE, 18, e0270672. https://doi.org/10.1371/journal.pone.0270672.

Faltýnková, A., O'Dwyer, K., Pantoja, C., Jouet, D., Skírnisson, K., Kudlai, O. (2024). Trematode species diversity in the faucet snail, *Bithynia tentaculata* at the western edge of its native distribution, in Ireland. J Helminthol, 98, e52. https://doi.org/10.1017/S0022149X24000397.

Georgieva, S., Faltýnková, A., Brown, R., Blasco-Costa, I., Soldánová, M., Sitko, J., Scholz, T., Kostadinova, A. (2014). *Echinostoma* *'revolutum'* (Digenea: Echinostomatidae) species complex revisited: species delimitation based on novel molecular and morphological data gathered in Europe. Parasites Vectors, 7, 520. https://doi.org/10.1186/s13071-014-0520-8.

Georgieva, S., Selbach, C., Faltýnková, A., Soldánová, M., Sures, B., Skírnisson, K., Kostadinova, A. (2013). New cryptic species of the *'revolutum'* group of *Echinostoma* (Digenea: Echinostomatidae) revealed by molecular and morphological data. Parasites Vectors, 6, 64. https://doi.org/10.1186/1756-3305-6-64.

Gordy, M.A., Locke, S.A., Rawlings, T.A., Lapierre, A.R., Hanington, P.C. (2017). Molecular and morphological evidence for nine species in North American *Australapatemon* (Sudarikov, 1959): a phylogeny expansion with description of the zygocercous *Australapatemon mclaughlini* n. sp. Parasitol Res, 116, 2181–2198. https://doi.org/10.1007/s00436-017-5523-x.

Hüsken, A., Schwelm, J., Rückert, S., Sures, B. (2025). Intermediate insights: tracing trematodes infecting amphibians via their first intermediate snail hosts. Parasites Vectors, 18, 285. https://doi.org/10.1186/s13071-025-06920-x.

Hüsken, A., Schwelm, J., Sures, B. (2026). Land use drives trematode dynamics in a restored stream system. Curr Res Parasitol Vector Borne Dis 9, 100357. https://doi.org/10.1016/j.crpvbd.2026.100357.

Johnson, P.T.J., Calhoun, D.M., Achatz, T.J., Greiman, S.E., Gestos, A., Keeley, W.H. (2024). Outbreak of parasite-induced limb malformations in a declining amphibian species in Colorado. Int J Parasitol Parasit Wildl, 24, 100965. https://doi.org/10.1016/j.ijppaw.
2024.100965.

Katokhin, A.V., Kuzmenckin, D.V. (2020). Bithyniid Snails of Northern Eurasia. Unpublished.

Kudlai, O., Pantoja, C., O’Dwyer, K., Jouet, D., Skírnisson, K., Faltýnková, A. (2021). Diversity of *Plagiorchis* (Trematoda: Digenea) in high latitudes: Species composition and snail host spectrum revealed by integrative taxonomy. J Zool Syst Evol Res, 59, 937–962. https://doi.org/10.1111/jzs.12469.

Kundid, P., Pantoja, C., Janovcová, K., Soldánová, M. (2024). Molecular diversity of the genus *Plagiorchis* Lühe, 1899 in snail hosts of Central Europe with evidence of new lineages. Diversity, 16, 158. https://doi.org/10.3390/d16030158.

Menabit, S. (2021). Direct submission.

Nakao, M., Sasaki, M. (2021). Trematode diversity in freshwater snails from a stopover point for migratory waterfowls in Hokkaido, Japan: An assessment by molecular phylogenetic and population genetic analyses. Parasitol Int, 83, 102329. https://doi.org/10.1016/j.parint.2021.102329.

Pantoja, C., Faltýnková, A., O’Dwyer, K., Jouet, D., Skírnisson, K., Kudlai, O. (2021). Diversity of echinostomes (Digenea, Echinostomatidae) in their snail hosts at high latitudes. Parasite, 28, 59. https://doi.org/10.1051/parasite/2021054.

Pyrka, E., Kanarek, G., Gabrysiak, J., Jeżewski, W., Cichy, A., Stanicka, A., et al. (2022). Life history strategies of *Cotylurus* spp. Szidat, 1928 (Trematoda, Strigeidae) in the molecular era - Evolutionary consequences and implications for taxonomy. Int J Parasitol Parasit Wildl, 18, 201–211. https://doi.org/10.1016/j.ijppaw.2022.06.002.

Schniebs, K., Glöer, P., Vinarski, M., Hundsdörfer, A. (2016). A barcode pitfall in Palearctic Stagnicola specimens (Mollusca: Lymnaeidae): Incongruence of mitochondrial genes, a nuclear marker and morphology. North-West J Zool, 12, 239–254.

Schols, R., Smitz, N., Vanderheyden, A., Huyse, T. (2024). Expanding the swimmer's itch pool of the Benelux: a first record of the neurotropic *Trichobilharzia regenti* and potential link to human infection. Parasites Vectors, 17, 126. https://doi.org/10.1186/s13071-024-06218-4.

Schwelm, J., Kudlai, O., Smit, N.J., Selbach, C., Sures, B. (2020). High parasite diversity in a neglected host: larval trematodes of *Bithynia tentaculata* in Central Europe. J Helminthol, 94, e120. https://doi.org/10.1017/S0022149X19001093.

Soldánová, M., Georgieva, S., Roháčová, J., Knudsen, R., Kuhn, J.A., Henriksen, E.H., et al. (2017). Molecular analyses reveal high species diversity of trematodes in a sub-Arctic lake. Int J Parasitol, 47, 327–345. https://doi.org/10.1016/j.ijpara.2016.12.008.

Svinin, A.O., Chikhlyaev, I.V., Bashinskiy, I.W., Osipov, V.V., Neymark, L.A., Ivanov, A.Y., et al. (2023). Diversity of trematodes from the amphibian anomaly P hotspot: Role of planorbid snails. PLOS ONE, 18, e0281740. https://doi.org/10.1371/journal.pone.
0281740.

Vainutis, K., Andreev, M., Voronova, A., Zyumchenko, N. (2023). Direct submission.

Vinarski, M.V., Schniebs, K., Glöer, P., Hundsdörfer, A. (2011). The taxonomic status and phylogenetic relationships of the genus *Aenigmomphiscola* Kruglov and Starobogatov, 1981 (Gastropoda: Pulmonata: Lymnaeidae), J Nat Hist, 45, 2049–2068. http://dx.doi.org/10.1080/00222933.2011.574800.

Vinarski, M.V., Schniebs, K., Glöer, P., Nekhaev, I.O., Hundsdörfer, A. (2014). Geography rather than morphology explains the genetic diversity within the *Lymnaea stagnalis* s.l. complex (Mollusca, Gastropoda, Pulmonata) in Eurasia. Unpublished.

Zikmundová, J., Georgieva, S., Faltýnková, A., Soldánová, M., Kostadinova, A. (2014). Species diversity of *Plagiorchis* Lühe, 1899 (Digenea: Plagiorchiidae) in lymnaeid snails from freshwater ecosystems in central Europe revealed by molecules and morphology. Syst Parasitol, 88, 37–54. https://doi.org/10.1007/s11230-014-9481-8.
